# High-resolution binding data of TFIID and cofactors show promoter-specific differences

**DOI:** 10.1038/s41467-026-77758-9

**Published:** 2026-10-07

**Authors:** Sergio G-M Alcantara, Simon Bourdareau, Melanie Weilert, Julia Zeitlinger

**Affiliations:** 1 Stowers Institute for Medical Research, Kansas City, MO USA; 2 Department of Pathology & Laboratory Medicine, The University of Kansas Medical Center, Kansas City, KS USA

**Keywords:** Gene expression, Structural biology, Genomics, Transcription

## Abstract

TFIID is instrumental in recognizing promoter sequences and initiating transcription, yet a cohesive understanding of how this complex engages and functions at different promoter types in vivo is still lacking. Here, we employed ChIP-nexus to capture high-resolution binding footprints of all *Drosophila* TFIID subunits across the genome. These footprints reveal TFIID sub-modules whose DNA contacts agree with cryo-EM structures and point to new structural details. At different promoter types, the footprints of the TAFs are very similar, suggestive of the same engaged TFIID conformation. In contrast, the binding profile of TBP is promoter-specific, enabling us to identify TATA, DPR, and TCT/housekeeping promoters de novo, along with their underlying core promoter elements. Notably, TATA promoters display specific TBP and NC2 binding footprints and reduced TAF occupancy, consistent with a fraction of transcripts being initiated in a TAF-independent manner at these promoters in vivo. Together, our results provide a comprehensive resource for linking structural and biochemical results to in vivo data and offer a potential explanation for the increased burst size observed at TATA promoters.

## Abstract (web summary)

Here, the authors use ChIP-nexus to map all TFIID subunits and cofactors across the *Drosophila* genome at base pair resolution, revealing a general TFIID organization across promoter types with promoter-specific TBP and NC2 binding.

## Introduction

TFIID is a multi-subunit complex crucial for recognizing core promoter sequences, assembling the pre-initiation complex (PIC), and initiating RNA polymerase II (Pol II) transcription at transcription start sites (TSSs)^1^. TFIID is composed of TATA-binding protein (TBP) and 13 TBP-associated factors (TAFs), together forming a three-lobed structure when reconstituted in vitro^2^. As revealed by elegant cryo-EM studies, these lobes become reorganized when TFIID binds the promoter^3–6^. One lobe of TFIID binds to the downstream region (a.k.a. lobe C), while another lobe engages the upstream core promoter region (a.k.a. lobe B) together with the third lobe (a.k.a. lobe A), which is more flexible and structurally more difficult to characterize^5,6^. During this reorganization, TFIID places TBP near the site of transcription initiation, which is likely a critical step for PIC formation^5–9^.

Despite remarkable advances, TFIID is best understood in vitro, while its role in vivo across various promoter types remains incompletely understood. Different promoter types produce transcripts with distinct bursting properties, e.g. TATA promoters produce transcription bursts with more transcripts than TATA-less promoters^10–15^. Cryo-EM structures suggest that various promoter types are bound by engaged holo-TFIID^6^, but whether TFIID plays different functional roles across promoter types is not clear.

Since TFIID is very flexible in vitro and sometimes found in sub-stoichiometric compositions^16–20^, it is possible that TFIID preferentially binds some promoter types, adopts different conformations, or forms distinct sub-complexes. Alternatively, TFIID could influence the transcription initiation pattern by differential TBP mobilization. TBP’s contacts appear to be highly regulated since it is bound by several TAFs (TAF1, TAF11/13)^8^, as well as by NC2 and Mot1^21–27^. These observations raise the possibility that TFIID binding differs across promoter types, an idea that remains untested in vivo.

To investigate TFIID function in vivo, we performed ChIP-nexus experiments to map the occupancy of all 14 TFIID subunits at high-resolution across the *Drosophila* genome, in which core promoters are well characterized^28^. Our data reveal that TFIID’s DNA-bound modular organization is broadly conserved across promoters, but that TBP and NC2 display striking promoter-specific binding patterns. Notably, the TBP binding profile at TATA promoters suggests an additional mode of initiation that is independent of, or less reliant on, TAFs. We propose that this mechanism may contribute to the large transcription burst size of TATA promoters.

## Results

### ChIP-nexus captures all TFIID subunits at promoters

**Fig. 1:**
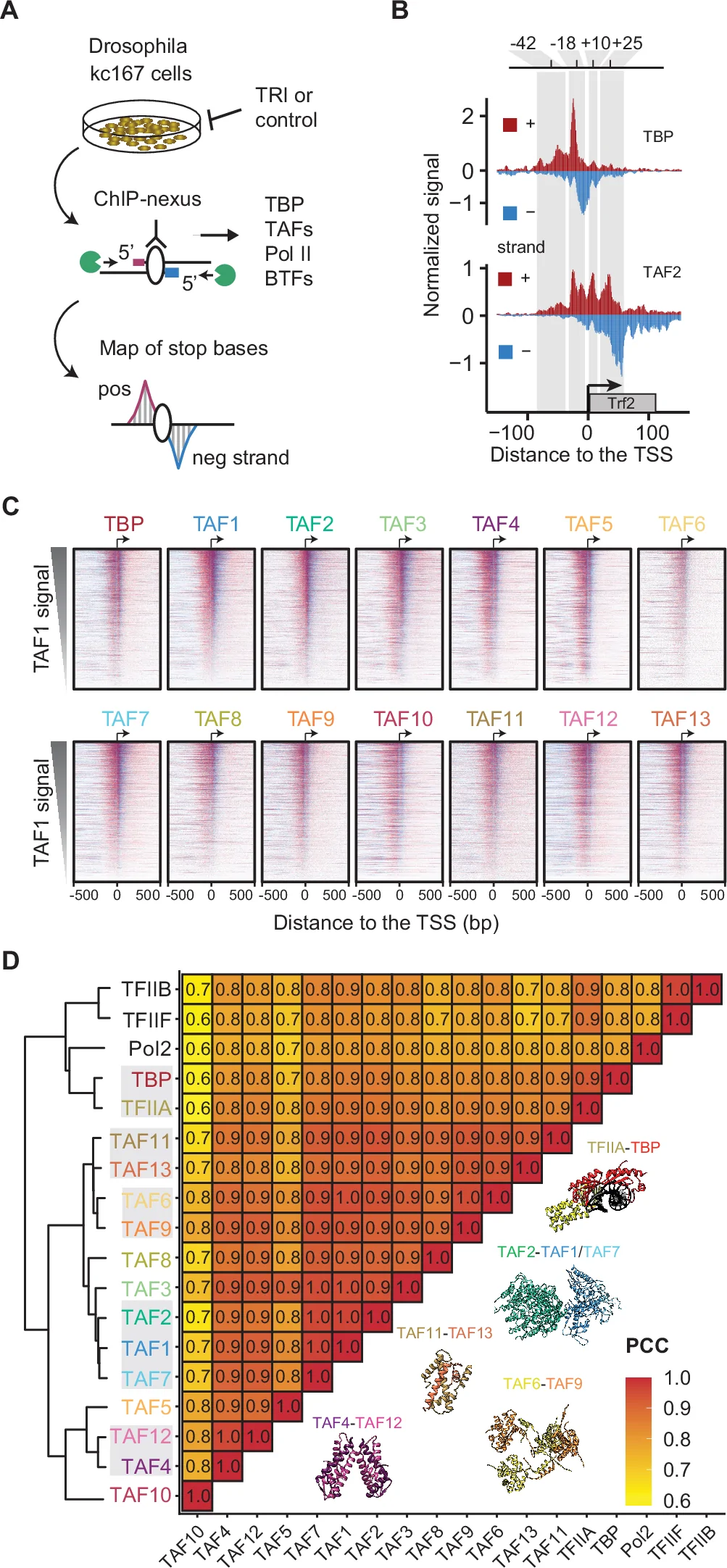
TFIID subunits show highly coordinated binding across active promoters. **A** Schematic representation of the ChIP-nexus workflow. **B** TBP and TAF2 binding profile at the *Trf2* gene, shown as normalized reads for each stop base on the positive strand (red) and the negative strand (blue). **C** Binding of each TFIID subunit across active promoters with narrow transcription initiation determined by CAGE-seq data (*n* = 1769). For each factor, the ChIP-nexus signal is shown for the positive (red) and negative (blue) strands, with the log intensity scaling from 0 to the maximum. Promoters were sorted based on the total binding of TAF1 within 100 bp centered on the TSS. **D** Pearson correlation coefficients (PCC) and hierarchical clustering of the binding of TFIID and PIC components across narrow promoters. The total binding signal was measured at each promoter within 100 bp centered on the TSS. Proteins known to physically interact (see structures) often clustered first (marked in gray). All protein structures were obtained from PDB: TFIIA-TBP (pdb_00001nvp), TAF1-7-2 (pdb_00006mzm), TAF4-12 (pdb_00001h3o), TAF6-9 (pdb_00006mzd) and TAF11-13 (pdb_00001bh8).

To robustly detect the patterns by which each TFIID subunit binds to core promoters in vivo at the highest resolution, we used ChIP-nexus^29^. ChIP-nexus is a chromatin immunoprecipitation coupled to exonuclease (ChIP-exo) technique, where a 5’ exonuclease digests the DNA of formaldehyde-fixed chromatin until encountering a protein-DNA crosslink. The DNA libraries produced from pulling down each TFIID subunit then show precise footprints, consisting of strand-specific peaks on either side of where the TFIID subunit is directly or indirectly crosslinked to DNA (Fig. 1A). We performed ChIP-nexus for all TFIID subunits in *Drosophila* Kc167 cells under normal conditions (DMSO control), and after treatment with triptolide (TRI), which blocks transcription at the PIC stage and, consistent with previous studies^30–33^, yields higher occupancy of PIC and TFIID components. After confirming that the biological replicate experiments were highly reproducible (Fig. S1), we analyzed the pooled ChIP-nexus coverage of each TFIID subunit. All experiments showed strong and distinct binding footprints of TFIID subunits at promoter regions (Fig. 1B).

We then chose active narrow promoters for our analysis since the positional information of high-resolution ChIP-nexus footprints is most interpretable when initiation occurs at a well-defined site. We used CAGE-seq data from Kc167 cell nuclear extracts (publicly available from modENCODE: ENCSR827COJ) ﻿and selected promoters where the annotated TSS shows transcription initiation within 11 bp (*n* = 1769; Fig.S2; see “Methods”). When the promoters were sorted by their TAF1 occupancy, the other TFIID subunits followed the same intensity pattern (Fig. 1C), suggesting that their binding levels are overall similar.﻿

We next clustered the TFIID subunits based on their binding level similarity across the promoters, measured as pairwise Pearson correlation coefficients (PCCs). We included ChIP-nexus data for the PIC transcription factors TFIIA, TFIIB, TFIIF and Pol II for comparison, which showed high correlations with the TFIID subunits (median PCC = 0.85) (Fig. 1D). Notably, TBP had the highest correlation with TFIIA (Fig. 1D), consistent with a direct physical interaction between TBP and TFIIA in the PIC^5,6,34,35^. Importantly, the TAFs were overall highly correlated in their binding levels (median PCC = 0.87), and the most correlated TAFs (TAF4/12, TAF1/7/2, TAF11/13 and TAF6/9) were also known to each form a physical complex^9^ (Fig. 1D). This suggests that high correlations are indicative of physical interactions, consistent with formaldehyde fixation generating abundant protein-protein crosslinks^36,37^ and causing interacting proteins to be pulled down with the same protein-DNA contacts in ChIP-nexus experiments^33,38–41^. Taken together, these results show that TFIID subunits exhibit highly coordinated binding across active promoters and suggest an in vivo organization that aligns with the known in vitro structure of TFIID.

### High-resolution footprints reveal the promoter-bound conformation of TFIID in vivo

To determine where TFIID subunits contact DNA, we analyzed the base-resolution ChIP-nexus binding footprints. Interacting proteins are often crosslinked to each other and pulled down together, thereby producing similar footprints in ChIP-exo/nexus data^33,38–41^. Exact protein-protein interactions cannot be inferred since formaldehyde crosslinks do not occur at all amino acids^37^, but as a cumulative measure, we expect the ChIP-nexus binding profiles to reflect the structural organization of TFIID in vivo.

**Fig. 2:**
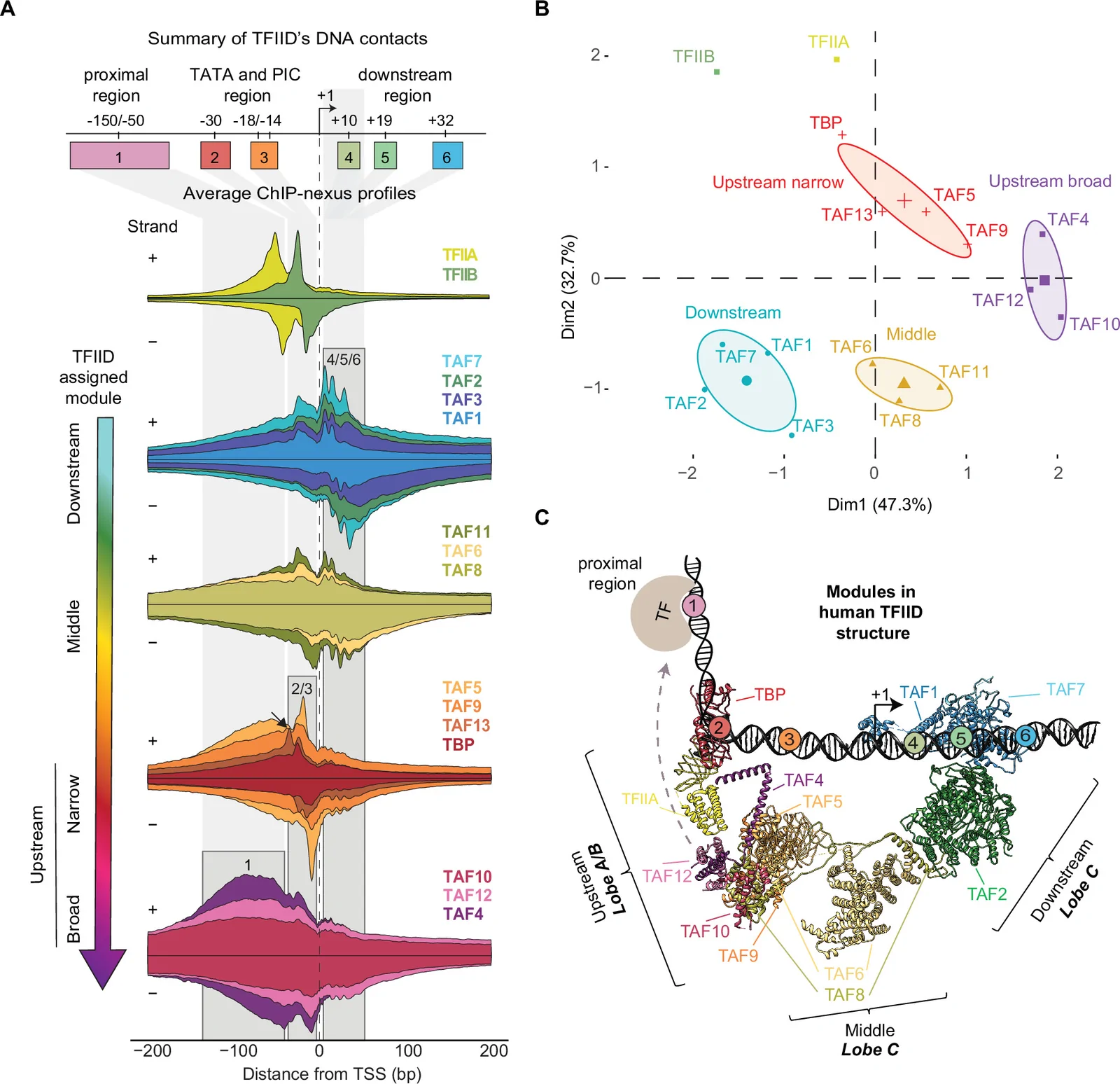
High-resolution ChIP-nexus footprints reveal a four-module organization of promoter-bound TFIID in vivo. **A** The average ChIP-nexus binding profile of the TFIID subunits centered at the TSS of narrow promoters (*n* = 1769) shows a four-module organization of the promoter-bound TFIID complex. For each subunit, the binding profile on the positive strand (top line) and negative strand (bottom line) are shown in the same color and stretched to an arbitrary scale to facilitate comparison with other subunits. The black arrow points to the footprint of TAF13 at −30. **B** Principal Component Analysis (PCA) of the averaged ChIP-nexus profiles confirms the modular organization. Modules were defined based on PCA clustering and similarity in binding profiles. **C** Cryo-EM structure of human promoter-bound TFIID (PDB: pdb_00006mzm) is consistent with the modular organization and DNA contacts identified by the ChIP-nexus experiments. The human TFIID structure is derived from the synthetic Super Core Promoter (SCP) template^3^, which contains TATA, Inr, MTE, and DPE elements, all of which are enriched in the narrow promoter set selected for the analysis in (**A**).

To analyze where the DNA contacts occur for each factor, we used the ChIP-nexus data of TBP and the 13 TAFs after triptolide treatment and averaged across the promoters with narrow transcription initiation (Fig. 2A). As expected, promoters with broad transcription initiation exhibited substantially more diffuse average profiles (Fig. S3). Since ChIP-nexus produces strand-specific peaks on either side of the protein-DNA crosslink site, we calculated the midpoint between matching peaks on the positive strand and negative strand to identify the protein-DNA contacts in each average profile (see Fig. S3 left). We note that the DNA-bound organization of TFIID analyzed here reflects an aggregate pattern across diverse promoter classes (Fig. S2C).

The preferred DNA contacts, as well as the modular organization, align well with cryo-EM structures of promoter-bound human and yeast TFIID (Fig. 2C)^3–6^. The main footprint positions were found in six distinct regions, mapped at −150/−50, −30, −18/−14, +10, +19 and +32 bp relative to the TSS (Regions 1–6 in Fig. 2A). In agreement with Principal Component Analysis (PCA) of the average profiles, we manually grouped the 14 TFIID subunits into four modules, which we call the downstream, middle, upstream narrow and upstream broad modules (Fig. 2A, B). TFIIA and TFIIB were separated from the TAF modules, but closer to TBP in PCA space, consistent with their TBP association in the PIC. In the untreated (DMSO) condition, similar TFIID modules were observed (Fig. S4A, B), indicating that they are not specific for triptolide treatment. We then analyzed our data to obtain insights into the promoter-bound organization of TFIID in vivo.

Our downstream module is the lobe C that engages with the downstream promoter region (DPR). Here, TAF1, TAF7, and TAF2 form three clustered footprints at +10, +19, and +32 bp (Fig. 2A, regions 4–6), consistent with the human TFIID cryo-EM structures^3,5,6^ and contradicting previous biochemical evidence implicating *Drosophila* TAF6/9 in binding to DPR elements^42^. Interestingly, TAF3 is also included in our downstream module. TAF3 is part of the flexible lobe A, whose location in promoter-bound TFIID is not well established^5,6^, but the proximity of TAF3 near TAF1/2/7 is supported by crosslinking mass spectrometry analysis in yeast^4^. The presence of TAF3 in this module should allow its PHD finger to interact with the H3K4me3 mark of the +1 nucleosome downstream of the TSS^9,43,44^, though we note that the levels of TAF3 (and TFIID in general) do not correlate well with the levels of H3K4me3 (Fig. S4C).

The middle module shows footprints both upstream and downstream of the TSS (Fig. 2A, regions 2–5). As expected, it contains TAF6 and TAF8, which are known to bridge the upstream and downstream module in cryo-EM structures and thus indirectly contact the promoter both upstream and downstream (Fig. 2C). More unexpectedly, TAF11 clusters within the middle module, even though TAF11 forms a heterodimer with TAF13, which is found in the upstream module^8^. TAF11/13 are known to interact with TBP and inhibit its binding to DNA in the canonical state of TFIID. Thus, in the conformation of TFIID where TBP engages the promoter, TAF11/13 must relocate or disengage from TBP^5,6^. One possible explanation is that the TAF11/TAF13 heterodimer adopts a flexible or repositioned configuration upon TBP engagement, allowing TAF11 to shift closer to the middle module while TAF13 remains positioned upstream.

The narrow upstream module consists of TBP, TAF5, TAF9, and TAF13, which are from the two lobes that engage the upstream promoter region (lobe A and B). The density for these TAFs is poorer in cryo-EM structures^4–6^, but their overall location agrees with our data. This module shows the strongest footprints around the site of PIC formation at −18/−14 bp (Fig. 2A, regions 2 and 3). This position seems to be a broader crosslinking hotspot in our data, since all TFIID subunits, as well as TFIIA and TFIIB, show a footprint at this location. This likely reflects the dense network of interactions within the engaged PIC at this region.

Notably, the strongest footprint of TBP is found at the −18 bp position. While this is consistent with previous *Drosophila* TBP ChIP-nexus data and human TBP ChIP-exo data^33,38,39^, it is puzzling since the TATA box is found at -30 bp, and according to cryo-EM structures^6^, TBP binds a similar position at TATA-less promoters. This is unlikely due to a general lack of crosslinking at this position since TAF13 has a strong footprint there (Fig. 2A, arrow). Rather, the limited crosslinking efficiency may reflect a feature specific for how TBP contacts DNA. TBP interacts with DNA primarily through the minor groove, by inserting conserved phenylalanine residues between TATA bases^45^. Formaldehyde crosslinking depends however on the proximity of amine groups in the protein and accessible reactive bases in the DNA^37^,  both of which might not be readily available when TBP binds to the TATA box.

Finally, we identified a broad upstream module that exhibits high levels of diffuse footprints upstream of the TSS (Fig. 2A, region 1). This module comprises TAF4, TAF10 and TAF12, each present in two copies and belonging to the two lobes that engage the upstream promoter region^3,5,6^. These diffuse footprints may result from interactions with promoter-proximal upstream enhancers, as many TAFs are known to associate with DNA-bound transcription factors^46–54^. Specifically, TAF4 has been identified as an interaction hub for transcription activators in biochemical studies^55,56^, supporting the idea that broad upstream footprints may reflect activator-mediated stabilization of TFIID. Additionally, TAF10 and TAF12 could engage these regions as part of the SAGA complex^57^.

Taken together, the ChIP-nexus profiles show strong overall agreement with the in vitro cryo-EM structures, provide insights for less well-characterized subunits such as TAF3, and point to unexplained observations for TAF11/13 and TBP.

### The TBP binding profile is promoter-type-specific

**Fig. 3:**
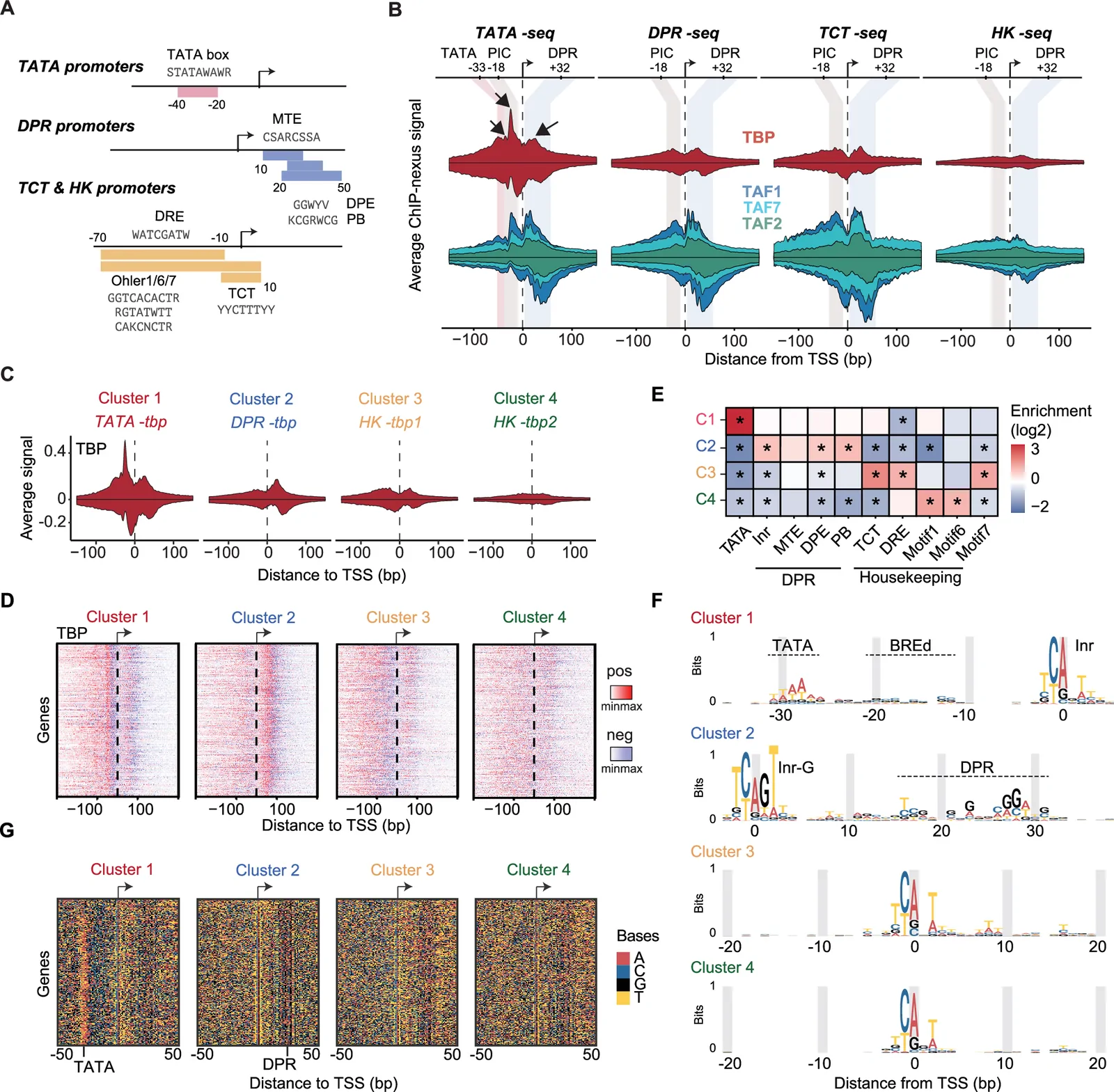
TBP is promoter-specific and allows the de novo identification of promoter types. **A** Schematic summarizing the different promoter elements used for analysis (see Methods and Supplementary Table 1 for more information). **B** The average ChIP-nexus binding profile of TBP (top; red), TAF1, TAF2 and TAF7 (second row) at TATA (*n* = 191), DPR (*n* = 334), TCT (*n* = 78) and HK (*n* = 282) promoters. Promoter classes are labeled with a “–*seq*” suffix to indicate sequence-based classification. **C** Average ChIP-nexus binding profiles of TBP at the four different k-means clusters (cluster 1, *n* = 309; cluster 2, *n* = 509; cluster 3, *n* = 392; cluster 4, *n* = 382) labeled with a “–*tbp*” suffix to indicate classification based on TBP binding patterns. **D** Distribution of TBP binding location at all promoters within each promoter cluster. Binding to the positive and negative strand is shown in red and blue, respectively. Signal per TSS was normalized to cluster promoters based on the TBP profile shape (see “Methods”). **E** Promoter clusters are enriched for different core promoter elements. Statistical significance was assessed using a two-sided Fisher’s exact test with Benjamini–Hochberg correction, *p < 0.05. Full statistical details are reported in Supplementary Table 2. **F** Position weight matrix (PWM) of the identified upstream, initiator and downstream regions of the four identified promoter clusters. **G** Underlying sequence information contents across the promoter clusters colored by base.

The high correlation of the TFIID subunits across promoter types argues against partial TFIID complexes at specific promoters but does not rule out differences by which TFIID subunits interact with distinct promoter types. To test this, we analyzed our ChIP-nexus data from untreated cells to capture all possible promoter states (see Fig. S6A for data from triptolide-treated cells). We identified four representative promoter types among our defined set of narrow promoters, selected based on the presence of core promoter elements at specific positions relative to the TSS, as done previously^33,58,59^ (Figs. 3A and S5A, B; see “Methods”).

TATA promoters (*n* = 191) have a TATA box located -30 bp upstream of the TSS. DPR promoters (*n* = 334) have a DPR element at +10–50 bp, thus an MTE, DPE or PB motif. DPR promoters typically show higher levels of Pol II pausing^33,59,60^. TCT promoters (*n* = 78) initiate at a TCT motif and are often found at ribosomal genes^61,62^. Finally, housekeeping (HK) promoters (*n* = 282) have at least one of the known housekeeping core promoter elements (DRE, Ohler 1, 6 and 7 motifs) and no TATA, TCT or DPR element. HK promoters tend to have broader initiation windows^63–66^, but this dispersion was kept minimal by our selection of promoters with narrow transcription initiation.

Comparing the profiles of all TFIID subunits at the four promoter types revealed that TBP has the most promoter-specific profile (Fig. 3B), while most TAFs display broadly similar profiles across promoter classes, with only subtle differences. For example, the TAF binding profiles at TCT and HK promoters tend to be more diffuse with more pronounced upstream binding (Fig. S6A). Since these promoter types have their regulatory regions very closely nearby^63^, this upstream binding could be the result of TFIID interacting with transcription activators. Furthermore, TRF2 occupancy was minimal at TATA promoters and highest at TCT promoters (Fig. S6A), in line with previous observations^61,62,65,67–69^. Overall, TBP has the most intriguing promoter-specific profile, because it supports the hypothesis that TBP is a mobile component of TFIID^1,9,70^ and may indicate functional differences between promoter types.

The most notable feature is that the strong footprint of TBP at the upstream −18 bp position is specific for TATA promoters (Fig. 3B). It is the strongest after triptolide treatment, supporting its association with the PIC (Fig. S6A). TBP also displays a broad footprint +32 bp downstream of the TSS (Fig. 3B). This footprint has been previously observed^33^ and is present at all promoter types to various degrees, but it is not observed after triptolide treatment (Figs. 2A and S6A) and has lower levels at promoters with stably paused Pol II (Fig. S9). This suggests that downstream TBP occurs during a yet unknown step of the transcription cycle. Since cryo-EM studies have suggested that TBP is in a promoter-downstream position before it gets loaded onto the upstream position^3,5,6^, it is possible that downstream TBP represents the TFIID loading or re-loading state.

While TBP appears to have the most promoter-specific profile, this conclusion is so far based on average profiles across selected promoter sets. To test whether the differential TBP binding profile is robust across individual promoters, we asked whether we could re-classify promoters based on their individual TBP binding profile. Using the base-resolution TBP profiles at the previously defined narrow promoters, we performed k-means clustering allowing four possible clusters (see Methods).

The TBP profiles of the four clusters (Fig. 3C, D) and their enrichment of core promoter elements (Fig. 3E) suggest that the clusters represent TATA promoters (cluster 1), DPR promoters (cluster 2), and two sets of TCT/HK promoters, one with diffuse TBP ChIP-nexus footprints (cluster 3), and the other with low TBP ChIP-nexus footprints (cluster 4) (Fig. 3C, D). Notably, the TBP levels correlated with the mRNA levels measured by RNA-seq and CAGE-seq (Fig. S5C, D).

As further validation, we asked whether we could de novo discover the core promoter elements from each ﻿of the promoter sets clustered by TBP profile (Fig. 3F, G). We aligned the sequences at the TSS and visualized the bases as a color-coded heatmap. Due﻿ to their strict positioning relative to the TSS, certain core promoter elements were discernible by eye (Fig. 3G). Clusters 3 and 4 (TCT/HK) showed clear Inr elements, while clusters 1 and 2 showed additional strong sequence patterns indicative of TATA and DPR promoters, respectively (Fig. 3G). Consistently, the resulting motif logos closely matched the known core promoter elements^71–73^ (Fig. 3F). Notably, the TATA and DPR promoters both have Inr elements, but only those from DPR promoters were enriched for the G at position +2 (Inr-G) as previously reported^59^. Furthermore, a CG-rich sequence next to the TATA motif resembles the TFIIB response element (BREd)^74^, but we cannot exclude that this sequence simply enhances the crosslinking of TFIIB at this position. Taken together, we conclude that TBP displays binding profiles that can be used to classify promoter types de novo and predict their sequence elements.

### TATA promoters show high occupancy of TBP and NC2 with reduced TAF occupancy

We next investigated what causes the strong upstream footprint of TBP at TATA promoters. We considered that it might be due to prolonged binding of TBP at the TATA box, which could explain more efficient re-initiation at TATA promoters^75–79^. If so, one would expect higher steady-state levels of TBP relative to the transcription output at TATA versus DPR promoters. To test this, we normalized the ChIP-nexus binding profiles of TBP and the other factors by dividing the binding signal at each promoter by the corresponding nuclear CAGE-seq transcript levels. This ratio cannot infer initiation kinetics but provides an approximate measure of promoter occupancy relative to transcription initiation.

**Fig. 4:**
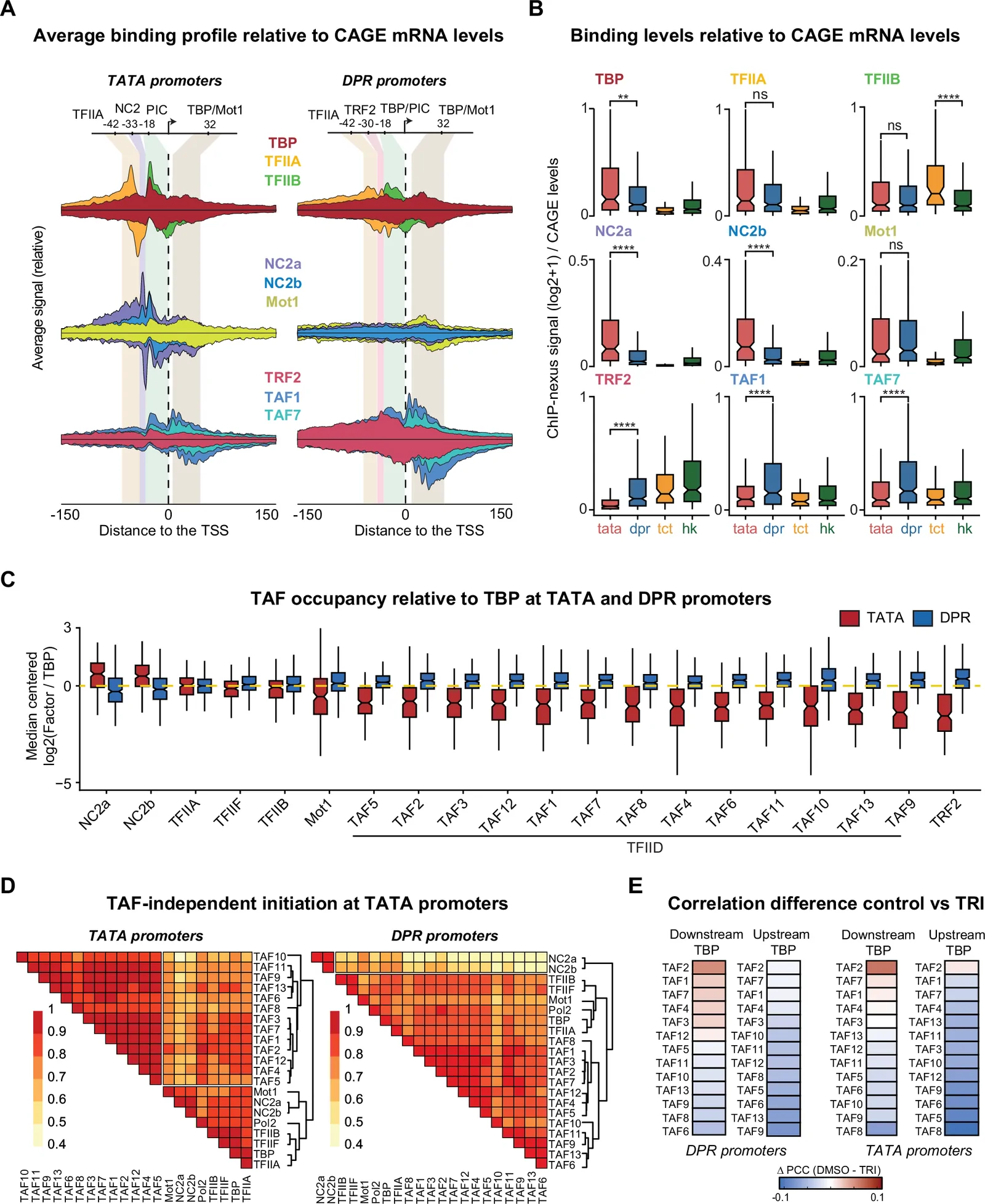
TATA promoters show reduced occupancy of TAFs. **A** Average transcription-normalized binding profile of TBP, TFIIA and TFIIB (top row; red, yellow and green), NC2α, NC2β and Mot1 (middle row; purple, blue and yellow-green), and TRF2, TAF1 and TAF7 (bottom row; pink, blue and turquoise) at TATA (*n* = 191) and DPR (*n* = 334) promoters. Transcription normalization was calculated by dividing the bp signal at each promoter by its corresponding CAGE-seq score. **B** Transcription-normalized signal distribution for the transcription factors shown in (**A**) at the same TATA and DPR promoters, together with TCT (*n* = 78) and HK (*n* = 282) promoters. Normalized occupancy was calculated by dividing the total ChIP-nexus signal (measured using factor-specific windows around the TSS, see “Methods”) at each promoter by the corresponding CAGE-seq levels. Statistical significance was assessed using two-sided Wilcoxon rank-sum tests with Benjamini–Hochberg correction: ns (not significant), *p* ≥ 0.05; **, *p* < 0.01; ****, *p* < 0.0001. Exact *p* values, effect sizes and 95% confidence intervals are reported in Supplementary Table 3. **C** Occupancy distribution of each factor relative to TBP at TATA and DPR promoters. Relative ChIP-nexus occupancy was calculated as the log2-transformed ratio between the total signal of each factor and the total TBP signal, measured within a 100-bp window centered on the TSS. For each factor, relative occupancy ratios were median-centered by subtracting the median log2(*factor*/TBP) value across promoters. For panels B and C, the central line indicates the median, the box spans the 25th–75th percentiles, and whiskers extend to the most extreme values within 1.5 × the interquartile range. Notches indicate the approximate 95% confidence interval around the median. Values beyond the whiskers are not shown but were included in the statistical analyses. **D** Pairwise Pearson correlation coefficients for the promoter-level occupancy of TFIID, TBP, PIC, NC2, and Mot1 at TATA and DPR promoters. **E** Difference in Pearson correlation coefficients between control (DMSO) and triptolide (TRI) when correlating upstream or downstream TBP with TAFs. Results are shown for TATA and DPR promoters, and differences were calculated as ΔPCC = PCC(DMSO) − PCC(TRI).

Once normalized, the levels of TFIIA, TFIIB and TFIIF were not statistically different between TATA and DPR promoters (Figs. 4A, B and S7), suggesting that PIC factor occupancy is roughly proportional to the amount of transcripts initiated at these promoters. An exception was TCT promoters, which had higher levels of TFIIB, indicating functional differences (Fig. 4B). For TBP, we analyzed the binding levels upstream of the TSS because the downstream signal was not associated with the PIC. This revealed that TBP binding was significantly higher at TATA promoters than at DPR promoters (Fig. 4B). Thus, whereas other PIC components largely scaled with the transcription output, TBP showed elevated binding levels at TATA promoters. These observations suggest that TBP may persist longer at the TATA box, potentially facilitating PIC reassembly during transcription reinitiation.

We next investigated whether the strong upstream TBP footprint at TATA promoters could be associated with reduced TAF occupancy. This is in agreement with classic studies showing that TATA promoters do not require TAFs for transcription initiation in vitro^80–86^, as well as with in vivo studies showing that TATA promoters have reduced TAF occupancy and a lower dependency on TAFs for transcription^16,38,87–92^. When we analyzed the transcript-normalized levels of TFIID, we found that all TAFs showed significantly lower levels at TATA promoters, while TBP, TFIIA, TFIIB and TFIIF were nevertheless present at similar or higher levels (Figs. 4A, B and S7). To make sure that this conclusion was not dependent on the normalization, we confirmed that the expected relative ratios of TBP and TAF binding were also present in the non-normalized data (Figs. 4C and S6B). These data together show that TATA promoters have substantially lower occupancy of TAFs and support the hypothesis that to some extent, TBP can initiate PIC formation from the TATA box without TFIID in vivo.

We then examined whether other TBP-associated proteins might provide clues to TBP’s promoter-specific distribution. We performed ChIP-nexus experiments on Mot1 and both NC2 subunits, two factors known to regulate TBP binding on DNA with mostly negative effects on transcription^21,23,24,26,93^. Mot1 showed elevated binding at all locations where TBP binds at TATA and DPR promoters, especially at the downstream footprint (Figs. 4A and S8A). In contrast, both NC2 subunits, NC2α and NC2β, were specifically bound to TATA promoters, with significantly higher occupancy compared to  DPR promoters even after normalization (*p* < 0.001) (Fig. 4A, B). Each NC2 subunit showed two sharp ChIP-nexus footprints at TATA promoters: one at −18 bp, coinciding with that of TBP, and another at −33 bp near the TATA box (Figs. 4A and S8A). These results match remarkably well the crystal structure of the TBP-NC2-DNA ternary complex where NC2α contacts the TATA box underneath TBP (Lys18, Lys19) and NC2β also interacts with the downstream region (Lys63, Lys64)^94^ (Fig. S8B).

Finally, we performed additional analyses to support the differences in binding patterns between TATA and DPR promoters. Because TATA promoters show strong NC2 binding and reduced TAF occupancy, we reasoned that with comparable PIC levels and transcription output, promoters with high NC2 should still be associated with higher TBP and lower TAF binding than promoters with low NC2, and this was indeed the case (Fig. S8C–E). Moreover, to obtain additional evidence not based on binding differences between promoter types, we analyzed each promoter type separately and performed a pairwise correlation analysis between the binding levels of TBP, TAFs, TFIIA, TFIIB, TFIIF, NC2 and Mot1 (Fig. 4D). At TATA promoters, TBP shows stronger correlations with TFIIA, TFIIB, TFIIF, NC2 and Mot1 than with the TAFs, implying that TBP does not always co-occur with the TAFs (Fig. 4D, left). This pattern was not observed at DPR promoters, where the binding levels of all factors were more homogenous, consistent with TFIID being an integral part of the machinery mediating PIC formation at DPR promoters (Fig. 4D, right). Taken together, these results support a model in which TATA promoters do not initiate all rounds of transcription through TFIID. Instead, relatively stable TBP binding at the TATA box may enable PIC formation independently of TFIID, a mechanism that may be antagonized by NC2.

Interestingly, this conclusion also sheds light on the binding of TBP downstream. TATA promoters not only have lower levels of TAFs but also lower levels of downstream TBP compared to its upstream binding. In contrast, DPR promoters whose downstream sequences favor TFIID binding have higher levels of TBP downstream than upstream. This suggests that downstream TBP is associated with TFIID and thus may be part of the TFIID loading state. If so, we wondered whether we could identify TAFs that correlate more highly with the TFIID loading state and thus might be physically close to TBP when downstream. For this, we took advantage of the fact that TBP is found downstream under steady-state control conditions, but not after triptolide treatment. We asked which TAFs have the highest increase in correlation with downstream TBP under control *versus* triptolide conditions. We found TAF2 to have the greatest increase in correlation, followed by TAF1 and TAF7, and these increases were overall higher at DPR promoters versus TATA promoters (Fig. 4E), consistent with the downstream module mediating the loading of TBP. We would have expected TAF1 to have the highest signal since it can contact TBP directly through its TAND domain, but it is possible that the DNA-facing surface of TAF2 is more easily cross-linked to DNA than TAF1.

**Fig. 5:**
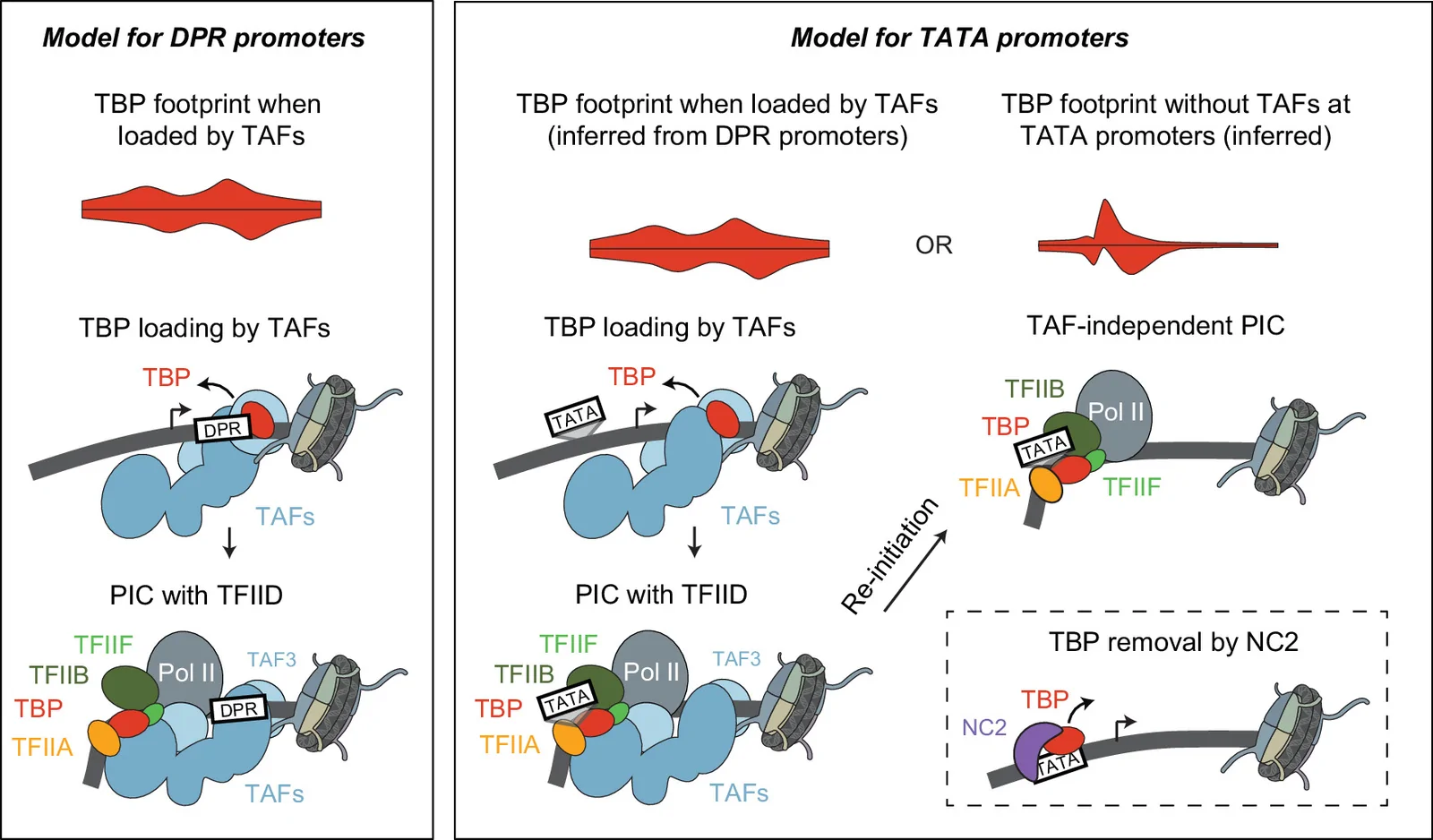
Model for transcription initiation at TATA and DPR promoters. The model summarizes the promoter-dependent modes of TBP recruitment and PIC assembly inferred from the data. At DPR promoters (left), TBP is recruited as part of TFIID through interactions of TAFs with downstream core-promoter sequences. This mode of recruitment produces the characteristic downstream TBP footprint shown at the top. Following TFIID engagement, TBP is positioned upstream, where TFIIA, TFIIB, TFIIF and Pol II assemble to form the PIC. At TATA promoters (right), two alternative modes are proposed. In the first, TBP is loaded through TFIID, analogous to the mechanism inferred at DPR promoters. Following loading by TFIID, TBP may remain bound longer at the TATA box and support successive rounds of re-initiation through a second mode that functions independently of TAFs. In addition, it is possible that TBP occasionally binds directly to the TATA box and mediates initiation without TAFs. The presence of TAF-independent PICs at TATA promoters may explain the sharply localized TBP footprints upstream of the TSS and the reduced TAF occupancy observed at these promoters. The dashed box illustrates a potential mechanism for terminating or resetting this TAF-independent promoter state. NC2 associates strongly with promoter-bound TBP at TATA promoters and may antagonize prolonged TBP binding and TAF-independent PIC formation. The TBP footprints shown above the proposed pathways represent the characteristic patterns observed experimentally and illustrate the different modes of TBP engagement inferred at DPR and TATA promoters. Together, these promoter-specific pathways provide a model in which DPR promoters predominantly use TFIID-mediated TBP loading, whereas TATA promoters can additionally exploit persistent or directly recruited TBP to support repeated PIC assembly. Such alternative modes of initiation could facilitate more efficient transcriptional re-initiation and thereby contribute to the larger transcription burst sizes characteristic of TATA promoters.

These findings suggest a model that explains the strong upstream TBP footprint at TATA promoters and the higher downstream footprint at DPR promoters. At both promoter types, TFIID initiates transcription by relocating TBP from the downstream to the upstream promoter position (Fig. 5). At TATA-containing promoters, however, TBP could either directly bind to the TATA box and mediate PIC formation, or promote TAF-independent transcription re-initiation after being initially loaded by TFIID.

## Discussion

While cryo-EM studies have unraveled the structure of TFIID bound to specific promoters in yeast and humans, it has remained speculative how TFIID’s structure and function might differ between promoter types. By mapping all 14 TFIID subunits at base-pair resolution, we provide a comprehensive view of how TFIID binds across different promoter types in vivo. Our data are remarkably consistent with cryo-EM structures and suggest that the holo-TFIID complex is found at active promoters, in line with recent functional and structural investigations^6,95–97^. However, we found that TBP displays significant promoter-specific differences, allowing us to connect structural studies with previous observations from biochemistry and genetics and construct a model of the function of TFIID at different promoter types.

Our in vivo ChIP-nexus profiles support a distinct alternative mechanism of initiation at TATA promoters. Through various analyses, we conclude that TATA promoters have elevated TBP binding but significantly lower TAF occupancy relative to PIC levels and transcription output. These findings are consistent with TATA promoters producing a substantial fraction of transcription in a TAF-independent manner^87^. Whether TFIID is universally required for transcription initiation and the extent to which this requirement depends on promoter type have been debated for long^38,81,87–92,96,98^. Early biochemical studies demonstrated that basal transcription from TATA promoters can be supported in vitro by recombinant TBP together with the general transcription factors in the absence of TAFs^80–86^. Consistent with these observations, subsequent genetic experiments in yeast and *Drosophila* showed that TAF-dependent and TAF-independent forms of transcriptionally active TBP can exist on promoters^16,89–92^. However, later work challenged a strict division between TAF-dependent and TAF-independent promoters and suggested that TFIID contributes broadly to transcription^25,81,96,98^. In particular, genome-wide perturbation experiments in yeast showed that rapid depletion of TAFs widely reduces transcription from both TATA and TATA-less genes, though a subset of TATA-rich promoters were less dependent on TAFs^81,96,98^. Conversely, parallel studies in yeast and mammals provided strong evidence that transcription of TATA genes persists upon depletion of certain TAF subunits^43,87^, and that TBP and the PIC are bound at active TATA promoters even when TAFs show minimal occupancy^38,88^. Because these studies relied on individual or limited subsets of TAFs as proxies for the entire TFIID complex, our high-resolution in vivo analysis of the complete set of TAFs extends and helps reconcile previous observations, supporting a model in which TFIID contributes to the transcription of most genes, while PIC formation at TATA promoters can occur to some extent in the absence of TAFs.

Our high-resolution profiles also provide more conclusive information on the promoter-specific binding of NC2 and TRF2. TRF2’s binding was particularly low at TATA promoters and higher at DPR, HK, and TCT promoters, in agreement with previous studies^61,67,68,99–101^. While it is unclear how TRF2 relates to TFIID, they both seem to preferentially function at TATA-less promoter types. In contrast, NC2 showed strong, sharp ChIP-nexus footprints specifically at TATA promoters, but not at TATA-less promoters. These footprints agree with structural data of the TATA-TBP-NC2 ternary complex^94^ and provide indirect evidence that TBP strongly binds at the TATA box, although its strongest footprint is detected slightly more downstream at -18 bp. The specificity of NC2 for TATA genes suggests that NC2 primarily inhibits transcription at TATA genes and that effects on TATA-less promoters^24,25,102–105^ may be largely indirect. Since previous evidence suggests that NC2 prevents PIC assembly and destabilizes TBP binding^24,94^, our data support a model in which NC2 antagonizes the prolonged binding of TBP at TATA promoters.

A more puzzling observation is that under steady-state conditions, all promoters with TFIID show TBP binding downstream of the TSS^33^. This downstream binding is most strongly associated with TAF2 at DPR promoters and is relatively lower at TATA promoters, where the TAF occupancy is also lower, suggesting that downstream TBP is part of TFIID. While we cannot exclude alternative explanations, several observations point to downstream TBP being part of the DNA-bound loading or reloading state of TFIID. Downstream TBP is not observed after triptolide treatment, when the DNA-binding contacts suggest that TFIID is preferentially in a PIC state and thus would sterically hinder other conformations of TFIID. Downstream TBP is also reduced at promoters with relatively stable Pol II pausing, either due to steric occlusion as well or because these promoters are less active. Given that cryo-EM structures suggest that the downstream position corresponds to the site where TFIID initially binds DNA to place TBP upstream of the promoter^5^, we favor the hypothesis that downstream TBP reflects the DNA-bound loading state of TFIID.

In light of these findings, our results inform models for the larger transcription burst sizes observed at TATA promoters^10–12,14,58,106,107^. By having alternative modes of transcription initiation, TATA promoters may allow more efficient reloading of TBP during a burst. TBP may either bind the TATA box directly to promote PIC formation, or TFIID could initially load TBP and subsequently disengage, while TBP remains promoter-bound. Such prolonged binding of TBP at TATA promoters is supported by the higher TBP occupancy relative to other PIC components, as well as recent live-cell single-molecule tracking experiments in yeast showing that TBP has longer residency times (~28 s) than RNA Pol II and PIC factors, which are short-lived (~2–5 s)^108^. Thus, once TBP is loaded onto the TATA box, it could remain bound through multiple rounds of transcription, as suggested by previous studies^75–79^. Alternatively, TBP may remain chromatin-bound but must re-bind the TATA box for each round of transcription since recent structural studies in vitro suggest that Pol II elongation leads to TBP being evicted from the TATA box^109^. In either case, TBP’s long residence time and ability to initiate PIC formation from the TATA box likely explain the larger burst size of TATA promoters. Altogether, our high-resolution mapping of TFIID binding and other co-factors provides a resource for refining models of promoter-specific transcription initiation.

## Methods

### Statistics and reproducibility

ChIP-nexus experiments were performed in at least two independent biological replicates for each factor and treatment condition (except non-treated Mot1, which has only one replicate). Replicate reproducibility was assessed using Pearson correlation of promoter-level binding signals (Fig. S1). RNA-seq experiments were performed in two biological replicates in DMSO (control) condition. Publicly available CAGE-seq data from *Drosophila* melanogaster Kc167 cell nuclear extracts were derived from two biological replicates. Publicly available H3K4me3 ChIP-seq data generated on *Drosophila* melanogaster Kc167 cells were derived from one replicate.

The number of promoters included in each analysis is reported in the corresponding figure legend. No statistical method was used to predetermine sample size and no data were excluded from the analyses. Extreme values were omitted only from box-plot displays to improve visualization, but were retained for the statistical tests.

Fu statistical tests, exact values are reported in the corresponding Supplementary Tables 2-7. PCCs were used to assess similarity between promoter-level binding profiles.

All statistical analyses were performed in R, and the analysis code is publicly available at the GitHub repository specified in the Code Availability statement.

### Cell culture and transcription inhibitor treatment

*Drosophila* melanogaster Kc167 cells (purchased from DGRC; negative for mycoplasma contamination) were grown at 25 °C in HyClone SFX-Insect Cell Culture Media. Transcription inhibitors were added directly into SFX media. Cells were treated with 10 μM triptolide (TOCRIS Bioscience, 3253; dissolved in DMSO) at room temperature for 1 h. Equivalent amounts of DMSO treatment (2%) were used as the control. The identity of the cell line was not independently authenticated in this study. Kc167 is not listed in the International Cell Line Authentication Committee Register of Misidentified Cell Lines (version 14, accessed 20 July 2026).

### ChIP-nexus experiments

For each ChIP-nexus experiment, 10 million Kc167 cells were fixed with 1% formaldehyde in culture media at room temperature for 10 min with rotation. Fixed cells were washed with cold PBS, incubated with Orlando and Paro’s Buffer (0.25% Triton X-100, 10 mM EDTA, 0.5 mM EGTA, 10 mM Tris-HCl pH 8.0, with freshly added Protease Inhibitor) for 10 min at room temperature with rotation, and then centrifuged and re-suspended in ChIP Buffer (10 mM Tris-HCl, pH 8.0; 140 mM NaCl; 0.1% SDS; 0.1% sodium deoxycholate; 0.5% sarkosyl; 1% Triton X-100, with freshly added Protease Inhibitor). Sonication was performed with a Bioruptor Pico for five cycles of 30 s on and 30 s off at HIGH setting. Chromatin extracts were then centrifuged at 16,000 × *g* for 5 min at 4 °C, and supernatants were used for ChIP.

To couple Dynabeads with antibodies, 50 µl Protein A and 50 µl Protein G Dynabeads were used for each ChIP-nexus experiment and washed twice with ChIP Buffer. After removing all the liquid, Dynabeads were resuspended in 400 µl ChIP Buffer. 10 µg of antibody were added, and tubes were incubated at 4 °C for 2 h with rotation. After the incubation, antibody-bound beads were washed twice with ChIP Buffer.

For chromatin immunoprecipitation, chromatin extracts were added to the antibody-bound beads and incubated at 4 °C overnight with rotation and then washed with Nexus washing buffers A to D (wash buffer A: 10 mM Tris, 1 mM EDTA, 0.1% Triton X-100; wash buffer B: 150 mM NaCl, 20 mM Tris-HCl, pH 8.0, 5 mM EDTA, 5.2% sucrose, 1.0% Triton X-100, 0.2% SDS; wash buffer C: 250 mM NaCl, 5 mM Tris-HCl, pH 8.0, 25 mM HEPES, 0.5% Triton X-100, 0.05% sodium deoxycholate, 0.5 mM EDTA; wash buffer D: 250 mM LiCl, 0.5% IGEPAL CA-630, 10 mM Tris-HCl, pH 8.0, 0.5% sodium deoxycholate, 10 mM EDTA). End repair and dA-tailing were performed using the NEBNext End Repair Module and the NEBNext dA-Tailing Module. ChIP-nexus adaptors with mixed fixed barcodes (CTGA, TGAC, GACT, ACTG) were ligated with Quick T4 DNA ligase, and the products converted to blunt ends with Klenow fragment and T4 DNA polymerase. The samples were treated with lambda exonuclease and RecJf exonuclease. After each enzymatic reaction, the chromatin was washed with the Nexus washing buffers A to D and Tris buffer (10 mM Tris, pH 7.5, 8.0, or 9.5, depending on the next enzymatic step). The chromatin was then eluted and subjected to reverse crosslinking and ethanol precipitation. Purified single-stranded DNA was then circularized with CircLigase, annealed with oligonucleotides complementary to the BamHI restriction site, and linearized by BamHI digestion. The linearized single-stranded DNA was purified by ethanol precipitation and subjected to PCR amplification with NEBNext High-Fidelity 2X PCR Master Mix and ChIP-nexus primers. The ChIP-nexus libraries were then gel-purified before sequencing with Illumina NextSeq 500.

### ChIP-nexus data processing

ChIP-nexus data generated in this work and reprocessed from GSE85741^33^ were first preprocessed by trimming and reassigning fixed and random barcode sequence information, stored as read names for use in downstream processing. Reads were next trimmed to remove adapter sequences using Cutadapt (v.4.2)^110^ and aligned to the dm6 genome assembly using bowtie2 (v.2.3.5.1)^111^. Using aligned coordinates alongside the stored fixed and random barcode sequence information, reads were deduplicated. Coverage was generated by collecting only the first “stop” base of each aligned read. In order to compare reproducible, base-resolution footprinting profiles, each replicate ChIP-nexus experiment was first normalized via reads-per-million (RPM) scaling of coverage to the total aligned reads of a replicate experiment. Next, normalized replicate footprints were pooled and averaged by number of replicates per TF and condition.

### CAGE-seq data processing and *Drosophila* TSS annotation

Publicly available CAGE-seq data from *Drosophila* melanogaster Kc167 cells nuclear extracts (modENCODE: ENCSR827COJ) were processed using the STAR aligner (v.2.7.1a)^112^ against the *Drosophila* melanogaster UCSC dm6 reference genome. The resulting coordinate-sorted BAM files were analyzed using the CAGEr (v.2.14.0) package in R to obtain TSSs (Transcription Start Sites)^113^. Quality was assessed using Pearson correlations and biological replicates were merged and normalized using a power-law approach (*α* = 1.19*, T* = 10^6^) within the fit range of 3–40,000 counts.

Individual replicate TSSs were grouped into tag clusters within each sample using distance-based clustering (distclu, CAGEr package) with a maximum merging distance of 30 bp. To delineate precise coordinates for each tag cluster, the cumulative TSS signal was calculated and the 10th and 90th percentiles of the cumulative distribution were determined to delineate the central coordinates which contain 80% of transcription signal.

Tag clusters with a TPM ≥ 0.5 in at least one sample were then merged across replicates (aggregateTagClusters, CAGEr package) with a maximum inter-cluster distance of 100 bp to define consensus TSS clusters. The genomic coordinates of each consensus cluster were defined as the union of the constituent clusters’ central activity regions. Finally, cumulative TSS distributions and quantile positions were recomputed for the consensus clusters to refine their boundaries. The dominant TSS of each consensus cluster was identified as the genomic position with the highest summed TSS signal across the two replicate samples. For promoters that exhibited a single TSS, only those with ≥3 TPM were kept.

TSSs from FlyBase (r6.64, FB2025_03) were used to annotate the TSS clusters. If multiple clusters mapped to the same gene or transcript, only the cluster with the highest TPM was selected. In total, we identified 6314 active promoters. Active focused promoters where transcription initiation occurred within a narrow window (*n* = 1769) used for subsequent analysis were selected based on their interquantile cluster width (≤ 11 bp).

### RNA-seq library preparation

Total mRNA was extracted from *Drosophila* melanogaster Kc167 cells using Invitrogen TRIzol reagent. cDNA libraries were prepared using Illumina TruSeq Stranded mRNA Preparation Kit with polyA enrichment and sequenced on Illumina NextSeq 500.

### RNA-seq data processing

All RNA-seq paired-end sequencing data were aligned to the UCSC dm6 reference genome using STAR (v.2.7.1a)^112^ and raw read counts per gene were quantified with “--quantMode GeneCounts” option. RNA-seq levels are TPM-normalized across replicates, measured using the UCSC dm6 transcriptome annotations^114^.

### H3K4me3 ChIP-seq data processing

Publicly available H3K4me3 ChIP-seq data generated on *Drosophila* melanogaster Kc167 cells was downloaded from GEO accession GSE62904 and processed using bowtie^115^ against the *Drosophila* melanogaster UCSC dm6 reference genome. H3K4me3 ChIP-seq reads were compared against the corresponding input control using the SES (Signal Extraction Scaling)^116^ normalization method available in deepTools^117^ (v.3.4.2) to account for differences in sequencing depth and background signal. Input-normalized signal was calculated by subtracting the scaled input coverage from the H3K4me3 coverage “--operation subtract” in 50-bp bins across the genome. The resulting normalized signal was exported as a bigWig file for downstream analysis.

### Principal component analysis

Average ChIP-nexus binding profiles under DMSO control and triptolide treatment were quantified per base pair within a 151 bp window centered on each annotated TSS. The average signal for each ChIP-nexus signal (*x*) was normalized via $x-\,\min (x)\,/\,\max (x)\,-\min (x)$ and subjected to principal component analysis (PCA) using the function prcomp (center = TRUE, scale = FALSE) from the base stats package in R. Modules were defined based on PCA clustering and similarity in the average binding profiles.

### Promoter type characterization

Promoter types were selected based on the presence or absence of the motifs listed in Supplementary Table 1. Each genomic sequence had to match the consensus sequence, with up to one mismatch for the TATA box and zero mismatches for the other elements. This sequence matching occurred at each promoter within the coordinate boundaries reported in Supplementary Table 1. TATA and TCT promoters were only required to contain the TATA box or the TCT, respectively, and were not excluded for having other elements. DPR promoters were allowed the presence of MTE, DPE, and PB, but not TATA, TCT, Ohler 1/6/7, or DRE, whereas housekeeping promoters were allowed to contain Ohler 1/6/7 or DRE, but not TATA, MTE, DPE, PB or TCT.

### TBP profile clustering

To cluster promoters based on the TBP ChIP-nexus profile we selected the top 90% narrow promoters given the total TBP ChIP-nexus signal across an 81 bp window centered at the TSS. To emphasize binding pattern shape, signals for each promoter and position relative to the TSS were ranked from the smallest to the largest values and divided by the number of columns, so the transformed values went from 0 to 1. We then performed K-means clustering (centers = 4, nstart = 100 and *x* = 30) using the base stats package in R.

### Promoter element enrichment

Known *Drosophila* promoter elements in each promoter were identified by the presence of the known consensus sequence with zero mismatches in a specified window relative to the TSS (Supplementary Table 1). For each k-means cluster and each promoter element, the enrichment in Fig. 3E was calculated by determining the ratio between the fraction of promoter elements in the cluster and the fraction of the same promoter element in the other three clusters. The significance for the observed frequencies was calculated using two-sided Fisher’s exact test applied to 2 × 2 contingency tables comparing the numbers of promoters with and without each motif in the cluster of interest versus all other clusters and corrected for multiple testing with the Benjamini–Hochberg method using stats::fisher.test() in R. Exact statistical values are reported in Table S2.

### TAF/TBP occupancy ratios at TATA and DPR promoters

Relative ChIP-nexus occupancy was calculated as the log2-transformed ratio between the total signal of each factor and the total TBP signal, measured within a 100-bp window centered on the TSS. For each factor, relative occupancy ratios were median-centered by subtracting the median log2(factor/TBP) value across promoters. Extreme outliers were excluded from the plot for visualization purposes.

### Normalization of the ChIP-nexus binding by CAGE-seq expression

To visualize average binding effects around active TSSs, the ChIP-nexus signal was scaled relative to the measured CAGE-seq expression score across the corresponding promoter (−150 bp to +150 bp). Next, ChIP-nexus signal for each factor at each promoter was quantified using windows defined by the predominant binding footprint of each factor relative to the TSS and scaled to the promoter’s CAGE-seq expression levels in order to compare signals between promoter types. TFIIA, TFIIB, TBP, TAF10, TAF4, TAF12, TAF9, TAF5 and TAF13 (upstream broad and narrow modules) were measured within 50 bp upstream of the TSS; TAF6, TAF8 and TAF11 (middle module) were measured within 100 bp centered on the TSS; and TAF1, TAF2, TAF7 and TAF3 (downstream module) were measured within 50 bp downstream of the TSS. Statistical significance was assessed using two-sided Wilcoxon rank-sum tests with Benjamini–Hochberg correction (Fig. 4B). Extreme outliers were excluded from the plot for visualization purposes. Effect sizes for two-sided Wilcoxon rank-sum tests were reported as the rank-biserial correlation (r), calculated from the Mann–Whitney U statistic: r=2U/(n1n2)−1, where *n*_1_ and *n*_2_ are the sample sizes of the two groups. Two-sided 95% confidence intervals were estimated by bootstrap resampling. Exact statistical values are reported in Table S3. 

### Hierarchical clustering of TFIID and PIC binding

To compare similarity of ChIP-nexus binding of TFIID subunits and PIC components, binding levels were measured around annotated TSSs (−50 bp to +50 bp) and pairwise Pearson correlations (log2 + 1). Correlations were next transformed to distance values (1−r) and hierarchically clustered using the Ward’s D linkage method. Hierarchical clustering of binding correlations was performed across all narrow promoters (Fig. 1D), as well as for each promoter type separately (Fig. 4D).

### Paused Pol II half-life measurement

Paused Pol II half-life was re-measured at CTSS promoters with narrow transcription initiation windows from Kc167 cells^33^ with an updated genome (UCSC dm6). Briefly, to analyze the half-life of paused Pol II from these data, promoters were selected if (1) the total Pol II signal was high (average ChIP-nexus signal >20), (2) a typical footprint was observed for Pol II (distance between positive- and negative-strand maximum <80 bp) and (3) the position of the Pol II footprint was less than 80 bp downstream of the TSS. Upon re-processing, 1307 promoters fulfilled these criteria. For each promoter, the Pol II ChIP-nexus signal was calculated in a 51 bp window centered on the pausing position (the midpoint between Pol II–positive and Pol II–negative maximum). To calculate the half-life of paused Pol II at each promoter, previously generated Pol II time-course measurements^33^ were fitted into an exponential decay model using nonlinear regression. Promoters with a paused Pol II half-life of longer than 60 min were capped at 60 min to eliminate inflated values due to noise. The 1307 promoters were then ranked based on half-life and divided into five quintiles. Promoters within quintile 1 (*n* = 262; Pol II pausing half-lives <6 min) were selected as promoters with short Pol II pausing and promoters within quintile 5 (*n* = 262; Pol II pausing half-lives > 45 min) were selected as promoters with long or stable Pol II pausing.

### Antibodies

Custom rabbit polyclonal antibodies against TFIID subunits were generated by GenScript for the Zeitlinger laboratory. Additional custom rabbit polyclonal antibodies against TBP, NC2α, NC2β, Mot1 and TRF2 were provided by the James T. Kadonaga laboratory. Antibodies generated in this study are available from the corresponding author upon request. Antibodies were not independently validated by Western blotting. Instead, their specificity was infered from the characteristic and reproducible ChIP-nexus binding footprints observed at promoter regions. See Supplementary Table 8 for details on epitope design.

### Reporting summary

Further information on research design is available in the Nature Portfolio Reporting Summary linked to this article.

## Supplementary information

Supplementary Information

Reporting Summary

Transparent Peer Review file

## Data Availability

The raw RNA-seq and ChIP-nexus data generated in this study are available under GEO accession number GSE314461. Gene expression table counts and ChIP-nexus tracks are readily available under the same GEO accession. CAGE-seq data were downloaded from modENCODE, accession number ENCSR827COJ. Previously published TBP, Pol II and Nelf ChIP-nexus are available under GEO accession GSE85741 and H3K4me3 ChIP-seq is available under GEO accession GSE62904. All protein structures, the DNA-bound human TFIID [https://www.rcsb.org/structure/6MZM], yeast TFIID [https://www.rcsb.org/structure/6HQA], TFIIA-TBP [https://www.rcsb.org/structure/1NVP], TAF1-7-2 [https://www.rcsb.org/structure/6MZM], TAF4-12 [https://www.rcsb.org/structure/1H3O], TAF6-9 [https://www.rcsb.org/structure/6MZD], TAF11-13 [https://www.rcsb.org/structure/1BH8] and NC2-TBP-DNA [https://www.rcsb.org/structure/1JFI], were downloaded from the RCSB Protein Data Bank and colored appropriately using UCSF CHIMERA^118^. The source data used to generate all figures is available at [https://doi.org/10.5281/zenodo.21753147].

## Code availability

The analysis code is available on GitHub at [https://github.com/zeitlingerlab/G-M_Alcantara_TFIID_2025].
