# Supplementary material for: High-resolution binding data of TFIID and cofactors show promoter-specific differences
Source: Nat Commun. 2026 Oct 7;17:11446. doi: 10.1038/s41467-026-77758-9 (PMC13736863; doi:10.1038/s41467-026-77758-9)
Supplement: Supplementary file 1 [file 41467_2026_77758_MOESM1_ESM.pdf]

Supplementary figures

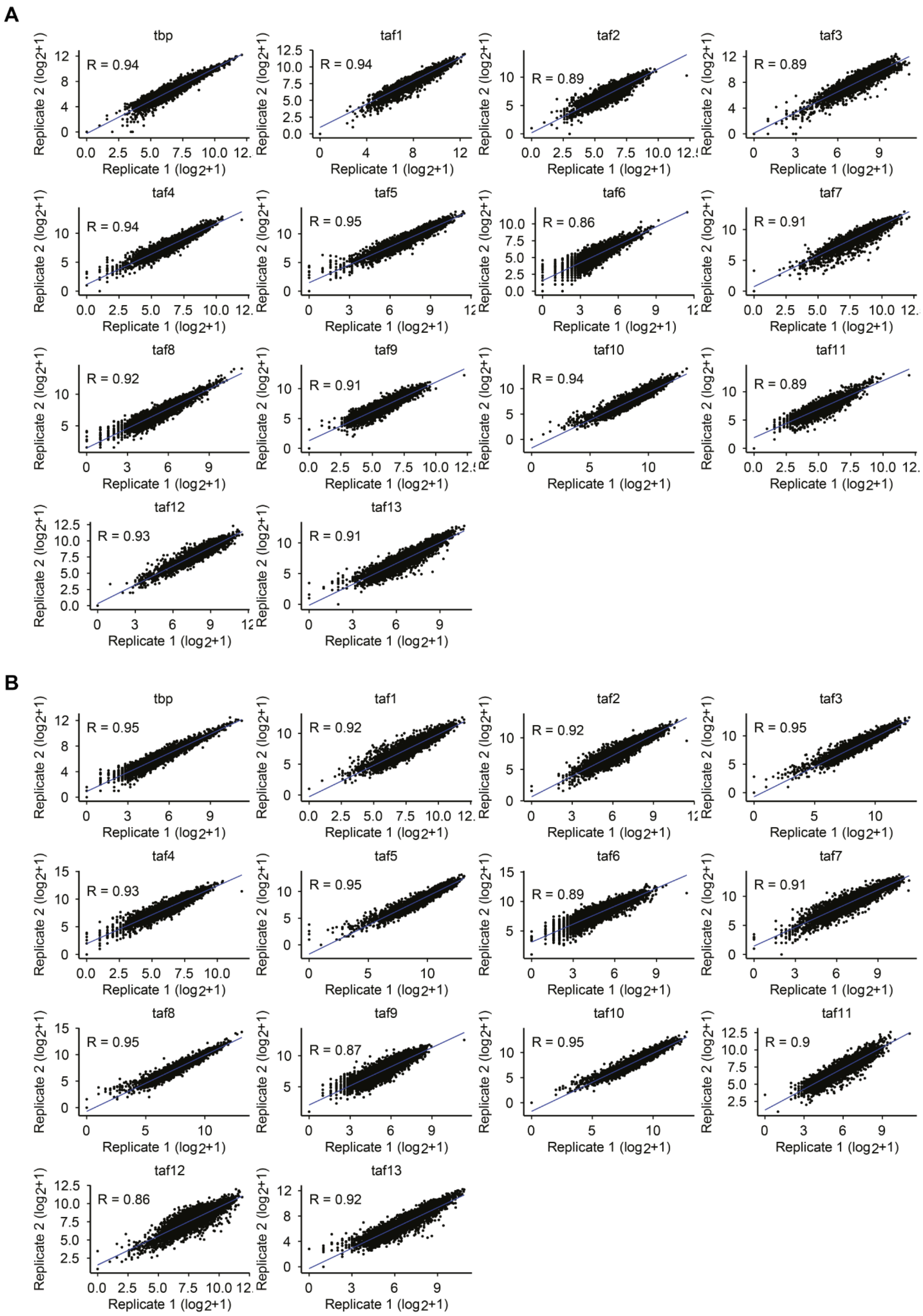

Figure S1: ChIP-nexus TFIID replicates show high reproducibility. Pearson correlations of ChIP-nexus read counts between replicate experiments for each TFIID subunit under DMSO control conditions (A) and after triptolide treatment (B). Each dot represents the total read count calculated within 100 bp centered on the TSS of each narrow promoter.

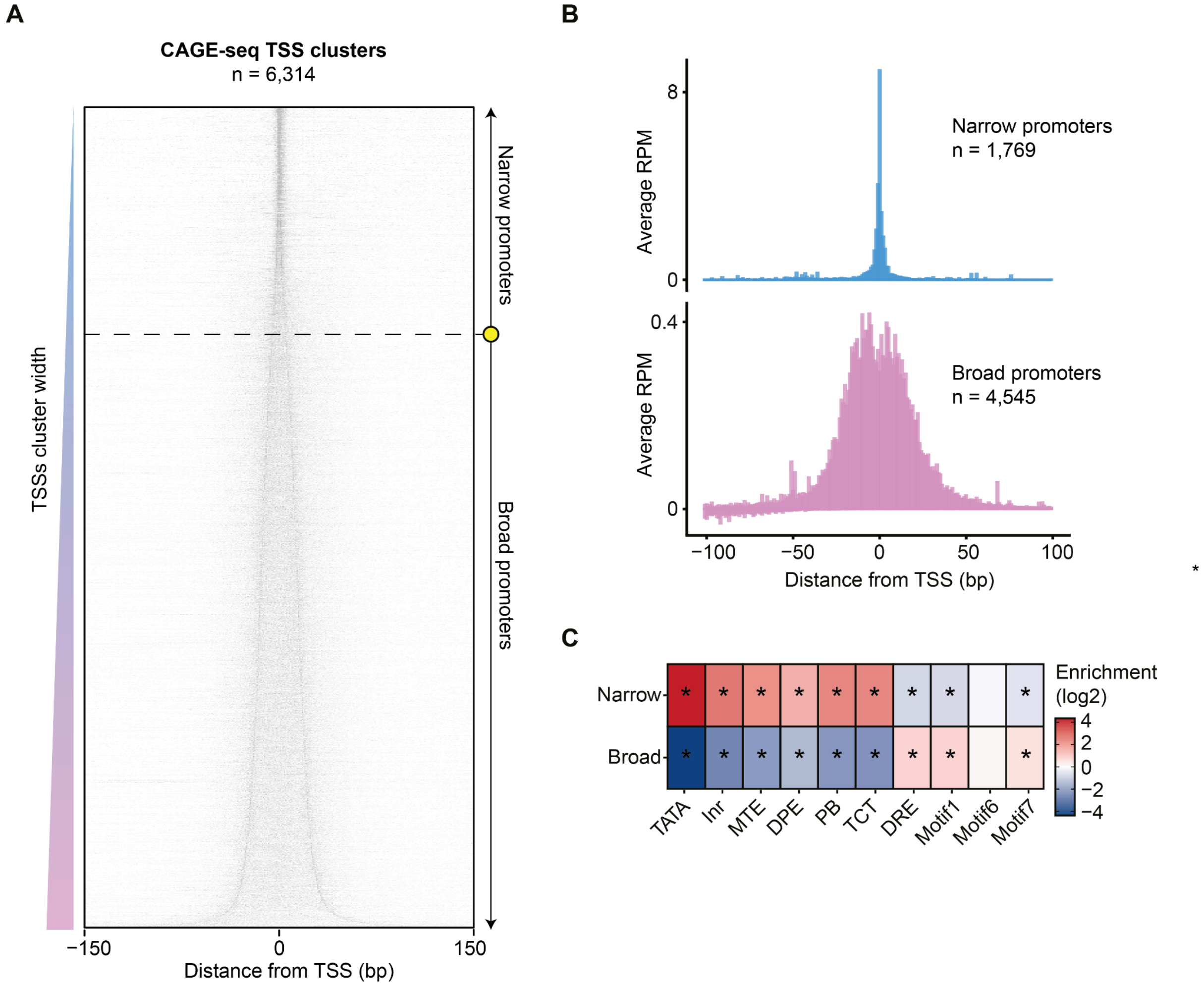

Figure S2: Identification of promoters with narrow *versus* broad transcription initiation by CAGE-seq. A) Heatmap showing the normalized CAGE-seq signal at all selected active promoters (n = 6,314). TSSs are centered at the midpoint and promoters are sorted by TSS cluster width (see Methods). The dotted line separates narrow promoters (cluster width ≤ 11 bp) from broad promoters (cluster width > 11 bp). **B)** Average CAGE-seq signal at narrow and broad promoters. **C)** Enrichment of core promoter elements in narrow and broad promoters. Statistical significance was calculated using Fisher's exact test with Benjamini–Hochberg correction: $p < 0.05$. Full statistical details are reported in Supplementary Table 4.

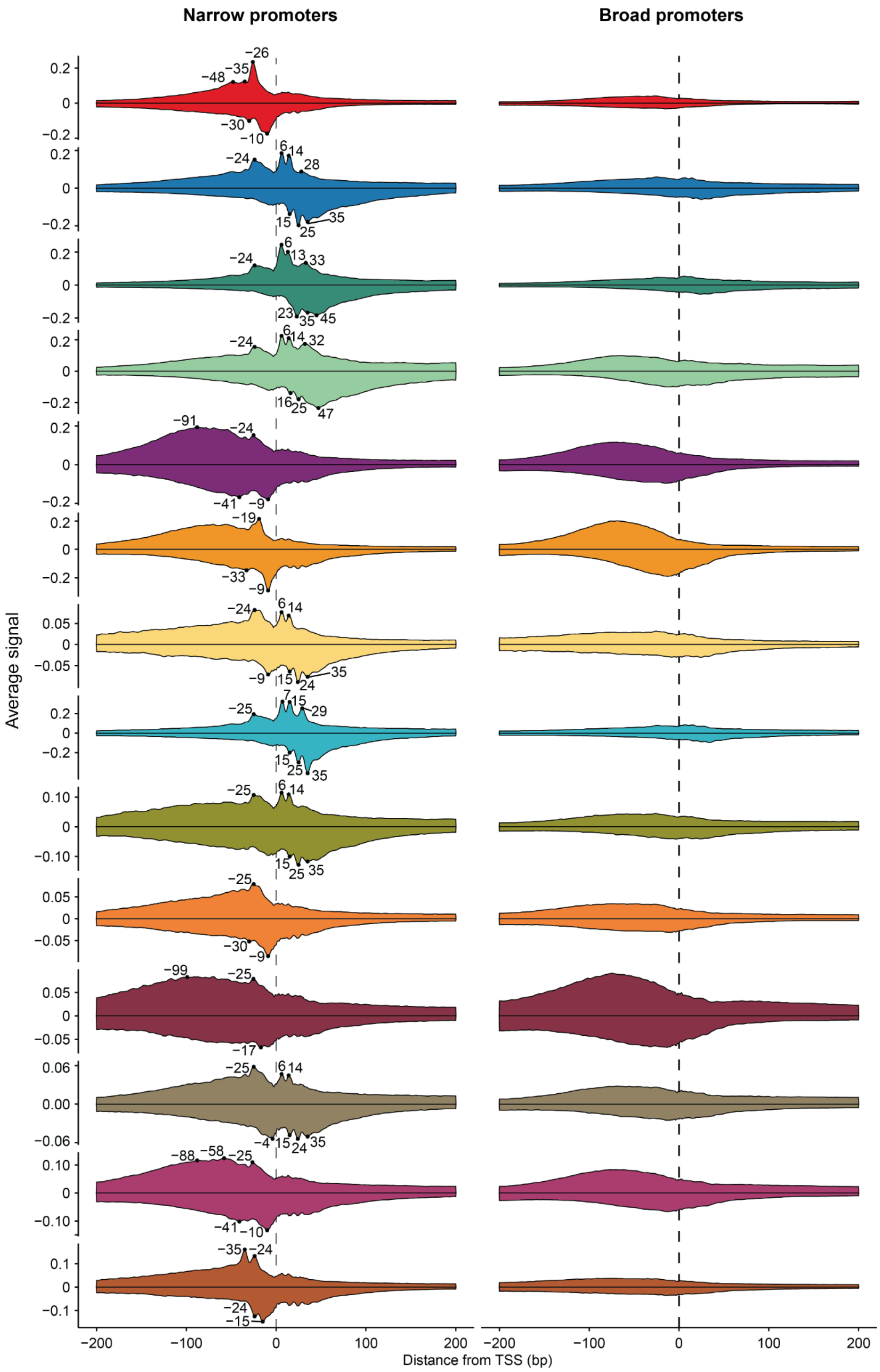

Figure S3: TFIID binding profiles are more diffuse at promoters with broad transcription initiation. Average ChIP-nexus binding profiles of TFIID subunits after triptolide treatment at narrow (left) and broad (right) promoters. Numbers at narrow promoters indicate the position (bp) of the average profile summits on each strand used to calculate the midpoint binding regions reported in Figure 2. Each profile is centered at the TSS and shown with the binding signal at the positive strand and negative strand in the same color.

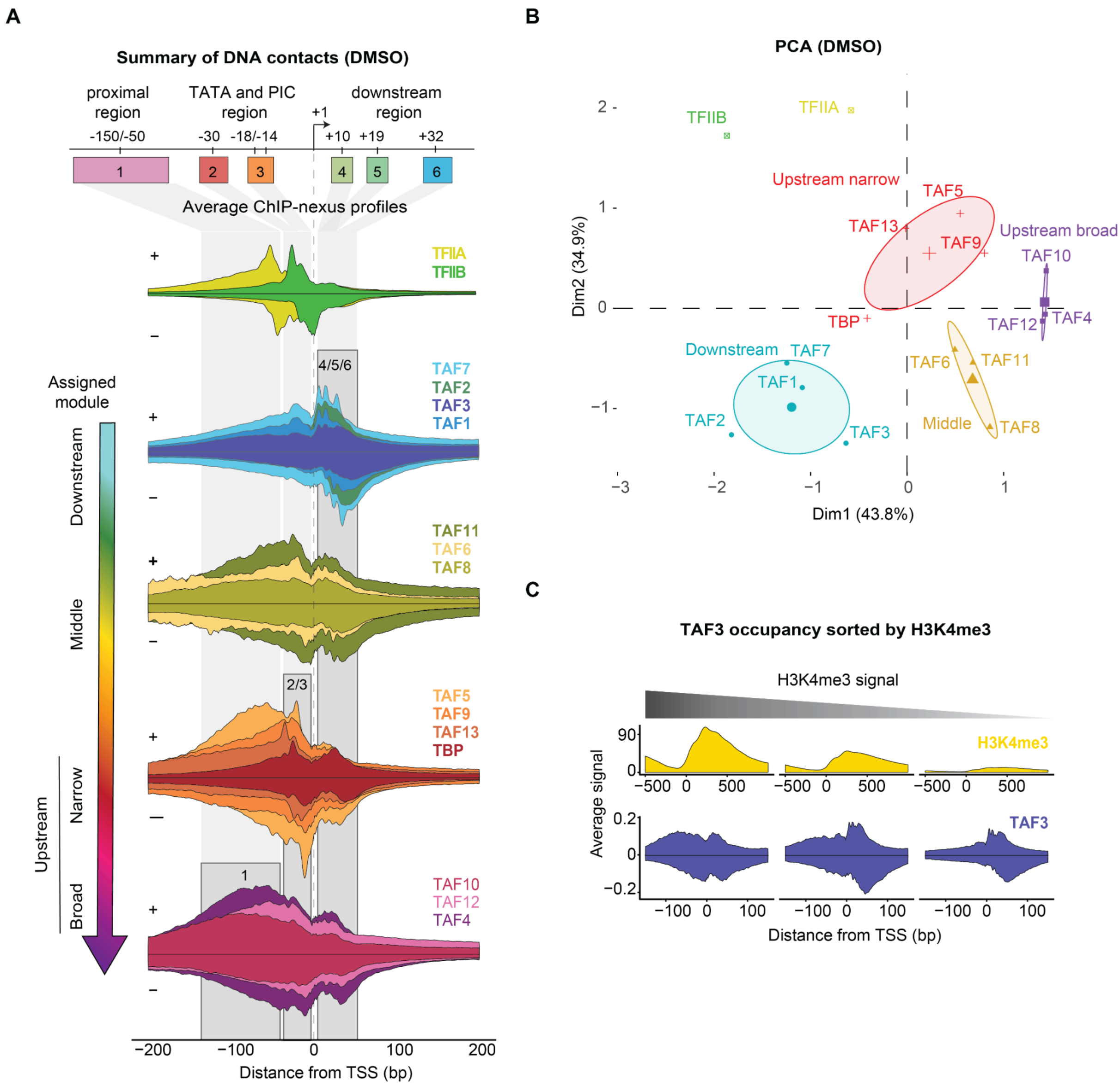

Figure S4: The overall modular binding organization of TFIID is preserved under DMSO control condition. A) Average ChIP-nexus binding profiles of TFIIA, TFIIB and TFIID subunits centered at the TSS. For each subunit, the positive- and negative-strand binding profiles are shown in the same color and scaled arbitrarily to facilitate comparison across subunits. **B)** Principal component analysis (PCA) of the averaged ChIP-nexus profiles shown in panel A. **C)** TAF3 binding profiles sorted by decreasing H3K4me3 levels, showing no association between the downstream TAF3 footprint and H3K4me3 enrichment.

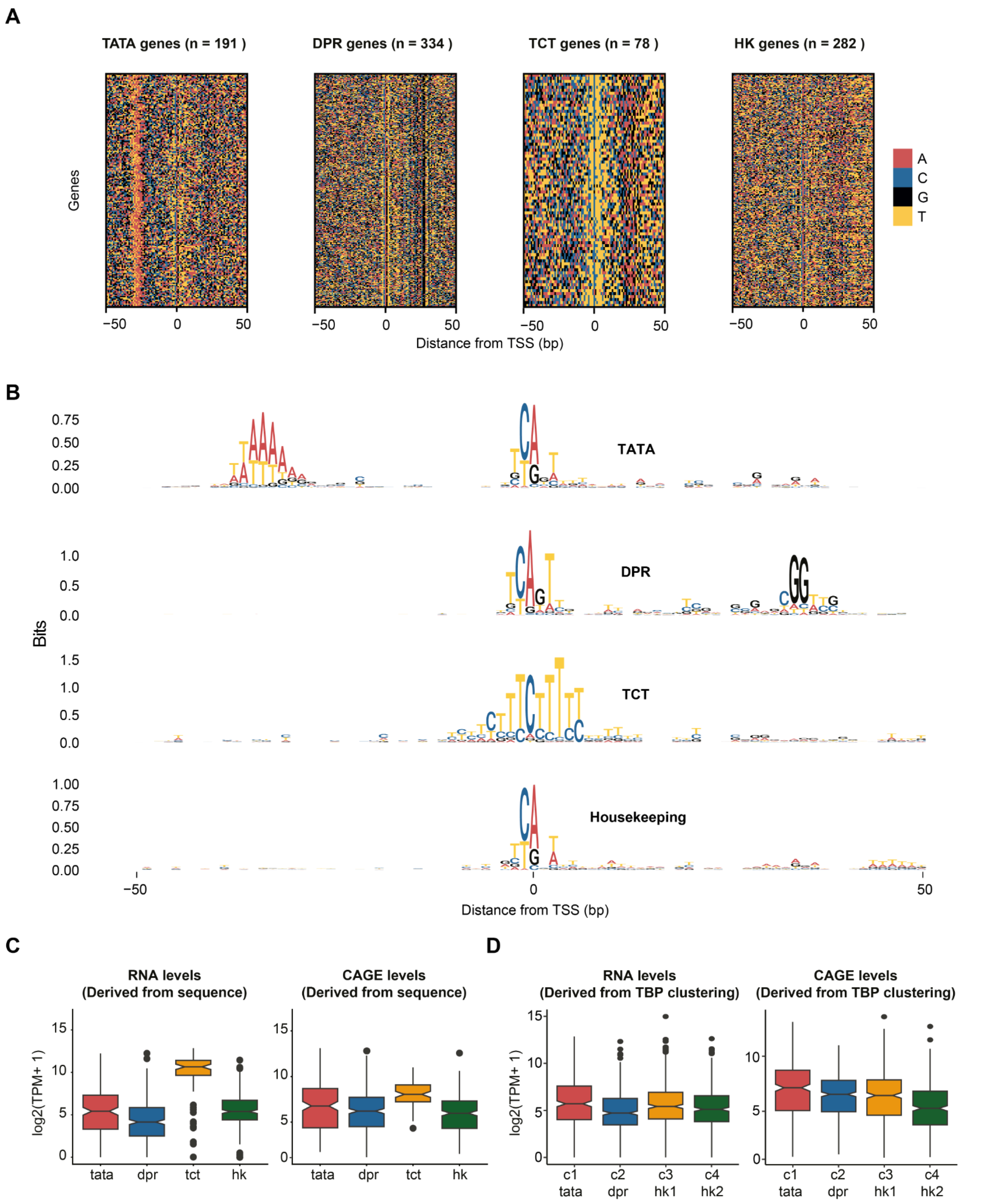

Figure S5: Distinct sequence composition and expression profiles define TATA, DPR, TCT and housekeeping promoters. A) DNA sequence heatmaps showing differences in DNA composition among TATA, DPR, TCT and HK core promoters selected based on the motif sequences from Supplementary Table 1 (see Methods). **B)** Position weight matrices (PWMs) of the promoter classes shown in panel A. **C)** RNA-seq and CAGE-seq mRNA levels for the promoter classes defined in panel A. **D)** RNA-seq and CAGE-seq mRNA levels for the clusters derived from the TBP profiles shown in Figure 3. For panel C and D, the central line indicates the median, the box spans the 25th–75th percentiles, and whiskers extend to the most extreme values within 1.5 × the interquartile range. Notches indicate the approximate 95% confidence interval around the median.

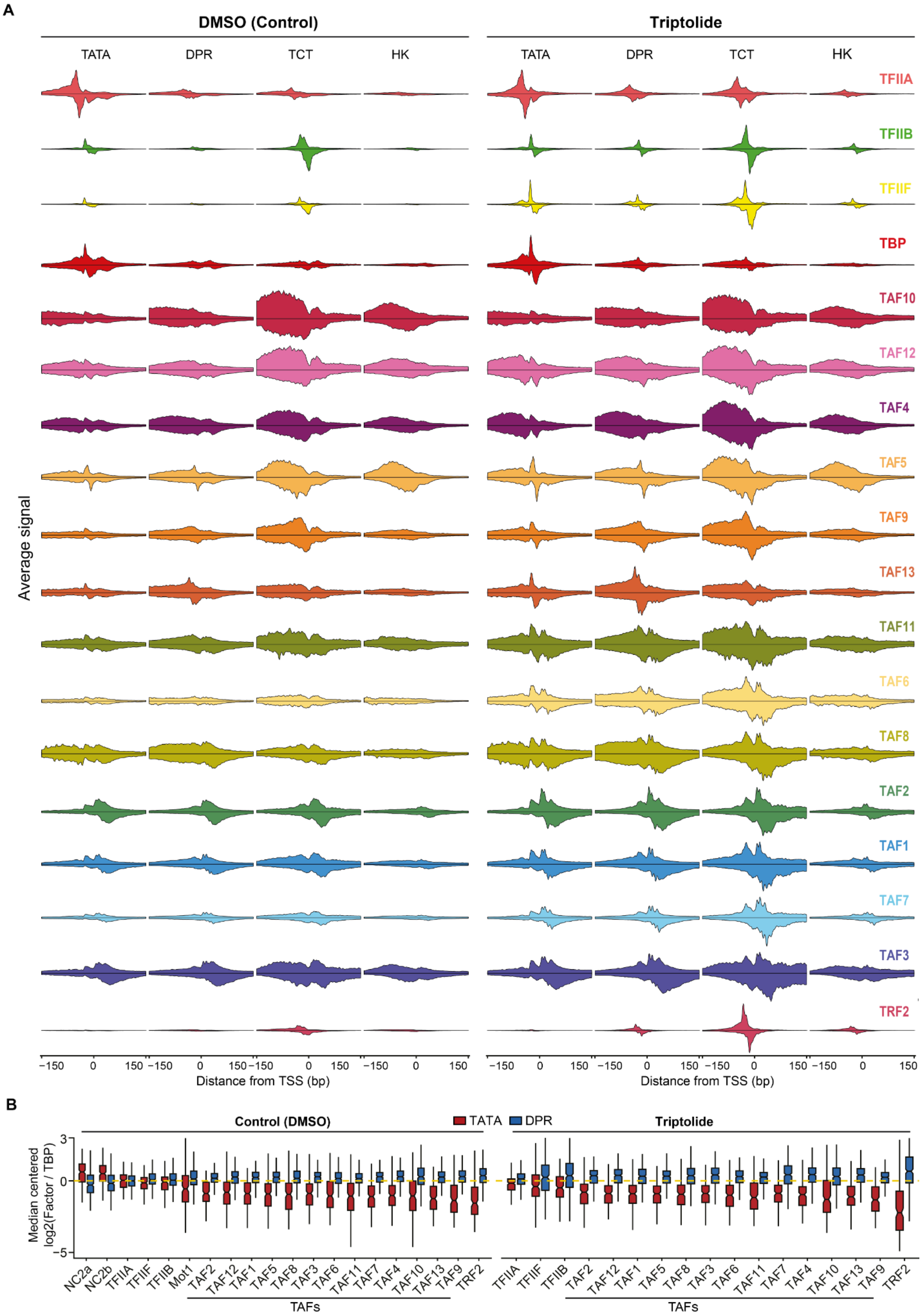

Figure S6: TAFs show similar binding patterns across promoter types but reduced occupancy relative to TBP at TATA promoters. A) Average ChIP-nexus binding profiles of TFIIA, TFIIB, TFIIF, TRF2 and TFIID subunits at TATA (n = 191), DPR (n = 334), TCT (n = 78) and HK (n = 282) promoters, centered at the TSS, under DMSO control and triptolide conditions. B) Occupancy distribution of each factor relative to TBP at TATA and DPR promoters. Relative ChIP-nexus occupancy was calculated as the log2-transformed ratio between the total signal of each factor and the total TBP signal, measured within a 100-bp window centered on the TSS. For each factor, relative occupancy ratios were median-centered by subtracting the median log2(factor/TBP) value across promoters. The central line indicates the median, the box spans the 25th–75th percentiles, and whiskers extend to the most extreme values within 1.5 × the interquartile range. Notches indicate the approximate 95% confidence interval around the median. Extreme outliers were excluded from the plot for visualization.

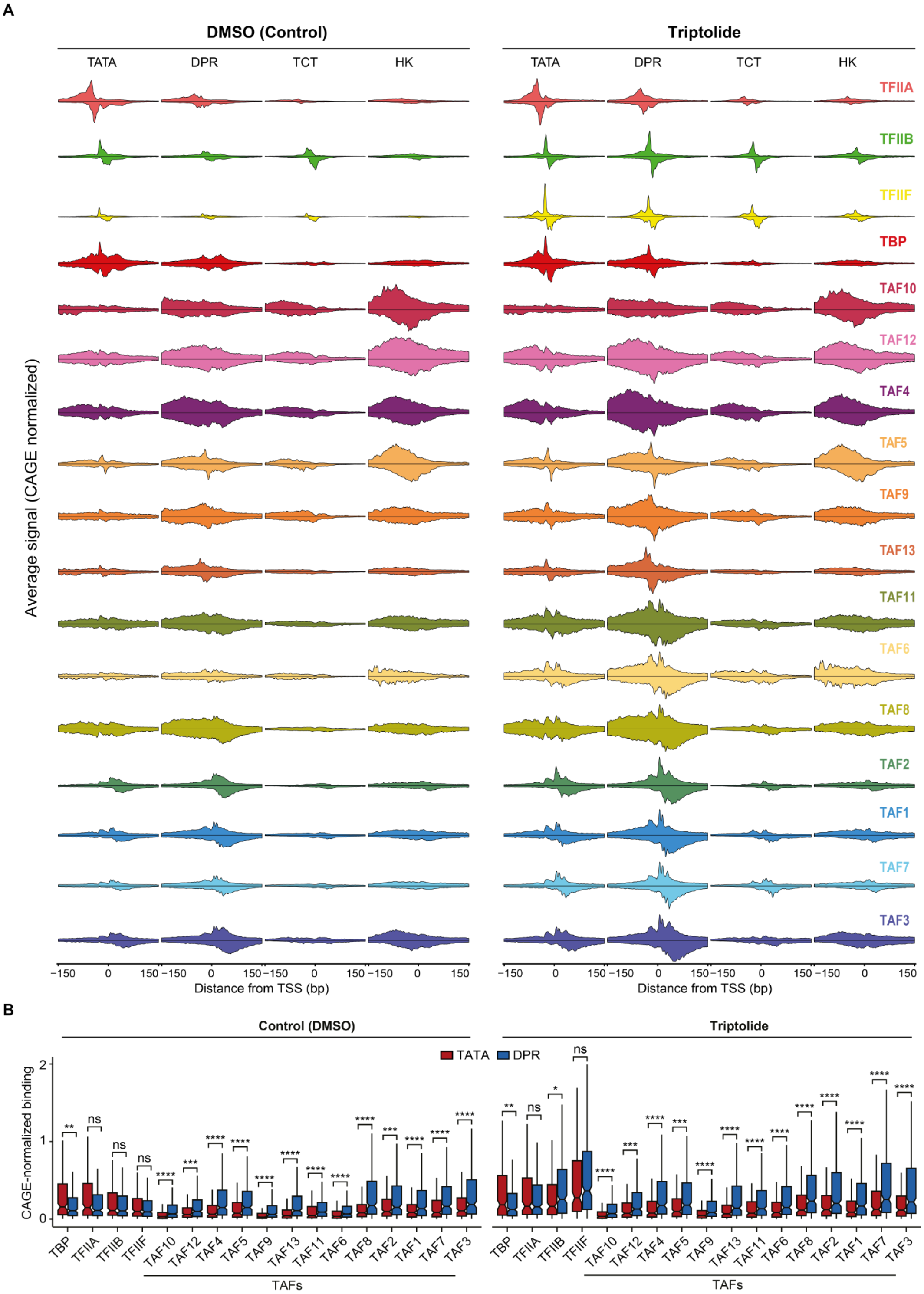

Figure S7: Transcription-normalized occupancy reveals reduced TAF binding at TATA promoters. A) CAGE-seq normalized average ChIP-nexus binding profiles of TFIIA, TFIIB, TFIIF and TFIID subunits at TATA (n = 191), DPR (334), TCT (n = 78) and HK (n = 282) promoters, centered at the TSS, under DMSO (control) and triptolide conditions. **B)** CAGE-seq normalized signal distribution for the factors shown in panel A (Wilcoxon test). Normalized occupancy at each promoter was calculated by dividing the total ChIP-nexus signal (measured using factor-specific windows around the TSS; see methods) by the corresponding CAGE-seq levels. Statistical significance was performed using two-sided Wilcoxon rank-sum tests with Benjamini–Hochberg correction. Stars represent adjusted p values: ns (no significant), $p \geq 0.05$; *, $p < 0.05$; **, $p < 0.01$; ***, $p < 0.001$; ****, $p < 0.0001$. Exact p values, effect sizes and 95% confidence intervals are reported in Supplementary Table 3. The central line indicates the median, the box spans the 25th–75th percentiles, and whiskers extend to the most extreme values within 1.5 × the interquartile range. Notches indicate the approximate 95% confidence interval around the median. Values beyond the whiskers were not displayed but were retained for the statistical tests.

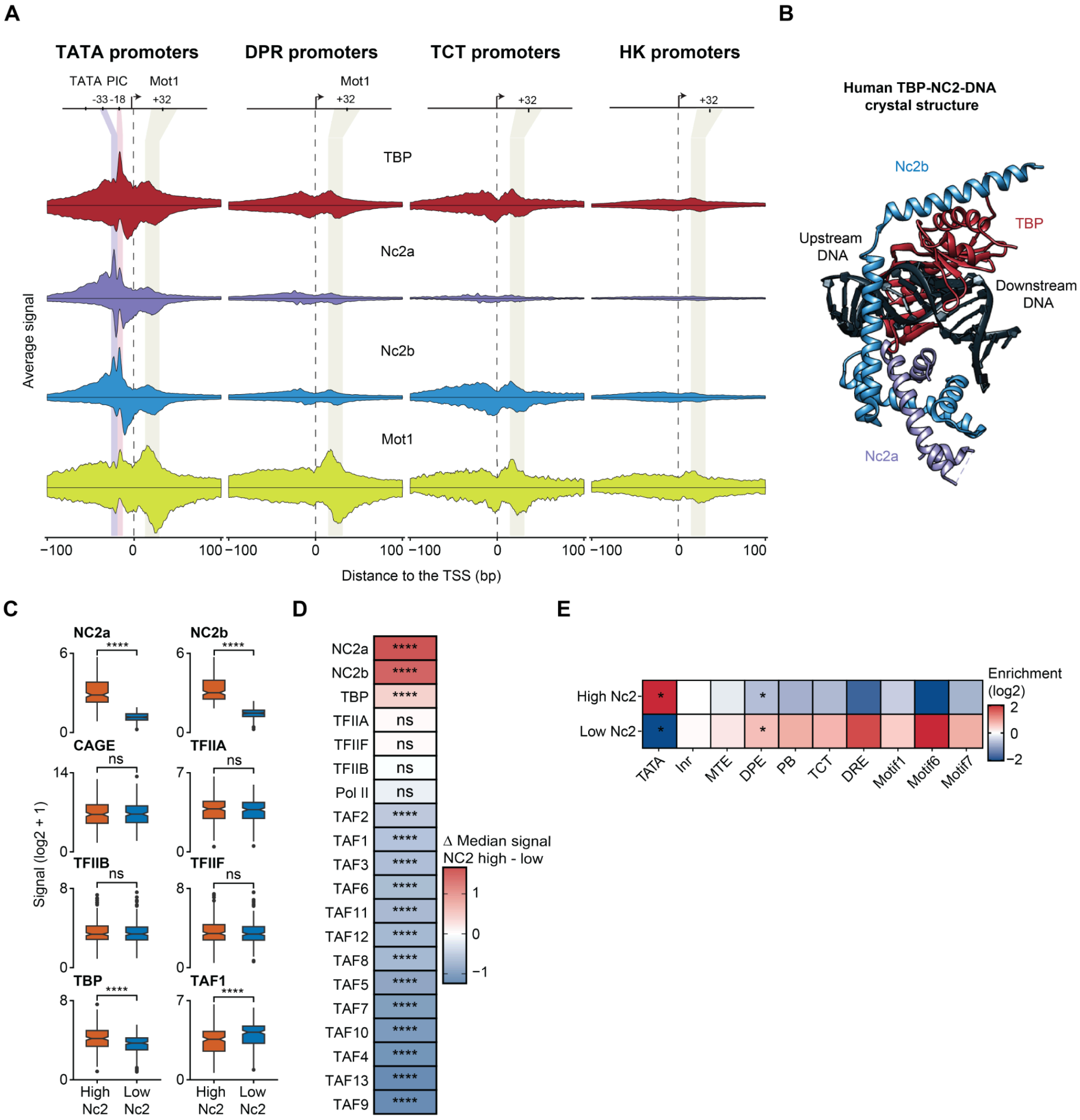

Figure S8: NC2 occupancy is associated with reduced TAF binding. A) Average ChIP-nexus binding profiles of NC2α, NC2β and Mot1 at TATA (n = 191), DPR (n = 334), TCT (n = 78), and HK (n = 282) promoters centered at the TSS. **B)** Crystal structure of human NC2–TBP–DNA (PDB: pdb_00001jfi). **C)** CAGE-seq levels and total ChIP-nexus binding distributions for NC2α, NC2β, TBP, TFIIA, TFIIB, TFIIF and TAF1 at NC2-high (n = 203) and NC2-low promoters (n = 203). The central line indicates the median, the box spans the 25th–75th percentiles, and whiskers extend to the most extreme values within 1.5 × the interquartile range. Notches indicate the approximate 95% confidence interval around the median. **D)** Median differences in ChIP-nexus signal for NC2α, NC2β, TBP, TFIIA, TFIIB, TFIIF, Pol II, and TAFs between NC2-high and NC2-low promoters. For each factor, the difference was calculated by subtracting the median total signal at NC2-high promoters from the median total signal at NC2-low promoters. Red indicates higher occupancy at NC2-high promoters, whereas blue indicates higher occupancy at NC2-low promoters. ChIP-nexus signal was measured within 100 bp centered on the TSS. For panels C and D, statistical significance was performed using two-sided Wilcoxon rank-sum tests with Benjamini–Hochberg correction: ns (no significant); ****, $p < 0.0001$. **E)** Enrichment of core promoter elements at NC2-high and NC2-low promoters. Statistical significance was calculated using a two-sided Fisher's exact test with multiple-testing correction: *$p < 0.05$. Infinite enrichment values resulting from zero motif occurrences in the comparison group were capped at the maximum finite enrichment for visualization. Full statistical details are reported in Supplementary Tables 5 and 6.

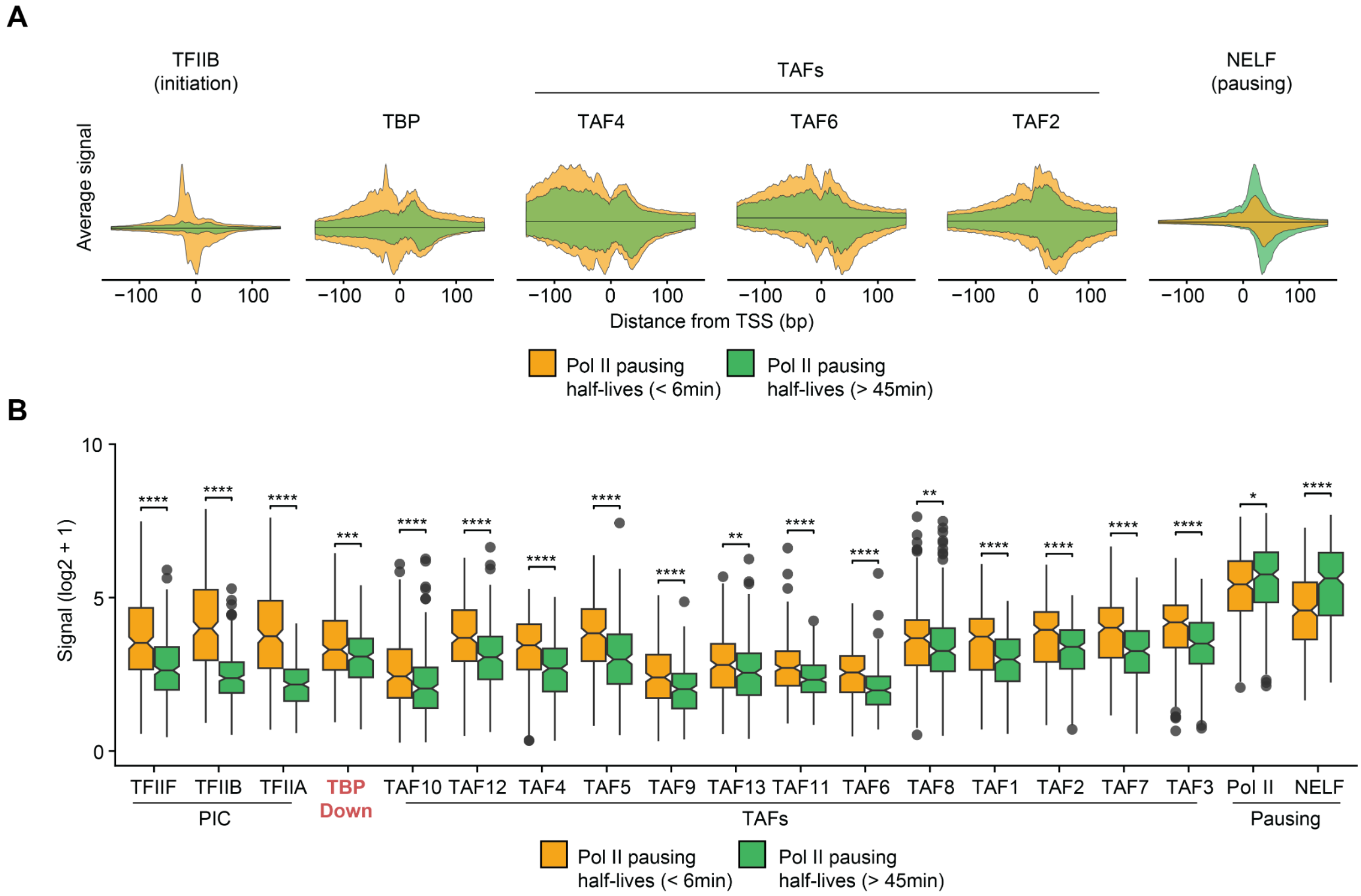

Figure S9: Downstream TBP and TAF levels are reduced at promoters with stable Pol II pausing. A) Average ChIP-nexus binding profiles of TFIIB, TBP, TAF4, TAF6, TAF2 and NELF around the TSS at promoters with short Pol II pausing half-lives (< 6 min; yellow; n = 262) and long Pol II pausing half-lives (> 45 min; green; n = 262). **B)** Total ChIP-nexus levels at promoters with short and long Pol II pausing half-lives. TBP, PIC, and TAF levels are lower at stably paused promoters, whereas Pol II and NELF levels are increased. ChIP-nexus signal was calculated within 100 bp centered at the TSS for PIC components and TAFs and 50 bp downstream of the TSS for TBP, Pol II and NELF. The central line indicates the median, the box spans the 25th–75th percentiles, and whiskers extend to the most extreme values within 1.5 × the interquartile range. Notches indicate the approximate 95% confidence interval around the median. Statistical significance was performed using two-sided Wilcoxon rank-sum tests with Benjamini–Hochberg correction: $p \geq 0.05$; *, $p < 0.05$; **, $p < 0.01$; ***, $p < 0.001$; ****, $p < 0.0001$. Exact p values, effect sizes and 95% confidence intervals are reported in Supplementary Table 7.

Supplementary Tables

Name	Sequence	Start window	End window
TATA	STATAWAWR	-40	-20
INR	TCAKTY	-5	5
DPE	GGWYV	25	35
MTE	CSARCSSA	10	30
PB	KCGRWCG	20	50
DRE	WATCGATW	-70	-10
Ohler1	GGTCACACTR	-10	10
Ohler6	RGTATWTT	-50	-10
Ohler7	CAKCNCTR	-70	10
TCT	YYCTTTYY	-10	10

Table 1: Summary of features used for promoter elements sequence motifs selection.

Cluster	Motif	Cluster n/N (%)	Other clusters n/N (%)	Fold enrichment (log2)	Adjusted p-value (Fisher)	Significance	Direction
1	DPE	71/309 (23.0%)	252/1283 (19.6%)	0.226	0.276373	ns	enrichment
1	DRE	11/309 (3.6%)	136/1283 (10.6%)	-1.57	0.000128765	***	depletion
1	Inr	75/309 (24.3%)	297/1283 (23.1%)	0.0683	0.745407	ns	enrichment
1	MTE	8/309 (2.6%)	30/1283 (2.3%)	0.147	0.856646	ns	enrichment
1	Motif1	7/309 (2.3%)	23/1283 (1.8%)	0.338	0.731306	ns	enrichment
1	Motif6	14/309 (4.5%)	50/1283 (3.9%)	0.217	0.731306	ns	enrichment
1	Motif7	23/309 (7.4%)	142/1283 (11.1%)	-0.572	0.0913889	ns	depletion
1	PB	32/309 (10.4%)	99/1283 (7.7%)	0.424	0.191867	ns	enrichment
1	TATA	53/309 (17.2%)	21/1283 (1.6%)	3.39	5.42866e-22	****	enrichment
1	TCT	15/309 (4.9%)	62/1283 (4.8%)	0.00654	1	ns	enrichment
2	DPE	149/509 (29.3%)	174/1083 (16.1%)	0.866	1.78793e-08	****	enrichment
2	DRE	27/509 (5.3%)	120/1083 (11.1%)	-1.06	0.000376044	***	depletion
2	Inr	178/509 (35.0%)	194/1083 (17.9%)	0.965	5.45022e-12	****	enrichment
2	MTE	15/509 (2.9%)	23/1083 (2.1%)	0.473	0.47299	ns	enrichment
2	Motif1	4/509 (0.8%)	26/1083 (2.4%)	-1.61	0.0459999	*	depletion
2	Motif6	16/509 (3.1%)	48/1083 (4.4%)	-0.496	0.353103	ns	depletion
2	Motif7	33/509 (6.5%)	132/1083 (12.2%)	-0.911	0.000991699	***	depletion
2	PB	65/509 (12.8%)	66/1083 (6.1%)	1.07	4.54355e-05	****	enrichment
2	TATA	6/509 (1.2%)	68/1083 (6.3%)	-2.41	5.31829e-06	****	depletion
2	TCT	8/509 (1.6%)	69/1083 (6.4%)	-2.02	3.00708e-05	****	depletion
3	DPE	64/392 (16.3%)	259/1200 (21.6%)	-0.403	0.0434196	*	depletion
3	DRE	67/392 (17.1%)	80/1200 (6.7%)	1.36	3.02078e-08	****	enrichment
3	Inr	65/392 (16.6%)	307/1200 (25.6%)	-0.626	0.000536772	***	depletion
3	MTE	8/392 (2.0%)	30/1200 (2.5%)	-0.293	0.745407	ns	depletion
3	Motif1	6/392 (1.5%)	24/1200 (2.0%)	-0.386	0.745407	ns	depletion
3	Motif6	6/392 (1.5%)	58/1200 (4.8%)	-1.66	0.00575602	**	depletion
3	Motif7	81/392 (20.7%)	84/1200 (7.0%)	1.56	8.40738e-12	****	enrichment
3	PB	23/392 (5.9%)	108/1200 (9.0%)	-0.617	0.0866032	ns	depletion
3	TATA	7/392 (1.8%)	67/1200 (5.6%)	-1.64	0.00285374	**	depletion
3	TCT	45/392 (11.5%)	32/1200 (2.7%)	2.11	8.59498e-10	****	enrichment
4	DPE	39/382 (10.2%)	284/1210 (23.5%)	-1.2	2.61353e-08	****	depletion
4	DRE	42/382 (11.0%)	105/1210 (8.7%)	0.341	0.25846	ns	enrichment
4	Inr	54/382 (14.1%)	318/1210 (26.3%)	-0.895	2.57688e-06	****	depletion
4	MTE	7/382 (1.8%)	31/1210 (2.6%)	-0.483	0.683431	ns	depletion
4	Motif1	13/382 (3.4%)	17/1210 (1.4%)	1.28	0.0312666	*	enrichment
4	Motif6	28/382 (7.3%)	36/1210 (3.0%)	1.3	0.00106748	**	enrichment
4	Motif7	28/382 (7.3%)	137/1210 (11.3%)	-0.627	0.0443132	*	depletion
4	PB	11/382 (2.9%)	120/1210 (9.9%)	-1.78	1.21956e-05	****	depletion
4	TATA	8/382 (2.1%)	66/1210 (5.5%)	-1.38	0.0100534	*	depletion
4	TCT	9/382 (2.4%)	68/1210 (5.6%)	-1.25	0.0168176	*	depletion

Table 2: Summary of Figure 3E statistics. n = the number of promoters in that group that contain the motif and N = the total number of promoters in that group.

Treatment	Factor	TATA median	DPR median	Rank-biserial r (95% CI)	Adjusted p-value (Wilcoxon)	Significance
DMSO	taf1	0.086	0.136	−0.22 (−0.32, −0.12)	3.72824E-05	****
DMSO	taf10	0.032	0.068	−0.30 (−0.39, −0.20)	6.76822E-08	****
DMSO	taf11	0.044	0.091	−0.23 (−0.33, −0.13)	1.92028E-05	****
DMSO	taf12	0.060	0.103	−0.19 (−0.29, −0.09)	4.90142E-04	***
DMSO	taf13	0.043	0.110	−0.35 (−0.45, −0.26)	1.43329E-10	****
DMSO	taf2	0.098	0.154	−0.18 (−0.28, −0.08)	6.91413E-04	***
DMSO	taf3	0.107	0.189	−0.23 (−0.32, −0.13)	2.30572E-05	****
DMSO	taf4	0.065	0.149	−0.29 (−0.39, −0.20)	1.06690E-07	****
DMSO	taf5	0.078	0.151	−0.25 (−0.35, −0.15)	3.22051E-06	****
DMSO	taf6	0.036	0.067	−0.24 (−0.34, −0.13)	1.07408E-05	****
DMSO	taf7	0.086	0.165	−0.27 (−0.37, −0.17)	7.22681E-07	****
DMSO	taf8	0.082	0.174	−0.28 (−0.38, −0.18)	2.99883E-07	****
DMSO	taf9	0.027	0.062	−0.36 (−0.45, −0.26)	1.43329E-10	****
DMSO	tbp	0.159	0.108	0.15 (0.04, 0.25)	6.60384E-03	**
DMSO	tfiia	0.150	0.113	0.06 (−0.04, 0.17)	2.51768E-01	ns
DMSO	tfiib	0.110	0.107	0.05 (−0.05, 0.15)	3.43056E-01	ns
DMSO	tfiif	0.099	0.095	0.05 (−0.05, 0.15)	3.43056E-01	ns
DMSO	mot1	0.025	0.032	−0.07 (−0.18, 0.03)	0.202550825	ns
DMSO	nc2a	0.079	0.023	0.40 (0.30, 0.49)	1.40418E-13	****
DMSO	nc2b	0.079	0.027	0.37 (0.27, 0.46)	7.48470E-12	****
DMSO	trf2	0.030	0.094	−0.42 (−0.51, −0.33)	6.06194E-15	****
TRI	taf1	0.089	0.169	−0.23 (−0.33, −0.13)	2.59774E-05	****
TRI	taf10	0.035	0.072	−0.28 (−0.37, −0.18)	7.98838E-07	****
TRI	taf11	0.079	0.135	−0.22 (−0.32, −0.12)	4.02554E-05	****
TRI	taf12	0.076	0.131	−0.19 (−0.29, −0.09)	3.02326E-04	***
TRI	taf13	0.069	0.143	−0.28 (−0.38, −0.18)	4.96360E-07	****
TRI	taf2	0.122	0.204	−0.21 (−0.32, −0.11)	6.66422E-05	****
TRI	taf3	0.118	0.221	−0.23 (−0.32, −0.13)	2.83330E-05	****
TRI	taf4	0.080	0.177	−0.26 (−0.36, −0.17)	1.96287E-06	****
TRI	taf5	0.098	0.180	−0.21 (−0.31, −0.11)	1.02381E-04	***
TRI	taf6	0.084	0.152	−0.25 (−0.34, −0.14)	9.91038E-06	****
TRI	taf7	0.128	0.256	−0.23 (−0.33, −0.13)	2.59774E-05	****
TRI	taf8	0.118	0.234	−0.24 (−0.34, −0.14)	1.19942E-05	****
TRI	taf9	0.046	0.086	−0.29 (−0.38, −0.19)	4.96360E-07	****
TRI	tbp	0.185	0.125	0.14 (0.04, 0.24)	8.23220E-03	**
TRI	tfiia	0.167	0.159	0.01 (−0.10, 0.11)	8.96040E-01	ns
TRI	tfiib	0.165	0.257	−0.13 (−0.23, −0.03)	1.45830E-02	*
TRI	tfiif	0.276	0.365	−0.07 (−0.17, 0.03)	1.75949E-01	ns

Table 3: Summary of Figure 4B and S7B statistics.

Group	Motif	Group n/N (%)	Other group n/N (%)	Fold enrichment (log2)	Adjusted p-value (Fisher)	Significance	Direction
broad	DPE	258/4545 (5.7%)	349/1769 (19.7%)	-1.8	3.06044e-57	****	depletion
broad	DRE	832/4545 (18.3%)	156/1769 (8.8%)	1.05	3.69011e-22	****	enrichment
broad	Inr	145/4545 (3.2%)	403/1769 (22.8%)	-2.84	6.04656e-119	****	depletion
broad	MTE	19/4545 (0.4%)	40/1769 (2.3%)	-2.44	2.62048e-10	****	depletion
broad	Motif1	164/4545 (3.6%)	31/1769 (1.8%)	1.04	7.41653e-05	****	enrichment
broad	Motif6	207/4545 (4.6%)	67/1769 (3.8%)	0.266	0.19153	ns	enrichment
broad	Motif7	774/4545 (17.0%)	177/1769 (10.0%)	0.767	8.56943e-13	****	enrichment
broad	PB	62/4545 (1.4%)	140/1769 (7.9%)	-2.54	1.096e-34	****	depletion
broad	TATA	10/4545 (0.2%)	79/1769 (4.5%)	-4.34	3.4837e-33	****	depletion
broad	TCT	33/4545 (0.7%)	78/1769 (4.4%)	-2.6	2.28998e-20	****	depletion
narrow	DPE	349/1769 (19.7%)	258/4545 (5.7%)	1.8	3.06044e-57	****	enrichment
narrow	DRE	156/1769 (8.8%)	832/4545 (18.3%)	-1.05	3.69011e-22	****	depletion
narrow	Inr	403/1769 (22.8%)	145/4545 (3.2%)	2.84	6.04656e-119	****	enrichment
narrow	MTE	40/1769 (2.3%)	19/4545 (0.4%)	2.44	2.62048e-10	****	enrichment
narrow	Motif1	31/1769 (1.8%)	164/4545 (3.6%)	-1.04	7.41653e-05	****	depletion
narrow	Motif6	67/1769 (3.8%)	207/4545 (4.6%)	-0.266	0.19153	ns	depletion
narrow	Motif7	177/1769 (10.0%)	774/4545 (17.0%)	-0.767	8.56943e-13	****	depletion
narrow	PB	140/1769 (7.9%)	62/4545 (1.4%)	2.54	1.096e-34	****	enrichment
narrow	TATA	79/1769 (4.5%)	10/4545 (0.2%)	4.34	3.4837e-33	****	enrichment
narrow	TCT	78/1769 (4.4%)	33/4545 (0.7%)	2.6	2.28998e-20	****	enrichment

Table 4: Summary of Figure S2C statistics. n = the number of promoters in that group that contain the motif and N = the total number of promoters in that group.

Factor	High Nc2 median	Low Nc2 median	Rank-biserial r (95% CI)	Adjusted p-value (Wilcoxon)	Significance
nc2a	2.793	1.137	0.92 (0.88, 0.96)	2.58355E-57	****
nc2b	2.885	1.402	0.99 (0.98, 1.00)	3.20183E-65	****
rpb3	5.897	6.051	−0.08 (−0.19, 0.03)	2.12439E-01	ns
taf1	3.418	4.012	−0.30 (−0.41, −0.19)	2.57314E-07	****
taf10	1.870	2.925	−0.47 (−0.56, −0.37)	2.52922E-15	****
taf11	2.467	3.166	−0.38 (−0.48, −0.28)	1.29227E-10	****
taf12	3.010	3.735	−0.33 (−0.43, −0.22)	1.03474E-08	****
taf13	2.423	3.651	−0.46 (−0.55, −0.36)	7.60143E-15	****
taf2	3.673	4.206	−0.25 (−0.36, −0.15)	1.16544E-05	****
taf3	3.874	4.541	−0.27 (−0.38, −0.16)	2.81150E-06	****
taf4	3.044	4.167	−0.40 (−0.51, −0.30)	5.70613E-12	****
taf5	3.267	4.177	−0.34 (−0.45, −0.23)	6.60012E-09	****
taf6	2.161	2.848	−0.37 (−0.47, −0.26)	2.78678E-10	****
taf7	3.439	4.453	−0.36 (−0.46, −0.25)	8.36099E-10	****
taf8	3.427	4.164	−0.33 (−0.44, −0.23)	9.84592E-09	****
taf9	1.828	3.063	−0.54 (−0.63, −0.45)	3.14658E-20	****
tbp	3.975	3.536	0.28 (0.17, 0.38)	2.21657E-06	****
tfiia	3.984	3.913	0.05 (−0.06, 0.16)	4.20631E-01	ns
tfiib	3.481	3.504	0.02 (−0.09, 0.13)	6.94080E-01	ns
tfiif	3.289	3.240	0.04 (−0.07, 0.15)	5.27677E-01	ns

Table 5: Summary of Figure S8C-D statistics.

Group	Motif	Comparison n/N (%)	Other group n/N (%)	Fold enrichment (log2)	Adjusted p-value (Fisher)	Significance	Direction
Low Nc2	TATA	0/203 (0.0%)	43/203 (21.2%)	-Inf	1.88541e-13	****	depletion
Low Nc2	Inr	60/203 (29.6%)	59/203 (29.1%)	0.0242	1	ns	enrichment
Low Nc2	MTE	6/203 (3.0%)	5/203 (2.5%)	0.263	1	ns	enrichment
Low Nc2	DPE	64/203 (31.5%)	38/203 (18.7%)	0.752	0.0204834	*	enrichment
Low Nc2	PB	25/203 (12.3%)	13/203 (6.4%)	0.943	0.199026	ns	enrichment
Low Nc2	TCT	9/203 (4.4%)	5/203 (2.5%)	0.848	0.593801	ns	enrichment
Low Nc2	DRE	8/203 (3.9%)	2/203 (1.0%)	2	0.243792	ns	enrichment
Low Nc2	Motif1	3/203 (1.5%)	2/203 (1.0%)	0.585	1	ns	enrichment
Low Nc2	Motif6	5/203 (2.5%)	1/203 (0.5%)	2.32	0.358782	ns	enrichment
Low Nc2	Motif7	19/203 (9.4%)	10/203 (4.9%)	0.926	0.243792	ns	enrichment
High Nc2	TATA	43/203 (21.2%)	0/203 (0.0%)	+Inf	1.88541e-13	****	enrichment
High Nc2	Inr	59/203 (29.1%)	60/203 (29.6%)	-0.0242	1	ns	depletion
High Nc2	MTE	5/203 (2.5%)	6/203 (3.0%)	-0.263	1	ns	depletion
High Nc2	DPE	38/203 (18.7%)	64/203 (31.5%)	-0.752	0.0204834	*	depletion
High Nc2	PB	13/203 (6.4%)	25/203 (12.3%)	-0.943	0.199026	ns	depletion
High Nc2	TCT	5/203 (2.5%)	9/203 (4.4%)	-0.848	0.593801	ns	depletion
High Nc2	DRE	2/203 (1.0%)	8/203 (3.9%)	-2	0.243792	ns	depletion
High Nc2	Motif1	2/203 (1.0%)	3/203 (1.5%)	-0.585	1	ns	depletion
High Nc2	Motif6	1/203 (0.5%)	5/203 (2.5%)	-2.32	0.358782	ns	depletion
High Nc2	Motif7	10/203 (4.9%)	19/203 (9.4%)	-0.926	0.243792	ns	depletion

Table 6: Summary of Figure S8E statistics. n = the number of promoters in that group that contain the motif and N = the total number of promoters in that group.

Treatment	Factor	Q1 median	Q5 median	Rank-biserial r (95% CI)	Adjusted p-value (Wilcoxon)	Significance
DMSO	tfiia	3.523	2.626	0.41 (0.32, 0.50)	3.42398E-15	****
DMSO	tfiib	3.990	2.374	0.66 (0.59, 0.73)	2.33129E-38	****
DMSO	tfiif	3.740	2.168	0.67 (0.60, 0.74)	6.71810E-39	****
DMSO	tbp	3.299	3.074	0.21 (0.11, 0.30)	3.64445E-05	****
DMSO	taf10	2.432	2.038	0.22 (0.13, 0.32)	1.38788E-05	****
DMSO	taf4	3.685	3.055	0.31 (0.22, 0.41)	8.72893E-10	****
DMSO	taf12	3.447	2.692	0.38 (0.29, 0.47)	1.58675E-13	****
DMSO	taf5	3.835	2.985	0.34 (0.25, 0.44)	2.29030E-11	****
DMSO	taf9	2.398	2.018	0.23 (0.13, 0.32)	6.72303E-06	****
DMSO	taf13	2.804	2.550	0.14 (0.05, 0.24)	5.00542E-03	**
DMSO	taf11	2.708	2.320	0.27 (0.18, 0.37)	8.16288E-08	****
DMSO	taf6	2.558	1.976	0.37 (0.28, 0.46)	9.41548E-13	****
DMSO	taf8	3.677	3.259	0.15 (0.06, 0.25)	2.73529E-03	**
DMSO	taf1	3.726	2.979	0.35 (0.26, 0.44)	1.39358E-11	****
DMSO	taf2	3.947	3.395	0.27 (0.18, 0.37)	1.18261E-07	****
DMSO	taf7	4.015	3.258	0.35 (0.25, 0.44)	1.48181E-11	****
DMSO	taf3	4.193	3.499	0.31 (0.22, 0.40)	1.52115E-09	****
DMSO	rpb3	5.427	5.752	−0.13 (−0.22, −0.03)	1.19797E-02	*
DMSO	nelf	4.579	5.628	−0.34 (−0.43, −0.25)	2.52710E-11	****

Table 7: Summary of Figure S9B statistics.

Target	Host	Type	Source	Identifier	Region
TBP	Rabbit	Custom polyclonal	Zeitlinger Lab (GenScript)	U4123BK150-6 (1738)	Full-length protein
TAF1	Rabbit	Custom polyclonal	Zeitlinger Lab (GenScript)	U7893CL140-11	Peptide: DEGSIGNGLDLTGIL
TAF2	Rabbit	Custom polyclonal	Zeitlinger Lab (GenScript)	U4123BK150-9	aa 640–971
TAF3	Rabbit	Custom polyclonal	Zeitlinger Lab (GenScript)	U7724CL140-9	aa 133–319
TAF4	Rabbit	Custom polyclonal	Zeitlinger Lab (GenScript)	U7893CL140-3	aa 209–383
TAF5	Rabbit	Custom polyclonal (affinity purified)	Zeitlinger Lab (GenScript)	U7893CL140-5 AP	N-terminal aa 24–135
TAF6	Rabbit	Custom polyclonal	Zeitlinger Lab (GenScript)	U1266BL120-3	C-terminus
TAF7	Rabbit	Custom polyclonal	Zeitlinger Lab (GenScript)	U1266BL120-6	C-terminus
TAF8	Rabbit	Custom polyclonal	Zeitlinger Lab (GenScript)	U7893CL140-7	C-terminal aa 223–328
TAF9	Rabbit	Custom polyclonal	Zeitlinger Lab (GenScript)	529220-34	aa 1–188
TAF10	Rabbit	Custom polyclonal	Zeitlinger Lab (GenScript)	U7970DB220-5	aa 1–93
TAF11	Rabbit	Custom polyclonal	Zeitlinger Lab (GenScript)	U1266BL120-9	aa 1–98
TAF12	Rabbit	Custom polyclonal	Zeitlinger Lab (GenScript)	U7970DB220-1	Peptide: CMSDLFTTFDSNGVAEHHLHHNHNSTSSASGLLHDPP
TAF13	Rabbit	Custom polyclonal	Zeitlinger Lab (GenScript)	U3982CL190-3	Full-length protein
TBP	Rabbit	Custom polyclonal	James Kadonaga Lab	517	-
NC2α	Rabbit	Custom polyclonal	James Kadonaga Lab	NC2a_14940	-
NC2β	Rabbit	Custom polyclonal	James Kadonaga Lab	NC2b_14915	-
Mot1	Rabbit	Custom polyclonal	James Kadonaga Lab	Mot1_3860	-
TRF2	Rabbit	Custom polyclonal	James Kadonaga Lab	TRF2_275	-

Table 8: Summary of the antibodies used for the ChIP-nexus experiments.
