# Supplementary material for: High-resolution binding data of TFIID and cofactors show promoter-specific differences
Source: Nat Commun. 2026 Oct 7;17:11446. doi: 10.1038/s41467-026-77758-9 (PMC13736863; doi:10.1038/s41467-026-77758-9)
Supplement: Supplementary file 2 [file 41467_2026_77758_MOESM2_ESM.pdf]

nature portfolio

Corresponding author(s): Julia Zeitlinger, Ph.D.

Last updated by author(s): 07/22/2026

Reporting Summary

Nature Portfolio wishes to improve the reproducibility of the work that we publish. This form provides structure for consistency and transparency in reporting. For further information on Nature Portfolio policies, see our Editorial Policies and the Editorial Policy Checklist.

Statistics

For all statistical analyses, confirm that the following items are present in the figure legend, table legend, main text, or Methods section.

n/a	Confirmed	
☐	☒	The exact sample size (n) for each experimental group/condition, given as a discrete number and unit of measurement
☐	☒	A statement on whether measurements were taken from distinct samples or whether the same sample was measured repeatedly
☐	☒	The statistical test(s) used AND whether they are one- or two-sided<br>*Only common tests should be described solely by name; describe more complex techniques in the Methods section.*
☒	☐	A description of all covariates tested
☐	☒	A description of any assumptions or corrections, such as tests of normality and adjustment for multiple comparisons
☐	☒	A full description of the statistical parameters including central tendency (e.g. means) or other basic estimates (e.g. regression coefficient) AND variation (e.g. standard deviation) or associated estimates of uncertainty (e.g. confidence intervals)
☐	☒	For null hypothesis testing, the test statistic (e.g. F, t, r) with confidence intervals, effect sizes, degrees of freedom and P value noted<br>*Give P values as exact values whenever suitable.*
☒	☐	For Bayesian analysis, information on the choice of priors and Markov chain Monte Carlo settings
☐	☒	For hierarchical and complex designs, identification of the appropriate level for tests and full reporting of outcomes
☐	☒	Estimates of effect sizes (e.g. Cohen's d, Pearson's r), indicating how they were calculated

Our web collection on statistics for biologists contains articles on many of the points above.

Software and code

Policy information about availability of computer code

Data collection	All protein structures, the DNA-bound human TFIID (pdb_00006mzm), yeast TFIID (pdb_00006hqa), TFIIA-TBP (pdb_00001nvp), TAF1-7-2 (pdb_00006mzm), TAF4-12 (pdb_00001h3o), TAF6-9 (pdb_00006mzd), TAF11-13 (pdb_00001bh8) and NC2-TBP-DNA (pdb_00001jfi), were downloaded from the RCSB Protein Data Bank and colored appropriately using UCSF CHIMERA121. CAGE-seq data were downloaded from modENCODE (ENCSR827COJ). Previously published TBP, Poll II and Nelf ChIP-nexus are available under GEO accession GSE85741.
Data analysis	The analysis code is available on GitHub at https://github.com/zeitlingerlab/G-M_Alcantara_TFIID_2025

For manuscripts utilizing custom algorithms or software that are central to the research but not yet described in published literature, software must be made available to editors and reviewers. We strongly encourage code deposition in a community repository (e.g. GitHub). See the Nature Portfolio guidelines for submitting code & software for further information.

Data

Policy information about availability of data

All manuscripts must include a data availability statement. This statement should provide the following information, where applicable:

- Accession codes, unique identifiers, or web links for publicly available datasets
- A description of any restrictions on data availability
- For clinical datasets or third party data, please ensure that the statement adheres to our policy

The raw RNA-seq and ChIP-nexus data published in this study are available under GEO accession number GSE314461. Gene expression table counts and ChIP-nexus tracks are readily available under the same GEO accession. The analysis code is available on GitHub at https://github.com/zeitlingerlab/G-M_Alcantara_TFIID_2025.

Research involving human participants, their data, or biological material

Policy information about studies with human participants or human data. See also policy information about sex, gender (identity/presentation), and sexual orientation and race, ethnicity and racism.

Reporting on sex and gender	N/A
Reporting on race, ethnicity, or other socially relevant groupings	N/A
Population characteristics	N/A
Recruitment	N/A
Ethics oversight	N/A

Note that full information on the approval of the study protocol must also be provided in the manuscript.

Field-specific reporting

Please select the one below that is the best fit for your research. If you are not sure, read the appropriate sections before making your selection.

- [x] Life sciences
- [ ] Behavioural & social sciences
- [ ] Ecological, evolutionary & environmental sciences

For a reference copy of the document with all sections, see nature.com/documents/nr-reporting-summary-flat.pdf

Life sciences study design

All studies must disclose on these points even when the disclosure is negative.

Sample size	All samples were genome-wide experiments.
Data exclusions	Only replicates passing our library and alignment quality controls are reported.
Replication	Experiments were performed in at least two biological replicate experiments.
Randomization	N/A
Blinding	N/A

Reporting for specific materials, systems and methods

We require information from authors about some types of materials, experimental systems and methods used in many studies. Here, indicate whether each material, system or method listed is relevant to your study. If you are not sure if a list item applies to your research, read the appropriate section before selecting a response.

Materials & experimental systems

n/a	Involved in the study	
[ ]	[x]	Antibodies
[ ]	[x]	Eukaryotic cell lines
[x]	[ ]	Palaeontology and archaeology
[x]	[ ]	Animals and other organisms
[x]	[ ]	Clinical data
[x]	[ ]	Dual use research of concern
[x]	[ ]	Plants

Methods

n/a	Involved in the study	
[ ]	[x]	ChIP-seq
[x]	[ ]	Flow cytometry
[x]	[ ]	MRI-based neuroimaging

Antibodies

Antibodies used

The following antibodies were used in the study and are made available upon request to the scientific community.

Custom polyclonal rabbit antibodies against TFIID subunits were designed based on prior knowledge and made by Genscript:
custom anti-TBP rabbit polyclonal, Zeitlinger Lab, TBP - U4123BK150-6 (1738), full length
custom anti-TAF1 rabbit polyclonal, Zeitlinger Lab, TAF1 (P1) - U7893CL140-11, short peptide DEGSIGNGLDLTGIL
custom anti-TAF2 rabbit polyclonal, Zeitlinger Lab, TAF2 - U4123BK150-9, aa 640-971

custom anti-TAF3 rabbit polyclonal, Zeitlinger Lab, TAF3 - U7724CL140-9, aa 133-319
custom anti-TAF4 rabbit polyclonal, Zeitlinger Lab, TAF4 - U7893CL140-3, aa 209-383
custom anti-TAF5 rabbit polyclonal, Zeitlinger Lab, TAF5 (Affinity purified) - U7893CL140-5AP, N-term aa 24-135
custom anti-TAF6 rabbit polyclonal, Zeitlinger Lab, TAF6 - U1266BL120-3, C-term
custom anti-TAF7 rabbit polyclonal, Zeitlinger Lab, TAF7 - U1266BL120-6, C-term
custom anti-TAF8 rabbit polyclonal, Zeitlinger Lab, TAF8 - U7893CL140-7, C-term aa 223-328
custom anti-TAF9 rabbit polyclonal, Zeitlinger Lab, TAF9 - 529220-34, aa 1-188
custom anti-TAF10 rabbit polyclonal, Zeitlinger Lab, TAF10 - U7970DB220-5, aa 1-93
custom anti-TAF11 rabbit polyclonal, Zeitlinger Lab, TAF11 - U1266BL120-9, aa 1-98
custom anti-TAF12 rabbit polyclonal, Zeitlinger Lab, TAF12 - U7970DB220-1, short peptide CMSDLFTTFDSNGVAEHHLHHNHNSTSSASGLLHDPP
custom anti-TAF13 rabbit polyclonal, Zeitlinger Lab, TAF13 - U3982CL190-3, full length

Other antibodies used in this study were a gift from James Kadonaga's lab:
custom anti-TBP rabbit polyclonal, Kadonaga Lab, TBP - Kadonaga lab 517
custom anti-NC2a rabbit polyclonal, Kadonaga Lab, NC2a_14940
custom anti-NC2b rabbit polyclonal, Kadonaga Lab, NC2b_14915
custom anti-Mot1 rabbit polyclonal, Kadonaga Lab, Mot1_3860
custom anti-TRF2 rabbit polyclonal, Kadonaga Lab, TRF2_275

Validation: No validation by Western Blot was performed. The specificity of the antibodies were confirmed by ChIP-nexus promoter signal.

Eukaryotic cell lines

Policy information about cell lines and Sex and Gender in Research

Cell line source(s): Drosophila melanogaster Kc167 cells were purchased from DGRC.

Authentication: Kc167 cells were purchased from DGRC and not authenticated.

Mycoplasma contamination: Cells tested negative for mycoplasma contamination.

Commonly misidentified lines (See ICLAC register): N/A

Plants

Seed stocks: N/A

Novel plant genotypes: N/A

Authentication: N/A

ChIP-seq

Data deposition

☒ Confirm that both raw and final processed data have been deposited in a public database such as GEO.

☐ Confirm that you have deposited or provided access to graph files (e.g. BED files) for the called peaks.

Data access links (*May remain private before publication.*): https://www.ncbi.nlm.nih.gov/geo/query/acc.cgi?acc=GSE314461

Files in database submission:
Raw sequencing fastq files per replicate
Bigwig files per replicate, raw signal of ChIP-nexus stop bases, non-normalized

Genome browser session (e.g. UCSC): *Provide a link to an anonymized genome browser session for "Initial submission" and "Revised version" documents only, to enable peer review. Write "no longer applicable" for "Final submission" documents.*

Methodology

Replicates: Two replicates are provided for each protein and treatment (except non-treated Mot1 has only one replicate).

Sequencing depth: Refer to GEO accession

Antibodies	Refer to the Antibodies section
Peak calling parameters	N/A
Data quality	*Describe the methods used to ensure data quality in full detail, including how many peaks are at FDR 5% and above 5-fold enrichment.*
Software	The analysis code is available on GitHub at https://github.com/zeitlingerlab/G-M_Alcantara_TFIID_2025
