# Supplementary material for: High-resolution binding data of TFIID and cofactors show promoter-specific differences
Source: Nat Commun. 2026 Oct 7;17:11446. doi: 10.1038/s41467-026-77758-9 (PMC13736863; doi:10.1038/s41467-026-77758-9)
Supplement: Supplementary file 3 [file 41467_2026_77758_MOESM3_ESM.pdf]

nature portfolio

Peer Review File

High-resolution binding data of TFIID and cofactors show promoter-specific differences

Corresponding Author: Professor Julia Zeitlinger

This file contains all reviewer reports in order by version, followed by all author rebuttals in order by version.

Version 0:

Reviewer comments:

Reviewer #1

(Remarks to the Author)
In this paper the Zeitlinger lab uses their ChIP-NEXUS pipeline to analyze how TBP and TBP-associated factors (TAFs) crosslink to promoters in Drosophila KC167 cells. The distinct patterns match well with, and provide in vivo validation for, published structural models suggesting that different TFIID conformations exist. The crosslinking patterns correlate well with particular promoter sequences and behaviors. The data is clear and the interpretations are reasonable. Overall, this is a very nice paper that should be of wide interest to the gene expression community. I have some relatively minor comments and suggestions to consider before finalizing the paper for publication.

1. The figure legend for Fig 1C says both strands are shown in the same color. But only one heatmap is shown per TAF, which I assume is only one strand. Are the signals from the two strands are superimposed? It wouldn't make sense to show the data that way. Please clarify.

2. Fig 1D. I recommend adding the Correlation coefficients (currently shown in the supplemental figure) into the graph.

3. Line 86. I think it overstates things to say that TFIID structure is inferred from crosslinking. The crosslinking is consistent with published structures, but I don't think you could produce the same model based on crosslinks alone. It would be more accurate to say the crosslinks reveal TFIID "conformation" rather than "structure".

4. Line 140. The paper says that TBP primarily interacts with the phosphate backbone as an explanation for why there are no crosslinks up near TATA box. This isn't accurate. TBP makes extensive hydrophobic interactions with bases in the minor groove, and two phenylalanines actually intercalate between TATA box bases. More likely, crosslinking is mostly mediated by base amines in the major groove, but not the minor groove where TBP is binding.

5. Fig 2A: The figure legends don't mention the two black arrows pointing to peaks in the downstream and narrow upper traces. These should either be explained or removed.

6. Fig 2A and B: It might be useful to include the crosslinking and PC patterns for TFIIA and TFIIB for comparison, since both are direct TBP interactors. These are shown in a Fig 4, where it's helpful for contrast to Mot1 and NC2. Also, in Fig 2C it could be helpful to label lobes A, B, and C in the figure, not just describe them in the legend.

7. Fig 3B,C and Sup Fig S5C, D: These figures compare promoter classifications that are based on sequence motifs with those derived from clustering of the TBP crosslinking patterns. However, the same names (i.e. TATA, dpr, and hk) are used for both types of classifications, which is confusing. There's no reason to presume the two approaches would give identical classes, so using the same names hides the significance of the finding. It would be clearer if the two different classification methods were distinguished by using a subscript or other marker (maybe something like: "dpr-seq" versus "dpr-TBP").

8. Line 223 and 234. I don't follow the logic of the argument the authors present for concluding that TBP dissociates with each round of transcription. ChIP-NEXUS can measure steady-state occupancy, but not kinetics. Stronger TBP crosslinking could mean a longer dwell time per binding event, or more frequent binding events per unit time. In either case, I would expect a correlation with other GTFs and transcript output (CAGE).

9. Fig 4A. You can't really see what Mot1 is doing near the TATA box, as the signal is hidden behind NC2.

10. Line 355, 362. The cited paper from the Cramer lab does claim TBP dissociates upon elongation, but the authors may want to make clear that this paper used purified GTFs with TBP. There is no activator, Mediator, or TAFs. Pointing out the absence of TAFs will better lead into their discussion a little later about TFIID loading TBP.

11. The Discussion (line 359) says TATA promoters have "larger transcriptional bursts" than non-TATA promoters. I think this conflates burst size/amplitude with bursting behavior. My understanding is that non-TATA promoters don't show any bursts. One model from yeast work (summarized in reference 10) is that TAFs remain bound through multiple rounds of transcription to keep nucleosomes from shutting off the promoter, while TBP (without TAFs) dissociates to create off-periods around the bursts. Bursting is defined by the "off" periods as much as the production of multiple transcripts during active periods.

(Remarks on code availability)

Reviewer #2

(Remarks to the Author)
In this manuscript, Alcantara et al. profile the genomic footprints of the 14 subunits of TFIID and several general transcription factors in Drosophila cells at high resolution. These results show concordance with structural models of TFIID binding to the core promoter and provide a complementary view of TFIID's structural rearrangements. Their analysis reveals that TBP has the most promoter-specific binding patterns out of all TFIID subunits, to an extent that it can be used for promoter classification and to recover sequence motifs such as the TATA box. Profiling of TBP regulators revealed a specificity for the regulator NC2 for TATA promoters. They also provide evidence that, in cells, TBP can seed formation of the PIC in the absence of TAFs. Overall, the manuscript is well-written and appropriately referenced. The ChIP-nexus dataset is of high quality, complementary to prior structural studies, and could be a valuable resource to the field. The observation of TAF-independent transcription initiation in cells is a key result and provides valuable data into a mechanistic question that continues to be debated. However, we do have a few major concerns that must be addressed before we can support publication.

Major points:

1. The authors argue through the paper that their results agree very well with the available cryo-EM structures, but they seem to cherry pick from their data and from the information that can be deduced from the structure. For example, if one looks at the intensity displayed in Figure 1D, TAF4 and TAF12 are grouped together because they basically do not correlate strongly with anything else (the PCC between TAF4/TAF12 is also not that high). Both are known to form an integral part of two structural lobes of TFIID, so we are surprised they do not correlate strongly with anything else. We would expect them to correlate strongly with each other and with TFIIA and TBP, so why do they have such distinct behavior? We have the impression that the authors pick and choose what data fit with the structural work. Related to this, the discussion of Figure 1D with respect to the clustering and interpretation is confusing (see minor point 10).

2. One of the subsections is entitled "TFIID structure inferred from in vivo footprints". The authors really should tone this down. Maybe change it to something like "In vivo footprints are in agreement with TFIID structure" These data would not be able to define TFIID structure by itself.

3. The question of whether TAF footprints differ at different promoters is not adequately explored. Although TBP does show the clearest differences in ChIP-nexus signal at different promoter types, there are differences among the other TAFs, which are only briefly acknowledged by the authors in lines 170-171. In Figure S6, not only do many TAFs have more diffuse footprints in TCT/HK promoters, but the peaks appear to shift (for example, TAF5 and TAF3 upstream regions). Related to this, while TAF footprints may be similar for TATA vs. DPR vs. TCT vs. HK promoters, the authors do not consider the possibility that partitioning promoters based on other criteria besides sequence motifs may reveal differences. For example, the authors suggest that TAF3's somewhat unexpected downstream footprint may arise in part due to its interaction with H3K4me3, so it would be interesting to evaluate whether its footprint differs at promoters with high or low H3K4me3.

4. The observation that TBP has a downstream footprint, and that this footprint disappears after triptolide treatment, has previously been reported by the same research group in ref. 30 (Shao & Zeitlinger, Nat. Genet. 2017). However, in their discussion of this result (see lines 174-181, 321-325, 337), this paper is not cited. While we appreciate that this observation can now be better contextualized due to recent cryo-EM structures that were not available at the time of the earlier study, the authors should make it clear in the text which observations have been previously observed and thus are not fully novel.

5. The authors argue that since TBP does not show significantly higher normalized binding at TATA vs. DPR promoters (Figure 4B), it must not remain bound at the promoter through multiple cycles of initiation. However, this analysis seems to be flawed since it compares total ChIP-nexus signal in a window spanning the entire core promoter, which includes signal from non-engaged states (i.e., the +32 signal corresponding to a "loading" state). Instead, it would be more appropriate to compare ChIP-nexus signal around the -18 upstream region corresponding to engaged TBP, since if TBP were to remain engaged after TAF dissociation, one would expect it to remain bound at the TATA box. Figure 4A shows that binding

specifically in the -18 region is higher for TATA vs. DPR promoters, so the authors cannot conclusively rule out the possibility that TBP remains bound to the TATA sequence in TATA promoters after the other TAFs dissociate.

6. The above point is still compatible with the authors' conclusion that the PIC can assemble around TBP in the absence of TAFs, which is based on their comparison of normalized TAF binding at TATA vs. DPR promoters. However, given the data in the manuscript, it is not possible to distinguish between a mechanism (1) where TBP is able to bind by itself to a TATA promoter de novo, versus (2) where TAFs initially load TBP and then dissociate so that future cycles of PIC formation use TBP that remains bound to the TATA sequence. The authors should carefully revise the text to acknowledge this ambiguity and avoid unqualified statements like "TBP can directly bind to the TATA box" (line 298), since their data do not directly support this claim.

7. It would be interesting to look at whether TATA promoters with higher NC2 signal vs. lower NC2 signal have lower binding of PIC components, TBP, and/or TAFs. This analysis should be straightforward to perform using the existing data, and could help strengthen the authors' conclusions regarding NC2.

Minor points:

8. It would be good if the authors can clarify at the first point in the text where CAGE-seq is mentioned that this is a publicly available dataset from modENCODE that has been reanalyzed, as opposed to new data collected in this study.

9. Concerning the analysis of the data, can the authors comment on what they mean when they refer to the "corresponding peak" in the other strand (line 97)? What does the difference between the position in the two strands say? Does it relate to the size of protein?

10. In lines 76-77, the authors say "TBP had a higher correlation with TFIIA and other PIC components than with the TAFs (Figure 1D)." However, the individual PCCs for TBP and Pol II/TFIIF/TFIIB appear to be weaker than for TBP and some of the TAFs. It seems like the authors are referring instead to the fact that TBP clusters with TFIIA/Pol II/TFIIF/TFIIB based on all the pairwise PCCs. Similarly, in line 80, the authors refer to "the most correlated TAFs (TAF4/12, TAF1/7/2, TAF11/13 and TAF6/9)…" but the PCC for TAF4 and TAF12 is not very high. It would be good for the authors to use more precise wording when referring to the hierarchical clustering.

11. For any figures in which structural models are shown, the authors should at minimum cite the PDB IDs in the figure legend. This applies to Figures 1D, 2C, and S8B.

12. In Fig. 2B, TAF9 appears as an outlier in the PCA analysis shown, and so is TBP. It is not clear that the "upstream narrow" cluster is well-defined, as TAF9 could just as easily be considered part of the "upstream broad" cluster.

13. Concerning the results in Fig. 2C, the authors state that "both the modular organization and the preferred DNA contacts align well with cryo-EM structures of promoter-bound human and yeast TFIID" (lines 101-102), but there are obvious exceptions. For example, TAF11 does not belong at all to the TAF6/8 module that is supposed to be in the middle. Therefore, the following statement, "demonstrating that our data can provide insights into the structural organization of the complex in vivo" (line 103) should be toned down.

14. Can the authors comment on why TAF13 does not follow the same pattern as TAF11?

15. The authors state, "TBP primarily interacts with the DNA phosphate backbone, rather than the bases, which are the preferred substrate of formaldehyde crosslinking" (lines 140-141). This is a strange argument, as TBP is one of the TFIID subunits that actually recognizes sequences. The argument could then be applied to most of the TAFs.

16. The observation that TAF4 has a broad footprint in the upstream proximal promoter (discussed in lines 145-150) is a really nice finding that complements prior biochemical findings. TAF4 seems to be a major hotspot for interactions with transcriptional activators, possibly more than other TAFs. We think it would be good to emphasize this more in the discussion of this finding. Consider also citing the following relevant papers: PMIDs 17483474 and 23873942.

17. The authors do not comment on the fact that TAF12, and especially TAF4, also have a peak at around -18/-14. Interestingly, this is a site where conserved positive residues in TAF4 contact the DNA in the structure.

18. In Figure 2B,C, the downstream TAF cluster consists of those TAFs unique to lobe C (TAF2/1/7), along with TAF3. This is surprising, since TAF3 is known to move with TAF5,10,12,4,6,9,11,13 in lobe A to be everywhere, including upstream when in the process of engaging upstream DNA. Could the authors comment further on this?

19. The Figure 2C legend states that these structures are crystal structures, but in fact they are cryo-EM structures.

20. In Figure 3D, the color key for the negative strand should be fixed so that it is blue instead of gray.

21. In the reporting summary, the authors state under the Data Exclusions section that "Only experiments that worked were selected as samples." Could the authors clarify what they mean here?

(Remarks on code availability)

Reviewer #3

(Remarks to the Author)
n this manuscript, Alcantara et al. present a comprehensive, high-resolution binding atlas of the complete TFIID complex and associated factors (TBP, NC2, Mot1, TRF2) in Drosophila cells using ChIP-nexus. By generating base-pair resolution footprints, the authors validate that the in vivo modular organization of TFIID aligns remarkably well with recent cryo-EM structures and reveal distinct promoter-specific binding modes: a downstream loading state prevalent at DPR promoters and a specific upstream TBP engagement at TATA promoters facilitated by NC2. This work provides valuable in vivo evidence for TFIID footprints and proposes a dual mechanism of transcription initiation—TAF-dependent loading versus TAF-independent direct TBP binding—offering a potential mechanistic explanation for the distinct transcriptional bursting properties observed at TATA-containing genes. While the data quality is exceptional and the study addresses long-standing questions regarding TFIID function, the interpretation of these results requires more caution before publication.
Major Concerns
1. TAF-independent initiation and dynamic turnover:
The authors conclude that TATA promoters utilize a TAF-independent initiation mechanism, primarily because TAF occupancy (normalized to CAGE-seq transcript levels) is lower at TATA promoters compared to DPR promoters (Figure 4). While the validity of the observed lower occupancy is not in question, the interpretation of this result as independence is potentially missleading.
Transcription initiation is a dynamic cycle involving the association and dissociation of factors, whereas ChIP-nexus provides a static snapshot of steady-state occupancy. A lower steady-state signal at TATA promoters does not necessarily imply that TAFs are not required or not present during the initiation event; it could alternatively indicate that the PIC is highly dynamic with a rapid turnover rate (high dissociation/re-initiation frequency). In this scenario, TAFs might be essential but their residence time is short, resulting in a lower captured signal compared to the more stable complexes at DPR promoters. Therefore, the distinction between TAF-dependent and TAF-independent might be better explained by kinetic differences rather than a fundamental structural binary. The authors should discuss these dynamic possibilities (association/dissociation kinetics) and avoid presenting the TAF-independent model as an absolute dichotomy to avoid misleading readers regarding the underlying mechanism.
2. The TBP footprint location:
The authors observe the strongest TBP footprint at -18 bp rather than the TATA box (-30 bp), attributing the lack of signal at -30 bp to poor crosslinking of TBP to the DNA phosphate backbone. However, they simultaneously show a sharp NC2 footprint at -33 bp. If NC2 binds the TBP-DNA complex as described in the structural model, it is puzzling why the NC2 signal captures the -30 bp region while TBP does not. This discrepancy raises questions about what the -18 bp footprint actually represents.
The authors suggest the -18 bp signal might come from interactions with other PIC components (e.g., TFIIB). If the TBP signal is indeed indirect, this complicates the proposed model of "direct TBP binding" (Figure 5). The authors should reconcile the presence of the NC2 footprint at the TATA box with the absence of a TBP footprint at the same location to substantiate their structural conclusions.
3. The loading state and the effect of Triptolide:
The identification of the downstream module (+10 to +32 bp) as a "TFIID loading state" relies on its disappearance upon Triptolide treatment. Since Triptolide inhibits TFIIH and causes Pol II arrest shortly after initiation, it is crucial to clarify whether the disappearance of the downstream TBP/TAF signal is due to a genuine conformational change (transition to the upstream state) or simply a steric effect where the arrested Pol II occludes the downstream binding site.
4. NC2 and Transcriptional Bursting:
The authors propose a model where NC2 and the specific TBP binding mode contribute to the large burst sizes observed at TATA promoters. While this is a compelling hypothesis, the current link is largely correlative. The manuscript would benefit from providing more evidence or a deeper analytical connection between NC2 binding strength and bursting parameters to move this claim beyond speculation.

(Remarks on code availability)

Reviewer #4

(Remarks to the Author)
I co-reviewed this manuscript with one of the reviewers who provided the listed reports. This is part of the Nature Communications initiative to facilitate training in peer review and to provide appropriate recognition for Early Career Researchers who co-review manuscripts.

(Remarks on code availability)

Reviewer #5

(Remarks to the Author)

In this study, Alcantara et al. define a comprehensive binding map of transcription initiation complexes in Drosophila using ChIP-nexus. This work reports high-quality, spatially resolved datasets for TFIID subunits that will clearly be valuable to the field. Their attempt to infer structural configuration from ChIP-nexus footprints is an ambitious and exciting application of the method at the interface of structural biology and genomics. However, the manuscript requires substantial revision, including reanalysis and reconsideration of several interpretations, before it seems suitable for publication.

Selection of narrow TSSs

The authors define "narrow promoters" based on CAGE-defined TSS width and focus most analyses on this subset, which accounts for roughly one-fourth of all active promoters. Although this filtering could significantly affect the results, the authors do not clarify the motivation for this selection nor do they evaluate the effects caused by this selection. For example, narrow TSSs (<10bp) are known to be enriched for containing a TATA-box, which could bias their analyses on subunit positioning.

1. Rationales for the narrow TSS selection

The authors should explain why they restrict the analyses to narrow TSSs. If the concern is that including all promoters would blur the signal and complicate spatial inference, they should show what the signal across all active promoters looks like and demonstrate why this filtering is required and how it is justified.

2. Confounding effects from selecting narrow TSSs

It is unclear whether the module positions for each subunit (Fig. 2) are conserved across promoter classes or are specific to TATA-box promoters. For example, if TATA-box promoters are excluded, do the profiles remain similar? If the Fig.2 results are specific to TATA-box promoters, the figure and text should be reframed explicitly as describing configuration at TATA-box promoters. In addition, Fig. 2C should clarify whether the cryo-EM structure shown is derived from a TATA-box promoter or a TATA-less promoter.

Structural inference from ChIP-nexus data.

This section is particularly interesting because the authors propose that ChIP-nexus data could be used to predict structural relationships between components. However, to support this approach, it requires further clarification and validation.

1. Effects of triptolide treatment

The authors use triptolide treatment to enhance ChIP enrichment. Although a limited comparison between DMSO and triptolide conditions is shown (e.g. Fig. S6), it remains unclear how strongly the treatment alters footprint profiles. Notably, Fig. 2 appears to rely only on the triptolide condition, but the conclusions would be more convincing if the authors showed that the same overall spatial relationships hold without treatment. It would be useful to provide a PCA plot and average binding profiles (same as Fig. 2B and Fig. S4, respectively) using non-treated condition.

2. Module classification

The authors use PCA to characterize spatial profiles of TFIID subunits, but it was not clear to this reviewer from the Methods section how they group these factors into four modules. Is it manually grouped based on the PCA plot or is a clustering method used? In addition, the average positions of fragment midpoints (or an equivalent quantitative positional summary) should be shown to assess whether the footprint positions correlate with the structural configuration.

Downstream TBP binding

The authors emphasize a TBP binding peak downstream of TSS and interpret it in the context of a TFIID loading state model. However, because ChIP-nexus signal can be highly influenced by crosslinking to nearby proteins (as the authors note), it is not clear that this downstream peak truly reflects loaded TBP.

Based on the observation that triptolide diminishes the downstream TBP peak (Fig. S6), the authors link this peak with TFIID loading state. However, other TAF subunits also seem to show reduced downstream signal (TAF4/13/2/1/3 in Fig. S6). Given that the TBP crosslinking can be influenced by other proteins in the proximity, further evidence is required to support the conclusion that reduced downstream TBP signal specifically indicates loss of TBP loading by TAFs.

Normalization signal with CAGE reads

The authors normalize the ChIP-nexus signal at individual promoters by transcript levels measured by CAGE. CAGE detects transcript start site from total RNAs (mostly cytoplasmic) and does not directly reflect the rate of transcription initiation events per promoter. CAGE signal is influenced not only by initiation but also by pausing, elongation, RNA half-life, etc. Moreover, transcription output is a consequence of subunit binding, so it does not seem intuitive to divide binding strength by CAGE mRNA expression. It would be clearer if the authors provide the similar analyses by binning promoters by expression levels (to control the transcriptional activity) but still using non-normalized binding values.

"TAF-dependent" versus "TAF-independent" mechanisms – lack of validation

The authors describe "TAF-independent" initiation at TATA-box promoters (Line 264-267). Although CAGE-normalized TAF binding is reduced at these promoters, absolute TAF signal is still high and even at comparable levels for several TAFs (e.g. 1/2/3/4/13) (Fig. 3B, Fig. S6). Thus, the current correlative analyses do not support a conclusion of "TAF independence." Without experimental validation (e.g., TAF perturbation), the claim should be removed /toned down and the term "TAF-independent" not used. Also, the reduction in TAF-binding at TATA-box promoters can be strongly observed particularly in normalized values potentially due to the high CAGE signal at TATA-box promoters (worth checking it).

The authors propose that two distinct mechanisms at TATA-box promoters, TAF-dependent and TAF-independent PIC formation (Line 275-279), and highlight the resulting model summary in Fig.5. Given the limited experimental evidence, this conclusion is too strongly stated. To properly demonstrate two distinct modes, they would need evidence that can distinguish different states at the same promoter such as single-molecule measurements. Otherwise, they should point to this possibility not in the result but speculatively in the discussion and schematic model needs to be removed.

Minor comments:

- Several structural models are shown in the figures; the legends should cite the studies from which each structure was obtained.
- Line 32: "transcriptional initiation" is used, while in other parts, "transcription initiation". Please use one term consistently.

• Line 62 should refer to the results obtained in this study, not only references.
• In Fig. 1C, promoters are sorted by TAF1 signal. To represent the signal without being biased to one sample, it would be fair to sort the protomers based on CAGE tpm or an independent metrics.
• Line 69-72: the statement on SAGA complex is unclear. The authors indicate that correlations among TAF9/10/12 are not apparent likely due to the TAF1-based order of TSSs. If so, it would be relatively easy to show their correlation using an alternative sorting. However, they do not show any evidence for preferred co-occupancy of TAF9/10/12. They should either provide supporting analyses or remove the statements to avoid confusion.
• In Fig. S3, both dotted line and solid line are shown. The legend should indicate what they represent.
• In Fig. 2c, the authors appear to add an annotation "TAF3?" based on their prediction. Since this structure is used to validate that ChIP-nexus can support structural inference, it would be more appropriate to keep the original annotations from the source study. Also, the yeast module does not help here so it can be removed.
• The paragraph starting from Line 209 seems to be combined with the previous paragraph.
• Line 234 should cite relevant previous reports.

(Remarks on code availability)

Version 1:

Reviewer comments:

Reviewer #1

(Remarks to the Author)
I am satisfied by the authors' responses to my comments.

(Remarks on code availability)

Reviewer #2

(Remarks to the Author)
The authors did a good job addressing our comments and concerns. They added a few analyses and adjusted their wording throughout the manuscript. We think the paper has improved and do not have any remaining comments or concerns. We are in favor of publication

(Remarks on code availability)

Reviewer #3

(Remarks to the Author)
I appreciate the authors' effort to normalize the signals and present the TAF/TBP ratios, which clearly show that TAF occupancy is relatively lower at TATA promoters compared to DPR promoters at steady state. However, the interpretation of a low TAF/TBP ratio as a 'TAF-independent mechanism' remains conceptually flawed and potentially misleading. The reviewers 1 and 2 (points #6) also pointed out this concern.
ChIP-nexus captures steady-state residency, not assembly pathways or functional dependencies. A low TAF-to-TBP ratio simply implies that TAFs have a shorter residence time or higher turnover rate relative to TBP at TATA boxes. Earlier studies using acute degradation (degron) systems strongly argue against the authors' conclusion. For instance, acute Anchor-Away of Taf1 or Taf13 in yeast (Petrenko et al., eLife 2019) causes a global loss of TBP recruitment, even at strong TATA-containing promoters. Whiel the yeast system may not be fully comparable to the Drosophila cells, these functional studies suggest that TBP cannot efficiently engage TATA boxes in vivo without the assistance of TAFs (TFIID loading). If TBP requires TAFs to be loaded onto the promoter—even if TAFs subsequently disengage to allow TBP to facilitate rapid re-initiation during a burst—the mechanism is fundamentally TAF-dependent.
A good way to demonstrate this is to make a TAF1 degron cells and detect TBP occupancy. I don't think it is necessary to do so. However, the current statement and working model over-extrapolate steady-state binding data into functional genetics. To avoid misleading, I would suggest the author tone down or modify the conclusion of "TAF-independent", in the abstract, Figure 4, and working model.

(Remarks on code availability)

Reviewer #4

(Remarks to the Author)
I co-reviewed this manuscript with one of the reviewers who provided the listed reports. This is part of the Nature Communications initiative to facilitate training in peer review and to provide appropriate recognition for Early Career Researchers who co-review manuscripts.

(Remarks on code availability)

Reviewer #5

(Remarks to the Author)
The authors addressed most of our suggestions/concerns. The manuscript has improved substantially, and we would kindly request minor revisions in the following points before publication.

Line 133-134 should clarify that the classification is manual based on PCA. For example, “In agreement with PCA, we manually grouped …”

Line 217-218 “likely due to interactions with upstream transcription activators” is rather speculative. The authors should either provide further structural evidence to support this or remove this part.

Line 353 & 368, “less TAF-dependent” seems more appropriate than “TAF-independent” since TAF still binds these promoters.

Although most of the strong statements were toned down in the result section, they seems still present in the abstract. “Our results inform models for the increased burst size observed at TATA promoters” should be removed from abstract and kept in discussion since this statement remains correlative (lack of experimental validation including TBP/NC2/TAF perturbation).

(Remarks on code availability)

Open Access This Peer Review File is licensed under a Creative Commons Attribution 4.0 International License, which permits use, sharing, adaptation, distribution and reproduction in any medium or format, as long as you give appropriate credit to the original author(s) and the source, provide a link to the Creative Commons license, and indicate if changes were made.
In cases where reviewers are anonymous, credit should be given to 'Anonymous Referee' and the source.
The images or other third party material in this Peer Review File are included in the article’s Creative Commons license, unless indicated otherwise in a credit line to the material. If material is not included in the article’s Creative Commons license and your intended use is not permitted by statutory regulation or exceeds the permitted use, you will need to obtain permission directly from the copyright holder.
To view a copy of this license, visit https://creativecommons.org/licenses/by/4.0/

Overview

We were pleased that the reviewers appreciated the quality and resolution of the data and praised them as a valuable in vivo resource for understanding TFIID organization and promoter-specific transcription initiation. They also appreciated the agreement between our high-resolution footprints and published structural models, as well as the potential importance of the promoter-specific behavior of TBP, NC2, and TAFs. Meanwhile, the reviewers also questioned some details of our mechanistic interpretation of the data, especially with regard to promoter kinetics, which is far from what ChIP-nexus directly measures. This feedback was thoughtful and constructive, and prompted us to thoroughly revise the manuscript, while also adding additional analyses. We toned down our claims of TAF-independent initiation, more carefully interpreted the possible role of TBP at the TATA box in re-initiation, and explained in more detail what led us to interpret the downstream TBP signal as a TFIID loading state, acknowledging that alternative explanations are possible.

Additional analyses include:

- Using non-normalized TAF/TBP occupancy ratios, we show that TAF binding is consistently lower relative to TBP at TATA promoters compared with DPR promoters
- We reanalyzed the TBP occupancy using the upstream TBP signal, which reflects the engaged state. This revised analysis supports a more cautious model in which TBP may persist longer at the TATA box.
- We performed new analyses comparing promoters with high versus low NC2 occupancy while controlling for PIC and transcription levels, showing that elevated NC2 signal is associated with reduced TAF occupancy.
- We added control PCA and average footprint analyses to show that the overall spatial organization of TFIID subunits can also be observed without triptolide treatment.

We improved the clarity of the manuscript:

- We refined the structural interpretation of promoter-bound TFIID with more cautious wording, indicating that the footprints are broadly consistent with published cryo-EM structures and reveal promoter-bound conformational organization in vivo.
- We clarified that ChIP-nexus measures steady-state occupancy and cannot directly infer binding kinetics.
- We revised the interpretation of TBP binding at TATA promoters, acknowledging that our data cannot distinguish whether TBP binds the TATA box directly de novo or is first loaded by TFIID and then remains promoter-bound after TAF disengagement.
- We added a comparison between narrow and broad promoters to justify the use of narrow TSSs for footprinting analyses and to show how broader initiation patterns blur factor-positioning information.
- We added TFIIA and TFIIB as references and labeled structural lobes in Figure 2.
- We added PDB IDs structural references to the figure legends.
- We improved terminology to distinguish sequence-defined promoter classes from TBP-cluster-defined promoter classes in Figure 3.
- We corrected and clarified several figure and text issues raised by the reviewers, including strand-specific display in Figure 1C, TAF labeling and PCC values in the correlation matrix in Figure 1D, and the module assignments in Figure 2A,B.

Point-by-point response to reviewers

Reviewer #1 (Remarks to the Author):

In this paper the Zeitlinger lab uses their ChIP-NEXUS pipeline to analyze how TBP and TBP-associated factors (TAFs) crosslink to promoters in Drosophila KC167 cells. The distinct patterns match well with, and provide in vivo validation for, published structural models suggesting that different TFIID conformations exist. The crosslinking patterns correlate well with particular promoter sequences and behaviors. The data is clear and the interpretations are reasonable. Overall, this is a very nice paper that should be of wide interest to the gene expression community. I have some relatively minor comments and suggestions to consider before finalizing the paper for publication.

1. The figure legend for Fig 1C says both strands are shown in the same color. But only one heatmap is shown per TAF, which I assume is only one strand. Are the signals from the two strands are superimposed? It wouldn't make sense to show the data that way. Please clarify.

We thank the reviewer for noting this ambiguity. In Figure 1C, the original heatmaps of the ChIP-nexus signal were not strand-separated and instead pooled into a single intensity track with the same color.

To improve clarity and avoid confusion, we have revised Figure 1C to display signals from the positive and negative strands in different colors.

2. Fig 1D. I recommend adding the Correlation coefficients (currently shown in the supplemental figure) into the graph.

We thank the reviewer for the suggestion. We have now added the pairwise Pearson correlation coefficients (PCC) directly to Figure 1D.

3. Line 86. I think it overstates things to say that TFIID structure is inferred from crosslinking. The crosslinking is consistent with published structures, but I don't think you could produce the same model based on crosslinks alone. It would be more accurate to say the crosslinks reveal TFIID "conformation" rather than "structure".

We thank the reviewer for this important clarification. We agree that our data do not determine TFIID structure de novo, but rather reveal in vivo crosslinking patterns consistent with previously published structural models. We have replaced the title (line 116) to ***"High-resolution footprints reveal the promoter-bound conformation of TFIID in vivo".***

4. Line 140. The paper says that TBP primarily interacts with the phosphate backbone as an explanation for why there are no crosslinks up near the TATA box. This isn't accurate. TBP makes extensive hydrophobic interactions with bases in the minor groove, and two phenylalanines actually intercalate between TATA box bases. More likely, crosslinking is mostly mediated by base amines in the major groove, but not the minor groove where TBP is binding.

We thank the reviewer for this important correction. We agree that our original explanation was inaccurate and overstated the role of phosphate-backbone contacts. TBP makes extensive contacts with the TATA element through the minor groove, including intercalation of conserved phenylalanine residues between TATA bases. We have revised the text to clarify that the observed crosslinking pattern is likely influenced by the chemistry and geometry of formaldehyde crosslinking, consistent with preferential crosslinking to accessible reactive groups that are more exposed in the major groove than in the minor groove where TBP binds.

In the results (page 8, lines181-186) we now say: ***"Rather, the limited crosslinking efficiency may reflect a feature specific for how TBP contacts DNA. TBP interacts with DNA primarily through the minor groove, by inserting of conserved phenylalanine residues between TATA bases (Kim and Burley 1994), while formaldehyde crosslinking depends on the proximity and accessibility of reactive nucleophilic groups, which may be poorly positioned for efficient crosslinking when a protein contacts DNA mainly through a hydrophobic minor-groove interface (Hoffman et al. 2015). This may explain the weaker TBP footprint at the TATA box."***

5. Fig 2A: The figure legends don't mention the two black arrows pointing to peaks in the downstream and narrow upper traces. These should either be explained or removed.

We thank the reviewer for pointing this out. We have added the description for the arrows in the Figure 2C legend.

6. Fig 2A and B: It might be useful to include the crosslinking and PC patterns for TFIIA and TFIIB for comparison, since both are direct TBP interactors. These are shown in a Fig 4, where it's helpful for contrast to Mot1 and NC2. Also, in Fig 2C it could be helpful to label lobes A, B, and C in the figure, not just describe them in the legend.

We thank the reviewer for this suggestion. We have included TFIIA and TFIIB average profiles and PCA. TFIIA and TFIIB appear separated from the TAF modules, but closer to TBP in the PCA space, consistent with their upstream promoter contacts and association with TBP. We have also now labeled lobes A, B, and C directly in Figure 2C to improve clarity and to facilitate comparison with the description in the legend.

7. Fig 3B,C and Sup Fig S5C, D: These figures compare promoter classifications that are based on sequence motifs with those derived from clustering of the TBP crosslinking patterns. However, the same names (i.e. TATA, dpr, and hk) are used for both types of classifications, which is confusing. There's no reason to presume the two approaches would give identical classes, so using the same names hides the significance of the finding. It would be clearer if the two different classification methods were distinguished by using a subscript or other marker (maybe something like: "dpr-seq" versus "dpr-TBP").

We thank the reviewer for this insightful suggestion. We agree that using identical terminology for sequence-based and TBP-based classifications could imply equivalence and obscure the conceptual distinction between these two independent approaches. To clarify this, we have revised the figure and legend to explicitly distinguish the classifications.

Sequence-based promoter classes are now labeled with a "–seq" suffix (e.g., TATA-seq, DPR-seq, HK-seq), whereas TBP-based clusters are labeled with a "–TBP" suffix (e.g., TATA-TBP, DPR-TBP, HK-TBP).

8. Line 223 and 234. I don't follow the logic of the argument the authors present for concluding that TBP dissociates with each round of transcription. ChIP-NEXUS can measure steady-state occupancy, but not kinetics. Stronger TBP crosslinking could mean a longer dwell time per binding event, or more frequent binding events per unit time. In either case, I would expect a correlation with other GTFs and transcript output (CAGE).

We thank the reviewer for this important clarification. We agree that ChIP-nexus measures steady-state occupancy rather than binding kinetics, and therefore does not determine whether TBP dissociates after each initiation event. Our intention was to test whether TBP occupancy is higher than the PIC occupancy and transcription output specifically at TATA promoters. Assuming a certain amount of TBP per PIC per promoter, we tested whether TATA promoters exceed this amount. In our original analysis, we did not find significantly elevated levels of TBP, leading us to conclude that TBP dissociates and re-binds, just like at other promoters where TFIID loads TBP. However, reviewer 2 pointed out that it would be better to measure TBP upstream of the TSS, where the TBP signal should represent the engaged PIC. We agree and have now used this analysis in the revised version. We found that while TFIIA,TFIIB and TFIIF levels are still proportional to the transcription output across TATA and DPR promoters, TBP occupancy is higher at TATA promoters, suggesting that it may remain bound longer there.

The revised results text now states (page 13, lines 278-282): ***"For TBP, we analyzed the binding levels upstream of the TSS because the downstream signal was not associated with the PIC. This revealed that TBP binding was significantly higher at TATA promoters than at DPR promoters (Figure 4B). Thus, whereas other PIC components largely scaled with the transcription output, TBP showed elevated binding levels at TATA promoters. These observations suggest that while TFIIA, TFIIB and TFIIF do not remain bound across multiple rounds of transcription, TBP may persist longer at the TATA box, potentially facilitating rapid PIC reassembly during transcription reinitiation."***

We also now explicitly state (page 13, lines 272-273): ***"This ratio cannot infer initiation kinetics but provides an approximate measure of promoter occupancy relative to transcription initiation."***

9. Fig 4A. You can't really see what Mot1 is doing near the TATA box, as the signal is hidden behind NC2.

We thank the reviewer for pointing this out. To improve clarity, we have revised Figure 4A so that Mot1 is clearly visible in the TATA promoters.

10. Line 355, 362. The cited paper from the Cramer lab does claim TBP dissociates upon elongation, but the authors may want to make clear that this paper used purified GTFs with TBP. There is no activator, Mediator, or TAFs. Pointing out the absence of TAFs will better lead into their discussion a little later about TFIID loading TBP.

We thank the reviewer for this clarification, which contributed to our revised interpretation of our data. While we acknowledge the findings of the Cramer paper, we also note that recent live-cell single-molecule studies indicate that TBP exhibits longer promoter residence times than most Pol II and PIC components. Together with our revised analysis showing higher upstream TBP occupancy at TATA promoters relative to DPR promoters after normalization, we now extensively consider and discuss that prolonged binding of TBP may mediate multiple rounds of transcription (page 19).

11. The Discussion (line 359) says TATA promoters have “larger transcriptional bursts” than non-TATA promoters. I think this conflates burst size/amplitude with bursting behavior. My understanding is that non-TATA promoters don’t show any bursts. One model from yeast work (summarized in reference 10) is that TAFs remain bound through multiple rounds of transcription to keep nucleosomes from shutting off the promoter, while TBP (without TAFs) dissociates to create off-periods around the bursts. Bursting is defined by the “off” periods as much as the production of multiple transcripts during active periods.

We thank the reviewer for this important conceptual clarification. We have specifically focused here on explaining how TATA promoters generate “larger transcriptional bursts”, also known as burst size or burst amplitude. Other aspects of a promoter’s bursting behavior were not examined. In metazoans and specifically in Drosophila, transcription from both TATA and non-TATA promoters has been widely observed to occur in bursts, with promoter architecture primarily affecting burst size (Pimmett et al. 2021; Larsson et al. 2019; Tantale et al. 2016; Mahat et al. 2024; Lammers et al. 2020; Zoller et al. 2015; Luo et al. 2023). We have therefore revised the text to explicitly state that TATA promoters are associated with larger burst size (pages 2 line 54 and 19 line 402).

Reviewer #2 (Remarks to the Author):

In this manuscript, Alcantara et al. profile the genomic footprints of the 14 subunits of TFIID and several general transcription factors in Drosophila cells at high resolution. These results show concordance with structural models of TFIID binding to the core promoter and provide a complementary view of TFIID’s structural rearrangements. Their analysis reveals that TBP has the most promoter-specific binding patterns out of all TFIID subunits, to an extent that it can be used for promoter classification and to recover sequence motifs such as the TATA box. Profiling of TBP regulators revealed a specificity for the regulator NC2 for TATA promoters. They also provide evidence that, in cells, TBP can seed formation of the PIC in the absence of TAFs. Overall, the manuscript is well-written and appropriately referenced. The ChIP-nexus dataset is of high quality, complementary to prior structural studies, and could be a valuable resource to the field. The observation of TAF-independent transcription initiation in cells is a key result and provides valuable data into a mechanistic question that continues to be debated. However, we do have a few major concerns that must be addressed before we can support publication.

Major points:

1. The authors argue through the paper that their results agree very well with the available cryo-EM structures, but they seem to cherry pick from their data and from the information that can be deduced from the structure. For example, if one looks at the intensity displayed in Figure 1D, TAF4 and TAF12 are grouped together because they basically do not correlate strongly with anything else (the PCC between TAF4/TAF12 is also not that high). Both are known to form an integral part of two structural lobes of TFIID, so we are surprised they do not correlate strongly with anything else. We would expect them to correlate strongly with each other and with TFIIA and TBP, so why do they have such distinct behavior? We have the impression that the authors pick and choose what data fit with the structural work. Related to this, the discussion of Figure 1D with respect to the clustering and interpretation is confusing (see minor point 10).

We thank the reviewer for pointing out this discrepancy. Upon re-examining Figure 1D, we identified an error in the labeling of the TAFs in the correlation matrix. After correcting this error, TAF4 and TAF12 show the expected strong correlation with each other, as well as strong correlations with other TAFs and PIC components, in agreement with their established structural roles within TFIID.

We have replaced the figure with the corrected version in the revised manuscript. The correlation patterns are now more consistent with the structural organization of TFIID described in the cryo-EM studies. We apologize for this mistake and appreciate the reviewer's careful examination of the data.

2. One of the subsections is entitled "TFIID structure inferred from in vivo footprints". The authors really should tone this down. Maybe change it to something like "In vivo footprints are in agreement with TFIID structure" These data would not be able to define TFIID structure by itself.

We thank the reviewer for pointing this out. We agree that our data do not determine TFIID structure de novo, but rather reveal in vivo crosslinking patterns consistent with previously published structural models. We have revised the text 9 (line 116) and replaced the title to ***"High-resolution footprints reveal the promoter-bound conformation of TFIID in vivo".***

3. The question of whether TAF footprints differ at different promoters is not adequately explored. Although TBP does show the clearest differences in ChIP-nexus signal at different promoter types, there are differences among the other TAFs, which are only briefly acknowledged by the authors in lines 170-171. In Figure S6, not only do many TAFs have more diffuse footprints in TCT/HK promoters, but the peaks appear to shift (for example, TAF5 and TAF3 upstream regions). Related to this, while TAF footprints may be similar for TATA vs. DPR vs. TCT vs. HK promoters, the authors do not consider the possibility that partitioning promoters based on other criteria besides sequence motifs may reveal differences. For example, the authors suggest that TAF3's somewhat unexpected downstream footprint may arise in part due to its interaction with H3K4me3, so it would be interesting to evaluate whether its footprint differs at promoters with high or low H3K4me3.

We thank the reviewer for this insightful suggestion. While we do not note a major positional shift in the footprints, we agree that several TAFs display diffuse/broader footprints upstream of the TSS at TCT and HK promoters.

We have briefly expanded the results (page 9, lines 214-218) to state these differences more explicitly: ***"Comparing the profiles of all TFIID subunits at the four promoter types revealed that TBP has the most promoter-specific profile (Figure 3B), while most TAFs display broadly similar profiles across promoter classes, with only subtle differences. For example, the TAF binding profiles at TCT and HK promoters tend to be more diffuse with more pronounced upstream binding (Figure S6A), likely due to interactions with upstream transcription activators."***

We also considered the reviewer's suggestion whether TAF3 footprint patterns differ between promoters with high and low H3K4me3 enrichment. To test this directly, we stratified promoters by H3K4me3 levels and compared TAF3 ChIP-nexus footprints. We did this by multiple ways and did not observe a clear correlation between H3K4me3 enrichment and TAF3 occupancy or positioning. These results suggest that while H3K4me3 may facilitate TFIID recruitment, especially at promoters lacking canonical core promoter elements, it does not appear to substantially alter the DNA-bound position or levels of TAF3 within TFIID in our data.

This analysis has now been included in the results (pages 5 and 6, lines 145-148): ***"The presence of TAF3 in this module should allow the PHD finger of TAF3 to interact with the H3K4me3 mark of the +1 nucleosome downstream of the TSS[42,43], though we note that the levels of TAF3 (and TFIID in general) do not correlate with the levels of H3K4me3 (Figure S4C)."***

4. The observation that TBP has a downstream footprint, and that this footprint disappears after triptolide treatment, has previously been reported by the same research group in ref. 30 (Shao & Zeitlinger, Nat. Genet. 2017). However, in their discussion of this result (see lines 174-181, 321-325, 337), this paper is not cited. While we appreciate that this observation can now be better contextualized due to recent cryo-EM structures that were not available at the time of the earlier study, the authors should make it clear in the text which observations have been previously observed and thus are not fully novel.

We thank the reviewer for pointing this out. The downstream TBP footprint and its sensitivity to triptolide were indeed previously reported in our earlier study (Shao & Zeitlinger, Nat. Genet. 2017). In the current manuscript, we revisit this observation in the context of recent cryo-EM structures of the PIC and the expanded set of TFIID subunit footprints, which allow us to better interpret the structural basis of these signals. We have now added citations to the earlier study in the relevant sections of the manuscript and clarified that the downstream TBP footprint itself was previously observed.

5. The authors argue that since TBP does not show significantly higher normalized binding at TATA vs. DPR promoters (Figure 4B), it must not remain bound at the promoter through multiple cycles of initiation. However, this analysis seems to be flawed since it compares total ChIP-nexus signal in a window spanning the entire core promoter, which includes signal from non-engaged states (i.e., the +32 signal corresponding to a "loading" state). Instead, it would be more appropriate to compare ChIP-nexus signal around the -18 upstream region corresponding to engaged TBP, since if TBP were to remain engaged after TAF dissociation, one would expect it to remain bound at the TATA box. Figure 4A shows that binding specifically in the -18 region is higher for TATA vs. DPR promoters, so the authors cannot

conclusively rule out the possibility that TBP remains bound to the TATA sequence in TATA promoters after the other TAFs dissociate.

We thank the reviewer for this thoughtful comment. We agree that the TBP crosslinking downstream likely reflects a loading or non-engaged state and thus using only TBP binding upstream of the TSS is more appropriate. We have now repeated the analysis focusing only on TBP, TFIIA, TFIIB and TFIIF binding upstream of the TSS. The revised results now show that TBP occupancy is significantly higher at TATA promoters, while TFIIA,TFIIB and TFIIF remain proportional to transcriptional output between TATA and DPR promoters.

We have updated the text (page 13, lines 278-282) based on these new results: ***"For TBP, we analyzed the binding levels upstream of the TSS because the downstream signal was not associated with the PIC. This revealed that TBP binding was significantly higher at TATA promoters than at DPR promoters (Figure 4B). Thus, whereas other PIC components largely scaled with the transcription output, TBP showed elevated binding levels at TATA promoters. These observations suggest that while TFIIA, TFIIB and TFIIF do not remain bound across multiple rounds of transcription, TBP may persist longer at the TATA box, potentially facilitating rapid PIC reassembly during transcription reinitiation."***

6. The above point is still compatible with the authors' conclusion that the PIC can assemble around TBP in the absence of TAFs, which is based on their comparison of normalized TAF binding at TATA vs. DPR promoters. However, given the data in the manuscript, it is not possible to distinguish between a mechanism (1) where TBP is able to bind by itself to a TATA promoter de novo, versus (2) where TAFs initially load TBP and then dissociate so that future cycles of PIC formation use TBP that remains bound to the TATA sequence. The authors should carefully revise the text to acknowledge this ambiguity and avoid unqualified statements like "TBP can directly bind to the TATA box" (line 298), since their data do not directly support this claim.

We thank the reviewer for this important clarification. We agree that our data suggest that TBP can support transcription in the absence of TAFs, but they do not distinguish whether TBP finds the TATA box on its own (as it does in in vitro experiments) or whether TFIID initially loads TBP and subsequently dis-engages. We have revised the text and model to explicitly acknowledge both possibilities.

In the results section (page 16, lines 350-352) we now state: ***"At TATA-containing promoters, however, TBP could either directly bind to the TATA box and mediate PIC formation or promote TAF-independent transcription re-initiation after being initially loaded by TFIID."***

It now says in the discussion (page 19, lines 403-406): ***"By having alternative modes of transcription initiation, TATA promoters may allow more efficient reloading of TBP during a burst. TBP may either bind the TATA box directly to promote PIC formation, or TFIID could initially load TBP and subsequently dis-engage, while TBP remains promoter-bound."***

7. It would be interesting to look at whether TATA promoters with higher NC2 signal vs. lower NC2 signal have lower binding of PIC components, TBP, and/or TAFs. This analysis

should be straightforward to perform using the existing data, and could help strengthen the authors' conclusions regarding NC2.

We thank the reviewer for this helpful suggestion and have now performed this analysis. We selected promoters with the high and low NC2 while controlling for similar levels of TFIIA, TFIIB, TFIIF and CAGE-seq levels. This allowed us to assess whether NC2 enrichment is associated with differences in TBP or TAF occupancy independently of overall PIC abundance and transcription levels. As shown in the new Figure S8, promoters with high NC2 signal show significantly lower binding of all TAFs. These results support a model in which NC2 marks a specific promoter architecture (TATA promoters), which are characterized by reduced TFIID occupancy.

These results are now mentioned on page 15 (lines 321-325) of the results section: ***"Finally, we performed additional analyses to support the differences in binding patterns between TATA and DPR promoters. Because TATA promoters show strong NC2 binding and reduced TAF occupancy, we reasoned that with comparable PIC levels and transcription output, promoters with high NC2 should still be associated with higher TBP and lower TAF binding than promoters with low NC2, and this was indeed the case (Figure S8C-E)."***

Minor points:

8. It would be good if the authors can clarify at the first point in the text where CAGE-seq is mentioned that this is a publicly available dataset from modENCODE that has been reanalyzed, as opposed to new data collected in this study.

We thank the reviewer for this suggestion. We have now clarified in the text that the CAGE-seq data used in this study are from a previously published modENCODE dataset and were reanalyzed here.

This has been added at the first mention of CAGE-seq in the manuscript on page 3 (line 71): ***" We used CAGE-seq data from Kc167 cell nuclear extracts (publicly available from modENCODE: ENCSR827COJ) and selected promoters where the annotated TSS shows transcription initiation within 11 bp (n = 1,769; Figure S1; see Methods)."***

9. Concerning the analysis of the data, can the authors comment on what they mean when they refer to the "corresponding peak" in the other strand (line 97)? What does the difference between the position in the two strands say? Does it relate to the size of protein?

We thank the reviewer for this request for clarification. In ChIP-nexus, exonuclease digestion proceeds to the protein-DNA crosslink site, generating strand-specific peaks that typically appear as a pair: one on the positive strand and a corresponding peak on the negative strand downstream. By "corresponding peak," we refer to this paired signal arising from the same protein-DNA interaction reflected on opposite strands. We used the midpoint between the strand-specific peaks as an estimate of the primary DNA contact position.

We have revised the text on page 5 (lines 125-129) to: ***"Since ChIP-nexus produces strand-specific peaks on either side of the protein–DNA crosslink site, we calculated***

the midpoint between matching peaks on the positive strand and negative strand to identify the protein-DNA contacts in each average profile (Figure S3 left)."

10. In lines 76-77, the authors say "TBP had a higher correlation with TFIIA and other PIC components than with the TAFs (Figure 1D)." However, the individual PCCs for TBP and Pol II/TFIIF/TFIIB appear to be weaker than for TBP and some of the TAFs. It seems like the authors are referring instead to the fact that TBP clusters with TFIIA/Pol II/TFIIF/TFIIB based on all the pairwise PCCs. Similarly, in line 80, the authors refer to "the most correlated TAFs (TAF4/12, TAF1/7/2, TAF11/13 and TAF6/9)…" but the PCC for TAF4 and TAF12 is not very high. It would be good for the authors to use more precise wording when referring to the hierarchical clustering.

We thank the reviewer for pointing this out. We have revised the wording to clarify that TBP shows the highest correlation with TFIIA and clusters closer to PIC components based on the overall correlation matrix.

Regarding TAF4/12, upon re-examining Figure 1D, we identified an error in the labeling of the TAFs in the correlation matrix. After correcting this error, TAF4 and TAF12 show the expected strong correlation with each other, as well as strong correlations with other TAFs and PIC components, in agreement with their established structural roles within TFIID.

We have replaced the figure with the corrected version in the revised manuscript. We apologize for this mistake and appreciate the reviewer's careful examination of the data.

11. For any figures in which structural models are shown, the authors should at minimum cite the PDB IDs in the figure legend. This applies to Figures 1D, 2C, and S8B.

We thank the reviewer for this suggestion. We have now added the corresponding PDB accession codes for the structural models to the respective figure legends.

12. In Fig. 2B, TAF9 appears as an outlier in the PCA analysis shown, and so is TBP. It is not clear that the "upstream narrow" cluster is well-defined, as TAF9 could just as easily be considered part of the "upstream broad" cluster.

We thank the reviewer for pointing this out. Our assignment of modules was based not only on the PCA distribution but also on the similarity of the average ChIP-nexus footprint profiles. Factors in the "upstream narrow" module show their main footprint at the −14/−18 position. While TAF9 also exhibits a broad upstream footprint similar to that of the "upstream broad" module, its signal at the −14/−18 position is stronger. In contrast, factors in the "upstream broad" module display relatively higher signal in the broader upstream region compared to their −14/−18 footprint. Based on this distinction in footprinting profiles, we classified TAF9 (and TBP) within the "upstream narrow" group.

We have revised the text on page 5 (lines 133-134) to: ***"In agreement with Principal Component Analysis (PCA) of the average profiles, we grouped the 14 TFIID subunits into four modules, which we call the downstream, middle, upstream narrow and upstream broad module."***

We hope this clarifies that these groupings reflect overall trends based on both PCA positioning and footprint shape, rather than strictly discrete clusters.

13. Concerning the results in Fig. 2C, the authors state that “both the modular organization and the preferred DNA contacts align well with cryo-EM structures of promoter-bound human and yeast TFIID” (lines 101-102), but there are obvious exceptions. For example, TAF11 does not belong at all to the TAF6/8 module that is supposed to be in the middle. Therefore, the following statement, “demonstrating that our data can provide insights into the structural organization of the complex in vivo” (line 103) should be toned down.

We thank the reviewer for raising this point. We agree that the correspondence between our footprint-based organization and available cryo-EM structures overall agrees but is not exact. We have toned down relevant statements and now describe our data as being broadly consistent with structural models, rather than demonstrating the structural organization of TFIID in vivo. We do believe however that the surprising exceptions merit discussion since they could point to locations of TAFs that have not been captured in current cryo-EM structures, either because they are flexible or come from in vivo states not captured by purified components.

We have now revised the page 8 (lines 164-169) of the results section to more explicitly acknowledge the distinct behavior of TAF11: ***“More unexpectedly, TAF11 clusters within the middle module, even though TAF11 forms a heterodimer with TAF13, which is found in the upstream module[44]. TAF11/13 are known to interact with TBP and inhibit its binding to DNA in the canonical state of TFIID. Thus, in the conformation of TFIID where TBP engages the promoter, TAF11/13 must relocate or disengage from TBP[5,6]. One possible explanation is that the TAF11/TAF13 heterodimer adopts a flexible or repositioned configuration upon TBP engagement, allowing TAF11 to shift closer to the middle module while TAF13 remains positioned upstream.”***

We have also reviewed our concluding sentence for this section of the results in page 9 (lines 195-197): ***“Taken together, the ChIP-nexus profiles show strong overall agreement with the in vitro cryo-EM structures, provide insights for less well-characterized subunits such as TAF3, and point to unexplained observations for TAF11/13 and TBP.”***

14. Can the authors comment on why TAF13 does not follow the same pattern as TAF11?

We thank the reviewer for this follow-up question. Although TAF11 and TAF13 form a heterodimer in structural studies, ChIP-nexus footprints primarily reflect proximity to DNA rather than protein–protein interactions within the complex. Thus, the two subunits may produce distinct footprint patterns if the heterodimer adopts a flexible or repositioned configuration during promoter engagement. As mentioned above, one possible explanation is that TBP engagement with the TATA region requires disruption or rearrangement of the inhibitory interaction between TBP and the TAF11/TAF13 module. This could allow TAF11 to shift closer to the middle module, while TAF13 remains positioned further upstream.

15. The authors state, “TBP primarily interacts with the DNA phosphate backbone, rather than the bases, which are the preferred substrate of formaldehyde crosslinking” (lines 140-

141). This is a strange argument, as TBP is one of the TFIID subunits that actually recognizes sequences. The argument could then be applied to most of the TAFs.

We thank the reviewer for this correction. We agree that TBP makes extensive base-specific contacts, including intercalation of conserved phenylalanines within the TATA element, and our original wording overstated the role of phosphate-backbone contacts in explaining the reduced signal at the TATA box. We have revised the text to clarify that the observed crosslinking pattern is likely influenced by the chemistry and geometry of formaldehyde-mediated crosslinking, consistent with preferential crosslinking to accessible reactive groups that are more exposed in the major groove than in the minor groove where TBP binds.

In the results (page 8, lines 180-186) we now say: ***"Rather, the limited crosslinking efficiency may reflect a feature specific for how TBP contacts DNA. TBP interacts with DNA primarily through the minor groove, by inserting of conserved phenylalanine residues between TATA bases (Kim and Burley 1994), while formaldehyde crosslinking depends on the proximity and accessibility of reactive nucleophilic groups, which may be poorly positioned for efficient crosslinking when a protein contacts DNA mainly through a hydrophobic minor-groove interface (Hoffman et al. 2015). This may explain the weaker TBP footprint at the TATA box."***

16. The observation that TAF4 has a broad footprint in the upstream proximal promoter (discussed in lines 145-150) is a really nice finding that complements prior biochemical findings. TAF4 seems to be a major hotspot for interactions with transcriptional activators, possibly more than other TAFs. We think it would be good to emphasize this more in the discussion of this finding. Consider also citing the following relevant papers: PMIDs 17483474 and 23873942.

We thank the reviewer for highlighting this point. We agree that the broad upstream footprint of TAF4 is an interesting observation and may reflect its role as a major interface for transcriptional activators. We have expanded our results to emphasize that TAF4 has been identified as a prominent interaction hub for activators in previous biochemical studies and incorporated the suggested references (PMID: 17483474 and 23873942).

It now says on page 9 (lines 191-193): ***"Specifically, TAF4 has been identified as an interaction hub for transcription activators in biochemical studies (Chen et al. 2013; Wang et al. 2007), supporting the idea that broad upstream footprints may reflect activator-mediated stabilization of TFIID".***

17. The authors do not comment on the fact that TAF12, and especially TAF4, also have a peak at around -18/-14. Interestingly, this is a site where conserved positive residues in TAF4 contact the DNA in the structure.

We thank the reviewer for pointing out this interesting observation. We agree that TAF4 and TAF12 peak around −18/−14 coincides with DNA contacts in cryo-EM structures. However, we note that this position represents a broader crosslinking hotspot in our data, as footprints from all TAFs as well as TBP and PIC components are observed at this location, especially after triptolide treatment. We have now added a brief discussion of this point in the Results,

noting that the −18/−14 footprints likely reflect the combined proximity of several PIC components at this position.

We say on page 8 (lines 172-176): ***"This position seems to be a broader crosslinking hotspot in our data, since all TFIID subunits, as well as TFIIA and TFIIB, show a footprint at this location. This likely reflects the dense network of interactions within the engaged PIC at this region."***

18. In Figure 2B,C, the downstream TAF cluster consists of those TAFs unique to lobe C (TAF2/1/7), along with TAF3. This is surprising, since TAF3 is known to move with TAF5,10,12,4,6,9,11,13 in lobe A to be everywhere, including upstream when in the process of engaging upstream DNA. Could the authors comment further on this?

We thank the reviewer for raising this point. We agree that clustering of TAF3 with the downstream TAF module in our analysis was initially unexpected. However, available structural models of promoter-bound TFIID suggest that lobe A is repositioned between lobes B and C, likely contacting the H3K4me3-marked nucleosome downstream of the TSS (see PMCID: PMC7156316). Thus, although TAF3 is part of lobe A, its DNA contacts in the engaged complex may not necessarily be restricted to the upstream promoter region.

Our observations are also consistent with crosslinking mass spectrometry studies in yeast, which revealed a dense network of interactions between TAF3 and TAF1–TAF2–TAF7, higher than with other TAFs. Together, these results may explain why TAF3 ChIP-nexus footprints cluster more closely with the downstream TAFs despite being structurally associated with lobe A.

We have modified this section in the results section (page 5 and 6, lines 143-148) to make this point more clear: ***"Interestingly, TAF3 is also included in our downstream module. TAF3 is part of the flexible lobe A, whose location in promoter-bound TFIID is not well established5,6, but the proximity of TAF3 near TAF1/2/7 is supported by crosslinking mass spectrometry analysis in yeast4. The presence of TAF3 in this module should allow the PHD finger of TAF3 to interact with the H3K4me3 mark of the +1 nucleosome downstream of the TSS9,43,44, though we note that the levels of TAF3 (and TFIID in general) do not correlate well with the levels of H3K4me3 (Figure S4C)."***

19. The Figure 2C legend states that these structures are crystal structures, but in fact they are cryo-EM structures.

We thank the reviewer for pointing out this error. The structures shown in Fig. 2C are indeed derived from cryo-EM studies rather than crystal structures. We have corrected the figure legend accordingly.

20. In Figure 3D, the color key for the negative strand should be fixed so that it is blue instead of gray.

We thank the reviewer for pointing this out. The color key for the negative strand in Fig. 3D has now been corrected to match the blue color used in the figure.

21. In the reporting summary, the authors state under the Data Exclusions section that “Only experiments that worked were selected as samples.” Could the authors clarify what they mean here?

We apologize for the unclear wording. We have revised the Data Exclusions section to state that failed ChIP-nexus libraries were excluded based on standard quality-control criteria, including insufficient DNA yield, excessive adapter contamination, or high duplicate rates after sequencing. For each factor analyzed, at least two successful biological replicates were generated and showed reproducible signals, which were used for the main analyses.

Reviewer #3 (Remarks to the Author):

In this manuscript, Alcantara et al. present a comprehensive, high-resolution binding atlas of the complete TFIID complex and associated factors (TBP, NC2, Mot1, TRF2) in Drosophila cells using ChIP-nexus. By generating base-pair resolution footprints, the authors validate that the in vivo modular organization of TFIID aligns remarkably well with recent cryo-EM structures and reveal distinct promoter-specific binding modes: a downstream loading state prevalent at DPR promoters and a specific upstream TBP engagement at TATA promoters facilitated by NC2. This work provides valuable in vivo evidence for TFIID footprints and proposes a dual mechanism of transcription initiation—TAF-dependent loading versus TAF-independent direct TBP binding—offering a potential mechanistic explanation for the distinct transcriptional bursting properties observed at TATA-containing genes. While the data quality is exceptional and the study addresses long-standing questions regarding TFIID function, the interpretation of these results requires more caution before publication.

Major Concerns
1. TAF-independent initiation and dynamic turnover:
The authors conclude that TATA promoters utilize a TAF-independent initiation mechanism, primarily because TAF occupancy (normalized to CAGE-seq transcript levels) is lower at TATA promoters compared to DPR promoters (Figure 4). While the validity of the observed lower occupancy is not in question, the interpretation of this result as independence is potentially misleading.
Transcription initiation is a dynamic cycle involving the association and dissociation of factors, whereas ChIP-nexus provides a static snapshot of steady-state occupancy. A lower steady-state signal at TATA promoters does not necessarily imply that TAFs are not required or not present during the initiation event; it could alternatively indicate that the PIC is highly dynamic with a rapid turnover rate (high dissociation/re-initiation frequency). In this scenario, TAFs might be essential but their residence time is short, resulting in a lower captured signal compared to the more stable complexes at DPR promoters.
Therefore, the distinction between TAF-dependent and TAF-independent might be better explained by kinetic differences rather than a fundamental structural binary. The authors should discuss these dynamic possibilities (association/dissociation kinetics) and avoid presenting the TAF-independent model as an absolute dichotomy to avoid misleading readers regarding the underlying mechanism.

We thank the reviewer for this thoughtful comment. We agree that ChIP-nexus measures steady-state occupancy and cannot directly distinguish factor residence time or turnover. Thus, reduced TAF occupancy at TATA promoters should not be interpreted as evidence that TAFs are not at all required for initiation.

While we cannot infer kinetics from ChIP-nexus data, our results argue against a more short-lived PIC at TATA promoters. This is because TBP, TFIIA, TFIIB and TFIIF levels are higher or similar at TATA and DPR promoters, which is not consistent with a uniformly increased turnover of the entire PIC at TATA promoters. The simplest interpretation for the low TAF occupancy is therefore that some PICs are formed by TBP in the absence of TAFs. It is entirely possible though that the first round of transcription occurs though TFIID loading TBP onto the TATA box, in which case the TAFs could still be necessary to initiate a burst of transcription.

We have now revised the manuscript to present a more cautious interpretation. On page 17 (lines 366-368) of the discussion, we now say: ***"These findings are consistent with TATA promoters producing a substantial fraction of transcription in a TAF-independent manner…".*** *Also, on page 19 (lines 403-406) we say:* ***"By having alternative modes of transcription initiation, TATA promoters may allow more efficient reloading of TBP during a burst. TBP may either bind the TATA box directly to promote PIC formation, or TFIID could initially load TBP and subsequently dis-engage, while TBP remains promoter-bound."***

We also explicitly acknowledge on page 13 (lines 272-273) of the results section that we cannot infer kinetics: ***"This ratio cannot infer initiation kinetics but provides an approximate measure of promoter occupancy relative to transcription initiation."***

2. The TBP footprint location:
The authors observe the strongest TBP footprint at -18 bp rather than the TATA box (-30 bp), attributing the lack of signal at -30 bp to poor crosslinking of TBP to the DNA phosphate backbone. However, they simultaneously show a sharp NC2 footprint at -33 bp. If NC2 binds the TBP-DNA complex as described in the structural model, it is puzzling why the NC2 signal captures the -30 bp region while TBP does not. This discrepancy raises questions about what the -18 bp footprint actually represents.
The authors suggest the -18 bp signal might come from interactions with other PIC components (e.g., TFIIB). If the TBP signal is indeed indirect, this complicates the proposed model of "direct TBP binding" (Figure 5). The authors should reconcile the presence of the NC2 footprint at the TATA box with the absence of a TBP footprint at the same location to substantiate their structural conclusions.

We thank the reviewer for raising this important point. Indeed, the TBP footprint at -18 bp has puzzled us for a very long time and caused us to consider many hypotheses where TBP might not bind at the TATA box. However, no such alternative hypothesis was viable in the end and made it into the manuscript. In the end, we felt that the NC2 footprint at the TATA box actually supports the presence of a TBP–DNA complex, since NC2 binds TBP-bound DNA. TBP just may not efficiently cross-linked there. This interpretation is supported by the TBP–NC2–DNA structure. TBP recognizes the TATA box mainly through minor-groove binding and base intercalation, which may crosslink inefficiently. In contrast, NC2α/β

contains lysine residues positioned near the TATA region in a geometry more favorable for crosslinking, likely explaining the sharp NC2 footprint around −33 bp. (see results, page 15, lines 316-320)

We have now revised the text in the results (page 8, lines 171-186) to say: ***"Notably, the strongest footprint of TBP is found at the -18 bp position. While this is consistent with previous Drosophila TBP ChIP-nexus data and human TBP ChIP-exo data33,38,39, it is puzzling since the TATA box is found at -30 bp, and according to cryo-EM structures6, TBP binds a similar position at TATA-less promoters. This is unlikely due to a general lack of crosslinking at this position since TAF13 has a strong footprint there (Figure 2C, arrow). Rather, the limited crosslinking efficiency may reflect a feature specific for how TBP contacts DNA. TBP interacts with DNA primarily through the minor groove, by inserting of conserved phenylalanine residues between TATA bases46, while formaldehyde crosslinking depends on the proximity and accessibility of reactive nucleophilic groups, which may be poorly positioned for efficient crosslinking when a protein contacts DNA mainly through a hydrophobic minor-groove interface37. This may explain the weaker TBP footprint at the TATA box."***

In the discussion (page 18, lines 382-286) we now say: ***"In contrast, NC2 showed strong, sharp ChIP-nexus footprints specifically at TATA promoters, but not at TATA-less promoters. These footprints agree with structural data of the TATA-TBP-NC ternary complex (Kamada et al. 2001) and provide indirect evidence that TBP strongly binds at the TATA box, although its strongest footprint is detected slightly more downstream at -18 bp."***

3. The loading state and the effect of Triptolide:
The identification of the downstream module (+10 to +32 bp) as a "TFIID loading state" relies on its disappearance upon Triptolide treatment. Since Triptolide inhibits TFIIH and causes Pol II arrest shortly after initiation, it is crucial to clarify whether the disappearance of the downstream TBP/TAF signal is due to a genuine conformational change (transition to the upstream state) or simply a steric effect where the arrested Pol II occludes the downstream binding site.

We thank the reviewer for raising this important point. We believe that both the steric occlusion and the alternative state of TFIID are true. The loss of the downstream TBP signal is likely because TFIID is preferentially in an engaged PIC state after triptolide treatment (together with arrested Pol II) and thus sterically occludes other versions of TFIID such as the loading state. We acknowledge however that our data do not show a conformational change of TFIID; this is simply our preferred explanation given all evidence. We have now worded this more carefully in the revised version.

In the discussion (page 18 and 19, lines 393-401) we now say: ***"While we cannot exclude alternative explanations, several observations point to downstream TBP being part of the DNA-bound loading or reloading state of TFIID. Downstream TBP is not observed after triptolide treatment, when the DNA-binding contacts suggest that TFIID is preferentially in a PIC state and thus would sterically hinder other conformations of***

TFIID. Downstream TBP is also reduced at promoters with relatively stable Pol II pausing, either due to sterical occlusion as well or because these promoters are less active. Given that cryo-EM structures suggest that the downstream position corresponds to the site where TFIID initially binds DNA to place TBP upstream of the promoter[5], we favor the hypothesis that downstream TBP reflects the DNA-bound loading state of TFIID."

4. NC2 and Transcriptional Bursting:
The authors propose a model where NC2 and the specific TBP binding mode contribute to the large burst sizes observed at TATA promoters. While this is a compelling hypothesis, the current link is largely correlative. The manuscript would benefit from providing more evidence or a deeper analytical connection between NC2 binding strength and bursting parameters to move this claim beyond speculation.

We thank the reviewer for this helpful comment. We agree that our current data do not directly establish a causal relationship between NC2 binding and transcriptional bursting. Our intention was to propose a mechanistic hypothesis that links the strong NC2 association, the distinctive TBP footprint at TATA promoters and the large burst sizes previously reported for this promoter class.

To strengthen this point analytically, we performed an orthologous analysis approach. We compared promoters with high and low NC2 signal, while controlling for similar CAGE-seq, TFIIA, TFIIB, and TFIIF levels. This allowed us to assess NC2 enrichment independently of overall PIC and transcription levels. We found that high-NC2 promoters show significantly reduced TAF occupancy and are enriched for the TATA element. They support a model in which NC2 marks a TATA-associated promoter architecture characterized by strong TBP/NC2 occupancy and reduced TFIID engagement. Since TATA promoters have previously been linked to larger burst sizes, our findings provide a correlative connection between NC2 binding and promoter features associated with bursting.

These results are now mentioned on page 15 (lines 321-325) of the results section: ***"Finally, we performed additional analyses to support the differences in binding patterns between TATA and DPR promoters. Because TATA promoters show strong NC2 binding and reduced TAF occupancy, we reasoned that with comparable PIC levels and transcription output, promoters with high NC2 should still be associated with higher TBP and lower TAF binding than promoters with low NC2, and this was indeed the case (Figure S8C-E)."***

Reviewer #4 (Remarks to the Author):

I co-reviewed this manuscript with one of the reviewers who provided the listed reports. This is part of the Nature Communications initiative to facilitate training in peer review and to provide appropriate recognition for Early Career Researchers who co-review manuscripts.

Reviewer #5 (Remarks to the Author):

In this study, Alcantara et al. define a comprehensive binding map of transcription initiation complexes in Drosophila using ChIP-nexus. This work reports high-quality, spatially resolved datasets for TFIID subunits that will clearly be valuable to the field. Their attempt to infer structural configuration from ChIP-nexus footprints is an ambitious and exciting application of the method at the interface of structural biology and genomics. However, the manuscript requires substantial revision, including reanalysis and reconsideration of several interpretations, before it seems suitable for publication.

Selection of narrow TSSs
The authors define "narrow promoters" based on CAGE-defined TSS width and focus most analyses on this subset, which accounts for roughly one-fourth of all active promoters. Although this filtering could significantly affect the results, the authors do not clarify the motivation for this selection nor do they evaluate the effects caused by this selection. For example, narrow TSSs (<10bp) are known to be enriched for containing a TATA-box, which could bias their analyses on subunit positioning.

1. Rationales for the narrow TSS selection
The authors should explain why they restrict the analyses to narrow TSSs. If the concern is that including all promoters would blur the signal and complicate spatial inference, they should show what the signal across all active promoters looks like and demonstrate why this filtering is required and how it is justified.

We thank the reviewer for this important point. Our analyses focus on narrow TSSs because ChIP-nexus provides high spatial resolution relative to the annotated TSS, and this positional information is most interpretable when initiation occurs at a well-defined site. At broad promoters, transcription initiates over a wider window, so aligning profiles to a single representative TSS can blur strand-specific peaks and reduce the ability to infer precise factor-DNA contact positions.

We now show the average binding footprints across narrow and broad promoters side by side in the new Figure S3. As expected, broad promoters show substantially more diffuse profiles than narrow promoters. We hope this comparison illustrates why we used narrow promoters for the footprinting analyses.

We have revised the text (page 3, lines 69-70) to clarify this rationale: ***"We then chose active narrow promoters for our analysis since the positional information of high-resolution ChIP-nexus footprints is most interpretable when initiation occurs at a well-defined site."***

In page 5 (lines 124-125) we also say: ***"As expected, promoters with broad transcription initiation exhibited substantially more diffuse average profiles (Figure S3, right)."***

2. Confounding effects from selecting narrow TSSs

It is unclear whether the module positions for each subunit (Fig. 2) are conserved across promoter classes or are specific to TATA-box promoters. For example, if TATA-box promoters are excluded, do the profiles remain similar? If the Fig.2 results are specific to TATA-box promoters, the figure and text should be reframed explicitly as describing configuration at TATA-box promoters. In addition, Fig. 2C should clarify whether the cryo-EM structure shown is derived from a TATA-box promoter or a TATA-less promoter.

We thank the reviewer for this important point. Figure 2 was generated from the full set of narrow promoters and was not intended to represent TATA promoters specifically. TATA promoters (n=191) comprise only ~11% of the narrow-promoter set (n=1769), which is also enriched for other core promoter motifs, including Inr, MTE, DPE, PB, and TCT (updated Figure S1C).

We have clarified that Figure 2 represents the aggregate TFIID/PIC organization across narrow promoters, while promoter-class-specific differences are analyzed separately in Figure 3. We revised the text (page 5, lines 128-129) to avoid implying that this configuration applies identically to all promoter classes: ***"We note that the DNA-bound organization of TFIID analyzed here reflects an aggregate pattern across diverse promoter classes (Figure S1C)."***

Finally, we updated the Figure 2C legend to state that the cryo-EM structure was derived from the synthetic Super Core Promoter template, which contains TATA, Inr, MTE, and DPE elements. The structure is similar for other promoter types and is therefore used as a general reference framework, not as a promoter-class-specific model.

Structural inference from ChIP-nexus data.
This section is particularly interesting because the authors propose that ChIP-nexus data could be used to predict structural relationships between components. However, to support this approach, it requires further clarification and validation.

1. Effects of triptolide treatment
The authors use triptolide treatment to enhance ChIP enrichment. Although a limited comparison between DMSO and triptolide conditions is shown (e.g. Fig. S6), it remains unclear how strongly the treatment alters footprint profiles. Notably, Fig. 2 appears to rely only on the triptolide condition, but the conclusions would be more convincing if the authors showed that the same overall spatial relationships hold without treatment. It would be useful to provide a PCA plot and average binding profiles (same as Fig. 2B and Fig. S4, respectively) using non-treated condition.

We thank the reviewer for this suggestion. We agree that it is important to demonstrate that the spatial relationships described in Figure 2 are not specific to triptolide treatment. We now include the PCA and average binding profile analysis using the untreated (DMSO) condition. As shown in the new Figure S4, the overall positional relationships among the TAF modules are not identical but remain similar, indicating that they are not dependent on triptolide treatment. Notably, TBP occupies an intermediate PCA position between the "upstream narrow" and "downstream" modules in DMSO due to its downstream signal, which is not present under triptolide treatment.

We have added this analysis in the new Figure S4 and updated the results (page 5, lines 136-138) accordingly: ***"In the untreated (DMSO) condition, similar TFIID modules were observed (Figure S4A,B), indicating that they are not specific for triptolide treatment."***

2. Module classification

The authors use PCA to characterize spatial profiles of TFIID subunits, but it was not clear to this reviewer from the Methods section how they group these factors into four modules. Is it manually grouped based on the PCA plot or is a clustering method used? In addition, the average positions of fragment midpoints (or an equivalent quantitative positional summary) should be shown to assess whether the footprint positions correlate with the structural configuration.

We thank the reviewer for pointing this out. The PCA analysis was done directly on the average signal profiles and used primarily as a visualization of similarities among ChIP-nexus footprint profiles. Module assignment was based on the PCA clustering space as well as the similarity of average binding profiles. We have revised the text and methods section to clarify that these groupings reflect overall trends based on both PCA positioning and average footprint shape, rather than strictly discrete clusters.

In the results (page 5, lines 133-134) we now say: ***"In agreement with Principal Component Analysis (PCA) of the average profiles, we grouped the 14 TFIID subunits into four modules, which we call the downstream, middle, upstream narrow and upstream broad module (Figure 2A,B)."***

In the methods (page 31) we explicitly state: ***"Modules were defined based on PCA clustering and similarity in the average binding profiles."***

Downstream TBP binding

The authors emphasize a TBP binding peak downstream of TSS and interpret it in the context of a TFIID loading state model. However, because ChIP-nexus signal can be highly influenced by crosslinking to nearby proteins (as the authors note), it is not clear that this downstream peak truly reflects loaded TBP.

Based on the observation that triptolide diminishes the downstream TBP peak (Fig. S6), the authors link this peak with TFIID loading state. However, other TAF subunits also seem to show reduced downstream signal (TAF4/13/2/1/3 in Fig. S6). Given that the TBP crosslinking can be influenced by other proteins in the proximity, further evidence is required to support the conclusion that reduced downstream TBP signal specifically indicates loss of TBP loading by TAFs.

We thank the reviewer for raising this important point. We agree that ChIP-nexus signal should not be interpreted as a direct physical map of TBP binding since crosslinking can be influenced by nearby proteins.

We would first like to clarify a potential source of confusion in the original Figure S6. DMSO and triptolide profiles were plotted on different y-axis scales. When shown on a common scale, the apparent reduction in downstream signal for several TAFs is no longer observed. Instead, their downstream signal is maintained or increased after triptolide treatment. We have revised Figure S6 so that both conditions are displayed using the same scale.

We also agree that triptolide sensitivity alone does not prove that the downstream TBP peak reflects TFIID-mediated loading. Instead, we discuss our reasoning more carefully in the discussion (page 16, lines 390-401) of the revised manuscript:

"A more puzzling observation is that under steady-state conditions, all promoters with TFIID show TBP binding downstream of the TSS. This downstream binding is most strongly associated with TAF2 at DPR promoters and is relatively lower at TATA promoters, where the TAF occupancy is also lower, suggesting that downstream TBP it is part of TFIID. While we cannot exclude alternative explanations, several observations point to downstream TBP being part of the DNA-bound loading or reloading state of TFIID. Downstream TBP is not observed after triptolide treatment, when the DNA-binding contacts suggest that TFIID is preferentially in a PIC state and thus would sterically hinder other conformations of TFIID. Downstream TBP is also reduced at promoters with relatively stable Pol II pausing, either due to sterical occlusion as well or because these promoters are less active. Given that cryo-EM structures suggest that the downstream position corresponds to the site where TFIID initially binds DNA to place TBP upstream of the promoter, we favor the hypothesis that downstream TBP reflects the DNA-bound loading state of TFIID.

Normalization signal with CAGE reads
The authors normalize the ChIP-nexus signal at individual promoters by transcript levels measured by CAGE. CAGE detects transcript start site from total RNAs (mostly cytoplasmic) and does not directly reflect the rate of transcription initiation events per promoter. CAGE signal is influenced not only by initiation but also by pausing, elongation, RNA half-life, etc. Moreover, transcription output is a consequence of subunit binding, so it does not seem intuitive to divide binding strength by CAGE mRNA expression. It would be clearer if the authors provide the similar analyses by binning promoters by expression levels (to control the transcriptional activity) but still using non-normalized binding values.

We thank the reviewer for this thoughtful comment. In this study, the CAGE-seq data used were generated from nuclear RNA, which more closely reflects promoter-proximal transcriptional output than total or cytoplasmic RNA. Nevertheless, we agree that CAGE signal is not a direct measure of initiation rate and may be influenced by pausing, early elongation, processing, or RNA stability. We have revised the text to state that CAGE normalization provides an approximate measure of steady-state factor occupancy relative to transcriptional output, not binding per initiation event or initiation kinetics. We now say (page 13, lines 272-273): ***"This ratio cannot infer initiation kinetics but provides an approximate measure of promoter occupancy relative to transcription initiation.".***

The reviewer's suggestion of binning promoters by expression levels is a good idea, but is not straightforward in this case because TATA and DPR promoters have different expression distributions, and TATA promoters are relatively few. Strict binning or matching by expression would compare small, non-representative subsets, potentially removing genuine promoter-class differences linked to transcriptional output.

To provide an alternative to our normalization approach, we have compared the non-normalized TBP and TAF occupancy at TATA and DPR promoters, by calculating TAF/TBP

occupancy ratios across promoters. This should address the reviewer's concern since it avoids transcript normalization while preserving the full promoter-class comparison. The results show that TATA promoters have consistently lower TAF occupancy relative to TBP than DPR promoters, supporting reduced TAF engagement independently of CAGE normalization.

We have included this analysis in the updated Figures 4C and 6B and modified the text in the results (page 15, lines 305-307) accordingly: ***"To make sure that this conclusion was not dependent on the normalization, we confirmed that the expected relative ratios of TBP and TAF binding were also present in the non-normalized data (Figures 4C; S6B)."***

"TAF-dependent" versus "TAF-independent" mechanisms – lack of validation
The authors describe "TAF-independent" initiation at TATA-box promoters (Line 264-267). Although CAGE-normalized TAF binding is reduced at these promoters, absolute TAF signal is still high and even at comparable levels for several TAFs (e.g. 1/2/3/4/13) (Fig. 3B, Fig. S6). Thus, the current correlative analyses do not support a conclusion of "TAF independence." Without experimental validation (e.g., TAF perturbation), the claim should be removed /toned down and the term "TAF-independent" not used. Also, the reduction in TAF-binding at TATA-box promoters can be strongly observed particularly in normalized values potentially due to the high CAGE signal at TATA-box promoters (worth checking it).
The authors propose that two distinct mechanisms at TATA-box promoters, TAF-dependent and TAF-independent PIC formation (Line 275-279), and highlight the resulting model summary in Fig.5. Given the limited experimental evidence, this conclusion is too strongly stated. To properly demonstrate two distinct modes, they would need evidence that can distinguish different states at the same promoter such as single-molecule measurements. Otherwise, they should point to this possibility not in the result but speculatively in the discussion and schematic model needs to be removed.

We thank the reviewer for this thoughtful comment. We agree that our data demonstrate reduced steady-state occupancy of TAFs at TATA-box promoters relative to TBP and transcriptional output, but do not establish that initiation occurs in the complete absence of TAFs. We note that TAF perturbation experiments have been performed in the past and have shown that TATA promoters are less dependent on TAFs, but we agree that our mechanistic interpretation was over-simplified. We have now softened our TAF-independent interpretation and now describe that TATA promoters show reduced TAF occupancy, consistent with a fraction of transcription occurring in the absence of TAFs.

In the results section (page 15, lines 307-309) we now say: ***"These data show that TATA promoters have substantially lower occupancy of TAFs and support the hypothesis that to some extent, TBP can initiate PIC formation from the TATA box without TFIID in vivo."***

Also, in page 16 (lines 331-334) of the results we now say: ***"Taken together, these results support a model in which TATA promoters do not initiate all rounds of transcription through TFIID. Instead, relatively stable TBP binding at the TATA box may enable PIC formation independently of TFIID, a mechanism that may be antagonized by NC2."*** *and conclude:* ***"At TATA-containing promoters, however, TBP could either directly bind to***

the TATA box and mediate PIC formation or promote TAF-independent transcription re-initiation after being initially loaded by TFIID."

In the discussion (page 17, lines 366-368) we now say: ***"These findings are consistent with TATA promoters producing a substantial fraction of transcription in a TAF-independent manner…"*** *and in page 18:* ***"Because prior studies used a subset of TAFs as proxies for the entire TFIID complex, our high-resolution in vivo analysis of the complete set of TAFs consolidates the finding that PIC formation at TATA promoters occurs to some extent in the absence of TFIID."***

Minor comments:

• Several structural models are shown in the figures; the legends should cite the studies from which each structure was obtained.

We thank the reviewer for this helpful suggestion. We have revised the figure legends to include citations to the studies from which the structural models were obtained. These references are now provided together with the corresponding PDB accession codes in the relevant figure legends.

• Line 32: "transcriptional initiation" is used, while in other parts, "transcription initiation". Please use one term consistently.

We thank the reviewer for pointing this out. We have revised the manuscript to use "transcription initiation" consistently throughout the text.

• Line 62 should refer to the results obtained in this study, not only references.

We thank the reviewer for pointing this out. We have revised the sentence in line 62 to refer explicitly to the results obtained in this study in addition to the relevant previous studies.

• In Fig. 1C, promoters are sorted by TAF1 signal. To represent the signal without being biased to one sample, it would be fair to sort the protomers based on CAGE tpm or an independent metrics.

We thank the reviewer for this helpful comment. In the original Figure 1C, promoters were sorted by TAF1 signal because the purpose of the analysis was to assess whether binding levels for the different TAF subunits overall correlate across promoters. Since TAF1 is a subunit with strong promoter-associated signal in our dataset, sorting by TAF1 provided a useful way to visualize whether other TAFs show coordinated promoter occupancy consistent with shared TFIID recruitment.

However, we understand that sorting by one ChIP-nexus dataset could visually emphasize the signal pattern of that particular subunit. To address this concern, we re-plotted Figure 1C after sorting promoters by nuclear CAGE TPM, which provides an independent measure. The overall pattern of coordinated TAF occupancy remains similar when promoters are sorted by CAGE TPM, indicating that the observed concordance among TFIID subunits is not an artifact of sorting by TAF1 (see figure below).

B

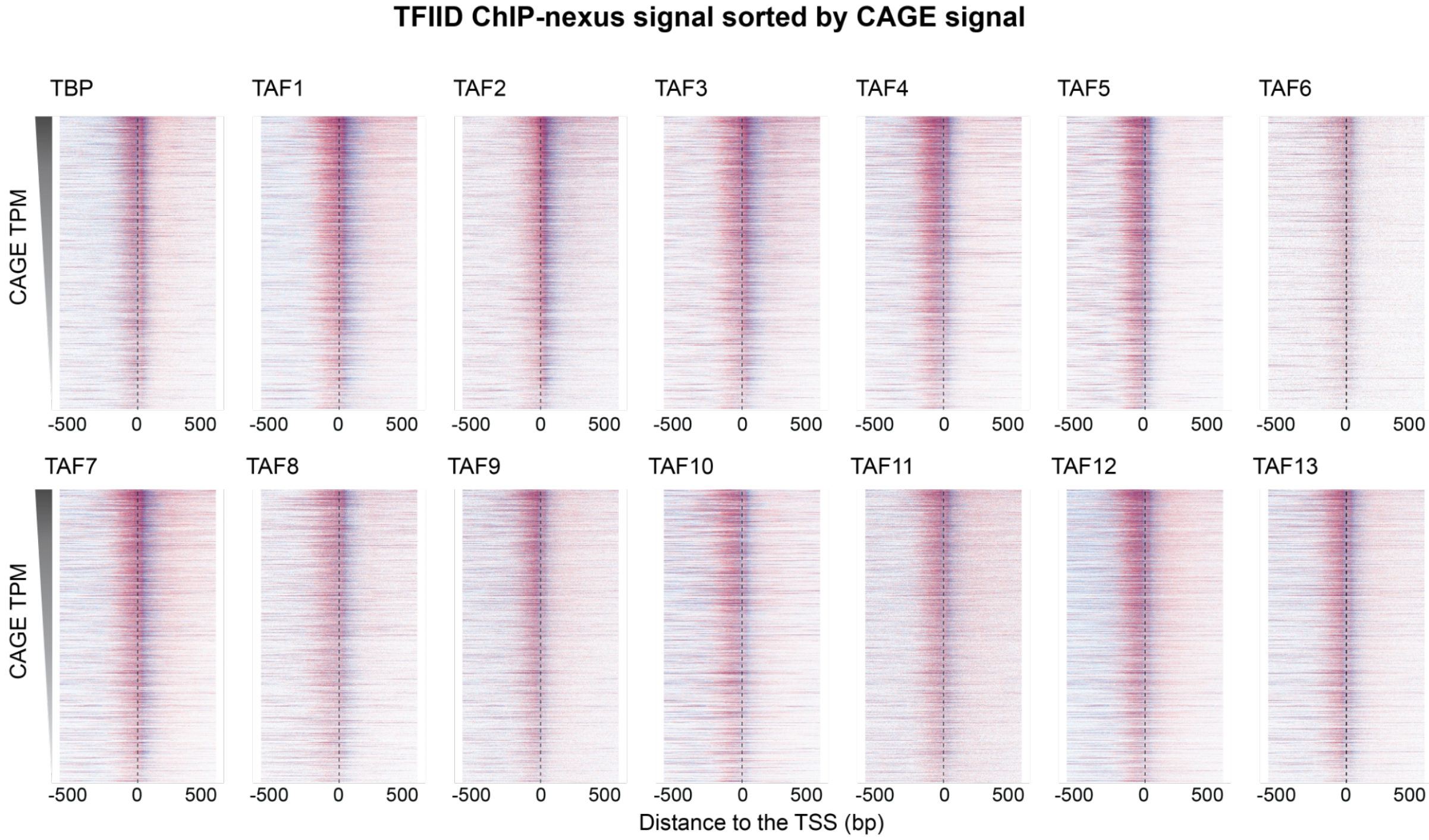

• Line 69-72: the statement on SAGA complex is unclear. The authors indicate that correlations among TAF9/10/12 are not apparent likely due to the TAF1-based order of TSSs. If so, it would be relatively easy to show their correlation using an alternative sorting. However, they do not show any evidence for preferred co-occupancy of TAF9/10/12. They should either provide supporting analyses or remove the statements to avoid confusion.

We thank the reviewer for this suggestion. We agree that our original statement was overstated relative to the data presented. Accordingly, we have removed the original statement to avoid overinterpretation. We now limit our description to the observed occupancy patterns directly supported by the data.

• In Fig. S3, both dotted line and solid line are shown. The legend should indicate what they represent.

We thank the reviewer for pointing this out. The dotted line represents the division between narrow and broad promoters. We have updated the figure legend accordingly.

• In Fig. 2c, the authors appear to add an annotation “TAF3?” based on their prediction. Since this structure is used to validate that ChIP-nexus can support structural inference, it would be more appropriate to keep the original annotations from the source study. Also, the yeast module does not help here so it can be removed.

We thank the reviewer for this helpful suggestion. We agree that the “TAF3?” annotation was speculative and could give the impression that the structural assignment was derived directly from the cryo-EM study. To avoid potential confusion, we have removed this annotation and now retain the original labeling from the source structure. In addition, we have simplified the

panel by removing the yeast module inset, which was not essential for interpreting the structural comparison. The figure and legend have been updated accordingly.

• The paragraph starting from Line 209 seems to be combined with the previous paragraph.

We thank the reviewer for pointing this out. We have now combined both paragraphs to improve readability.

• Line 234 should cite relevant previous reports.

We thank the reviewer for this suggestion. We have revised the text to include citations to relevant studies.
